# Direct Synthesis of α‑Amino Ketones via Photochemical Nickel-Catalyzed Acyl–Aryl Cross-Coupling

**DOI:** 10.1021/acsomega.5c08932

**Published:** 2025-10-28

**Authors:** Mariana dos S. Dupim, Gustavo dos S. Martins, Thais G. Silva, Fernanda G. Finelli

**Affiliations:** Instituto de Pesquisas de Produtos Naturais, Universidade Federal Do Rio de Janeiro, Rio de Janeiro 21941-599, Brazil

## Abstract

We report a direct method for synthesizing α-amino arylketones via photochemical nickel-catalyzed acyl–aryl cross-coupling of α-amino acid-derived aldehydes with aryl bromides. The reaction proceeds efficiently under mild conditions, particularly with electron-deficient aryl bromides, and provides mechanistic insights into the competition between ketone formation and decarbonylation pathways. The protocol further enables the straightforward preparation of cathinone derivatives, highlighting its synthetic versatility and potential in medicinal and forensic chemistry.

## Graphical abstract

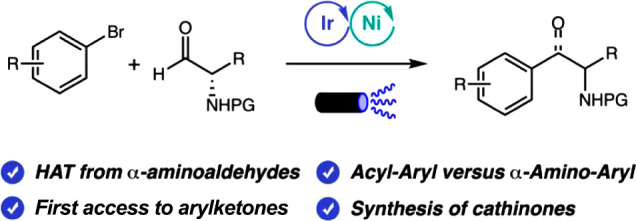

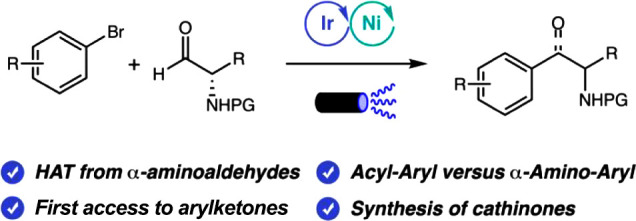

## Introduction

α-Amino ketones are highly significant organic compounds in synthetic and medicinal chemistry, valued for their versatility and wide scope of applications. The high reactivity resulting from the simultaneous presence of an amino group and a ketone at the α-position renders these molecules strategic building blocks for natural products, pharmacologically active compounds, and heterocycles of synthetic interest. These compounds function as key precursors to physiologically important ethanolamine derivatives and as intermediates in the synthesis of diverse heterocyclic systems, including pyrazines and pyrroles, as well as chiral aminoalcohols employed as ligands in asymmetric synthesis. Their significance is further evidenced by clinically relevant α-amino arylketone drugs such as the antidepressant bupropion, and the appetite suppressants amfepramone and pyrovalerone, as well as by their occurrence in natural products like cathinone, a pseudoalkaloid isolated from Khat leaves, known for its central nervous system stimulant properties and widely recognized as a drug of abuse.^1–5^

Given their considerable impact in organic chemistry, α-amino ketones have been the focus of numerous synthetic studies. Traditional approaches include nucleophilic substitution of α-halogenated ketones, electrophilic addition to enolates, and the use of α-amino acids as starting materials, typically requiring protection of the amine group and transformation of the carboxylate into derivatives such as acid chlorides, esters, or amides, in strategies commonly involving organolithium or Grignard additions and Friedel–Crafts acylation. However, these methods often encounter several limitations, including the restricted availability of suitable nitrogen electrophiles, the need for additional steps, and harsh reaction conditions.^6–9^

Over the past decade, dual photoredox-nickel catalysis has emerged as a versatile and sustainable strategy for constructing α-amino ketones, offering advantages such as improved atom economy, stereocontrol, functional group tolerance, and mild reaction conditions (Scheme 1). Seminal contributions include the pioneering metal insertion-decarboxylation-recombination sequence of *in situ*-generated anhydrides, reported by MacMillan and coworkers, which provided racemic α-amino alkylketones, and the complementary α-amino radical acylation approach using pyrrolidines and alkyl anhydrides, developed by Doyle and coworkers.^10,11^ Subsequently, Baran and coworkers expanded these studies by employing redox-active esters as precursor of α-amino radicals and acid chlorides in combination with chiral nickel ligands, thereby achieving enantioselective α-amino ketone synthesis.^12^ In parallel, Murakami and coworkers reported a dehydrogenative alkyl-acyl radical cross-coupling platform, which was later advanced by Huo and coworkers through the development of a highly enantioselective α-aminoacyl radical cross-coupling using amino acid derivatives and aldehydes to furnish enantiopure α-amino ketones.^13,14^ More recently, Hong and coworkers introduced an elegant strategy for coupling chiral amino acid chlorides with unactivated C­(sp^3^)–H hydrocarbons, providing broad access to structurally diverse chiral amino ketones while preserving the stereochemical integrity of the amino acid precursors.^15^ Finally, Stecko and Kobus-Bartoszewicz developed a complementary two-step approach involving cross-coupling, either through the Suzuki reaction with arylboronic acids or via dual photoredox/Ni-catalysis to install alkyl groups, followed by oxidative cleavage to access amino ketones.^16^

**Scheme 1:**
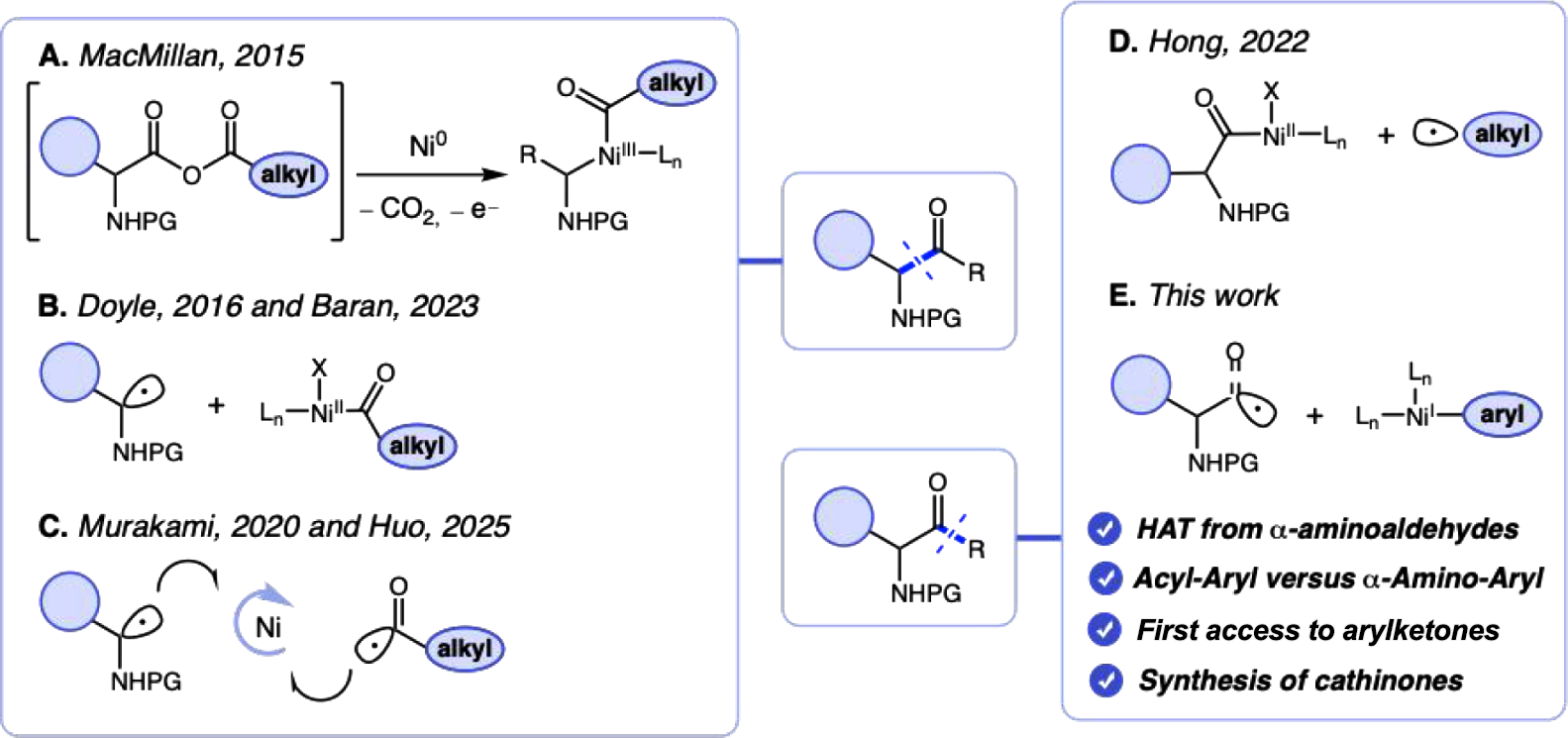
Dual Photoredox-Nickel Catalysis Synthesis of α-Amino Ketones

Despite these advances and the widespread importance of α-amino arylketones in synthetic and medicinal chemistry, photocatalytic methods for accessing these compounds are still underexplored.^17^ Herein, we report a direct approach to α-amino arylketones via photochemical nickel-catalyzed acyl-aryl cross-coupling, employing stable α-amino aldehydes derived from α-amino acids and aryl bromides. Moreover, we investigate how the intrinsic reactivity of the aryl bromide partners dictates the competition between acyl-aryl and α-amino-aryl cross-coupling pathways, aiming to provide mechanistic insights and guide future reaction design.

## Results and Discussion

In the search for an efficient synthesis of α-amino arylketones, we initiated our studies by investigating the metallaphotoredox cross-coupling of α-amino aldehydes with aryl bromides. As a model system, aryl bromide **1a** and the l-alanine derivative **2a** were subjected to reaction with Ir­[dF-(CF_3_)­ppy]_2_(dtbbpy)­PF_6_ under visible light irradiation, in the presence of NiBr_2_ *·*dtbbpy, quinuclidine, and K_2_CO_3_ in 1,4-dioxane (Table 1).

**Table 1:**
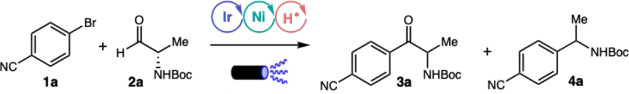
Optimization of the Reaction Conditions

entry	deviations from standard conditions^a^	3a^b^	4a^b^	1a recov.^b^
1	none	23%	22%	18%
2	30 mol % quinuclidine	14%	30%	5%
3^c^,^d^	50 mol % DABCO, NaHCO_3_	16%	23%	10%
4	no quinuclidine	32%	20%	14%
5^c^	NiBr_2_·2,2’-bpy instead of NiBr_2_·dtbbpy	n.d.	n.d.	72%
6^c^	NiBr_2_·BINAP instead of NiBr_2_·dtbbpy	n.d.	n.d.	75%
7^c^	Ni(acac)_2_ instead of NiBr_2_·DME	n.d.	n.d.	80%
8^c^	NiBr_2_·1,10-phen instead of NiBr_2_·dtbbpy	n.d.	n.d.	49%
9^c^	30 mol % NiBr_2_·dtbbpy	22%	18%	n.d.
10^c^	NaBr (20 mol %)	37%	4%	10%
11^c^	NaBr (50 mol %)	44%	15%	5%
12^c^	TBAB (20 mol %)	10%	n.d.	60%
13^c^	LiCl (20 mol %)	24%	10%	5%
14^c^	3 equiv of **2a**	40%	14%	6%
15^c^	4 equiv of **2a**	42%	18%	5%
16^c^	1 equiv of **2a** and 2 equiv of **1a**	34%	10%	25%
17^c^	iodobenzene instead **1a** and NaBr (20 mol %)	n.d.	n.d.	96% (PhI)

a Reaction conditions: **1a** (0.11 mmol), **2a** (0.22 mmol), Ir­[dF­(CF_3_)­ppy]_2_(dtbbpy)­PF_6_ (1 mol %), NiBr_2_·DME (10 mol %), dtbbpy (10 mol %), quinuclidine (10 mol %), K_2_CO_3_ (1.5 equiv), dioxane (0.03 M) under 10 W blue LED irradiation for 24 h at room temperature.

b Determined by ^1^H quantitative NMR using 1,3-benzodioxole as the internal standard.

c No quinuclidine.

d No K_2_CO_3_.

Under the standard conditions, the arylketone **3a** was obtained along with an equimolar amount of the decarbonylated product **4a** (Table 1, entry 1). Since α-heteroatom substituents are known to accelerate the decarbonylation of acyl radical intermediates,^18,19^ these findings were nevertheless encouraging and motivated us to explore strategies to modulate this ratio in favor of ketone formation.

To further investigate, we examined the role of the HAT catalyst and observed that, in its absence, the reaction proceeded more efficiently, affording the ketone in slight excess (Table 1, entries 2–4). Although this transformation had previously been shown to occur without an external HAT catalyst, this is the first instance in which improved efficiency was achieved under such conditions. Based on previous observations, we hypothesized that the bromine radical, generated via Ni–Br bond homolysis, could act as an intrinsic HAT catalyst in the absence of quinuclidine or DABCO.^20,21^

Therefore, we decided to evaluate the influence of critical reaction parameters that can affect nickel activity and deepen our understanding of the decarbonylation process. Alternative nickel ligands failed to promote product formation (Table 1, entries 5–8), and increasing the nickel catalyst loading did not lead to higher yields (Table 1, entry 9). In contrast, the use of additives capable of facilitating the HAT step proved advantageous (Table 1, entries 10–13). Notably, the presence of NaBr significantly enhanced overall reaction efficiency and shifted selectivity toward ketone formation over decarbonylation. Likewise, higher aldehyde concentrations also increased efficiency and further favored the ketone product (Table 1, entries 13–15). The use of the α-amino aldehyde as the limiting reagent also afforded good results; however, no recovery of this substrate was possible, and the resulting crude mixture was considerably more complex, rendering purification substantially more challenging (Table 1, entry 16). Moreover, employing a more reactive aryl iodide did not lead to any product formation (Table 1, entry 17).

Having these insights in mind, we next evaluated the influence of the aryl bromide on the competition between the acyl–aryl and α-amino-aryl cross-coupling pathways (Scheme 2). The reaction proceeded in good to excellent yields with electron-deficient aryl bromides bearing nitrile, acetyl, trifluoromethyl, and methyl sulfone substituents (**1a**–**f**, 43–73%), affording mixtures of ketones **3** and decarbonylated products **4** with favorable selectivity toward ketone formation. In contrast, electron-rich and electron-neutral arenes provided significantly lower yields (**1g**–**i**, 12–40%), requiring higher equivalents of aldehyde to improve coupling efficiency. Interestingly, under these conditions, the ketone-to-decarbonylated product ratio increased dramatically (>20:1).

**Scheme 2:**
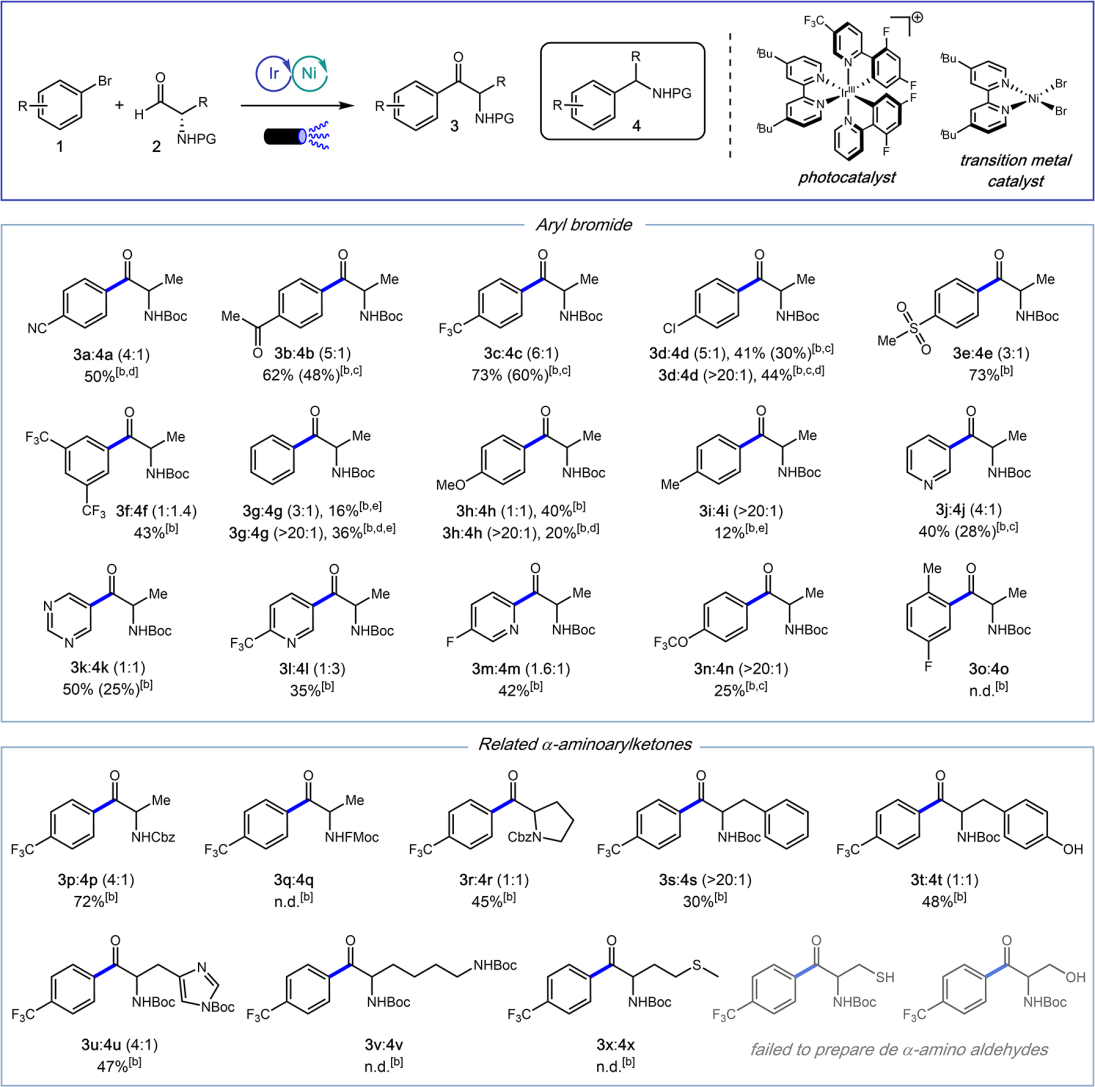
Exploration of Substrate Scope^a^ ^b^ ^c^ ^d^ ^e^

a Reactions performed with aryl bromide (0.11 mmol), α-amino aldehyde (2 equiv), Ir­[dF­(CF_3_)­ppy]_2_(dtbbpy)­PF_6_ (1 mol %), NiBr_2_·dtbbpy (10 mol %), NaBr (20 mol %), K_2_CO_3_ (1.5 equiv), in dioxane (0.03 M) under 10 W blue LED irradiation for 24 h at room temperature.
b Yield of the mixture of **3** + **4** determined by ^1^H quantitative NMR using 1,3-benzodioxole as the internal standard.
c Isolated yield of **3** in parentheses.
d Reactions performed with 3 equiv of α-amino aldehyde.
e Reactions performed with 10 mol % of quinuclidine.

We next investigated heteroaryl bromides, which are frequently found in bioactive molecules. Although the yields were moderate (**1j**–**m**, 35–50%), no significant preference between ketone and decarbonylated products was observed, typically resulting in 1:1 mixtures, except for 3-bromopyridine (**1j**). Remarkably, the aryl bromide **1d** bearing a *p*-chloro substituent underwent selective functionalization exclusively at the bromide position, highlighting the potential for further structural diversification.

We also examined the tolerance and efficiency of the reaction with other amino-protecting groups. The alanine-Cbz derived aldehyde **2p** afforded the desired ketone in good yield (**3p**:**4p** 4:1, 72%), whereas the alanine-Fmoc derived aldehyde **2q** failed to deliver the coupling products.

Finally, a representative set of amino acids was selected, including other aliphatic (Pro), aromatic (Phe, Tyr), basic (His, Lys), hydroxylic (Ser), and sulfur-containing (Cys, Met), to evaluate their compatibility with our strategy. Amino aldehydes derived from Pro, Phe, Tyr, His, Lys, and Met were successfully synthesized, whereas those derived from Ser and Cys could not be obtained.

The proline- and tyrosine-derived aldehydes afforded the corresponding ketones **3r** and **3t** together with an equimolar amount of the decarbonylated products **4r** and **4t**. The phenylalanine-derived aldehyde **2s** furnished exclusively the desired ketone **3s**, while the histidine-derived aldehyde **2u** predominantly yielded ketone **3u**. In contrast, aldehydes derived from lysine **2t** and methionine **2x** did not afford the expected products under the standard conditions.

In addition to assessing the influence of the electronic nature of the aryl bromide on both reaction efficiency and the extent of decarbonylation, our control studies also revealed that ketone **3** undergoes racemization (see Supporting Information, Section 8).

Based on previous studies, we propose the mechanism illustrated in Scheme 3.^22,23^ Initial visible-light excitation of the iridium­(III) photocatalyst Ir­[dF­(CF_3_)­ppy]_2_(dtbbpy)­PF_6_ generates the long-lived excited-state *Ir­(III). This highly oxidizing species can undergo single-electron transfer (SET) with the bromide anion, producing the reduced Ir­(II) complex along with the electrophilic bromine radical. The bromine radical can subsequently abstract a hydrogen atom from the aldehyde **2**, forming the nucleophilic acyl radical **2’**. Concurrently, the Ni(0) catalyst undergoes oxidative addition into the aryl bromide, generating the **A**-Ni­(II) intermediate. Trapping the acyl radical **2’** by **A**-Ni­(II) affords the highly electrophilic **B**-Ni­(III) complex, which undergoes reductive elimination to yield the target ketone **3** and a **D**-Ni­(I) intermediate. Finally, SET from the strongly reducing Ir­(II) species regenerates the Ni(0) catalyst, releasing the bromide anion and closing the photoredox cycle. Alternatively, the acyl radical **2’** may undergo decarbonylation to generate an alkyl radical **2″**, which can also be trapped by the **A**-Ni­(II) species to form a **C**-Ni­(III) complex. Reductive elimination of this complex yields the decarbonylated product **4**.

**Scheme 3:**
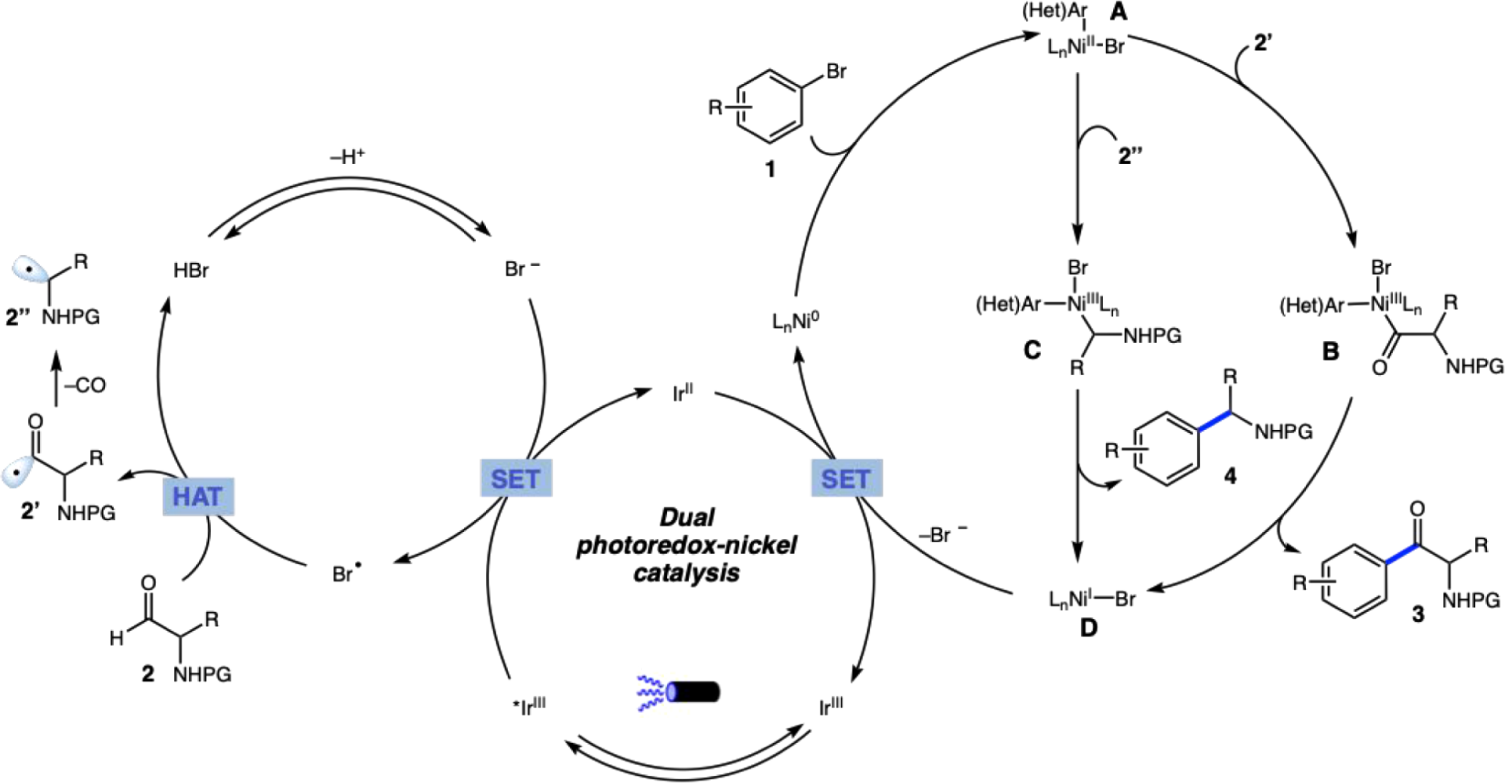
Proposed Reaction Mechanism for HAT-Metallaphotoredox CH-Arylation Employing α-Amino Aldehydes

Based on recent studies of Cavallo, Gagliardi and coworkers,^24^ an alternative catalytic cycle involving Ni­(II) and Ni­(I) species has been also proposed (see Supporting Information, Section 9). In this mechanism, the LNi^II^(Ar)Br complex is excited via energy transfer from the light-excited Ir photocatalyst, leading to Ni–Br bond homolysis and formation of a bromine radical. This radical promotes the hydrogen atom transfer (HAT) from aldehyde **2** to **2’’**, which is trapped by Ni­(I) and undergoes reductive elimination to yield ketone **3**. Additionally, an in-sphere decarbonylation of the Ni­(II) intermediate may also lead to product **4**.^25^ Further studies are required to substantiate this hypothesis more conclusively.^26–29^

During our studies, we observed that electron-deficient aryl bromides, the use of NaBr as an additive, and an excess of aldehyde significantly enhanced the overall reaction efficiency, favoring ketone formation over the decarbonylated product. Electron-deficient aryl bromides display higher reactivity in the oxidative addition step (Scheme 3), thereby improving the reaction yield. In parallel, electron-withdrawing substituents on the aryl ring render the nickel center in complex **B**Ni­(III) even more electrophilic, facilitating the reductive elimination step. This effect likely accelerates the cross-coupling pathway, making it preferred over the decarbonylation (**2’** to **2’’**). Higher aldehyde concentrations accelerate radical addition to the **A**-Ni­(II) intermediate, probable improving the kinetics of ketone **3** formation. NaBr, in turn, plays a crucial role in the hydrogen atom transfer step of the proposed mechanism.

Finally, to further demonstrate the utility of our methodology, selected α-amino arylketones were directly converted into the corresponding cathinone hydrochlorides **5** in a simple one-step procedure. Treatment with 4 M HCl in dioxane at room temperature for 2 h furnished the pure hydrochloride salts in excellent yields, without the need for further purification (Scheme 4). Considering that cathinone derivatives are widely known as drugs of abuse, this direct and efficient approach can also assist in the synthesis of reference materials, which are highly valuable in forensic chemistry.

**Scheme 4:**
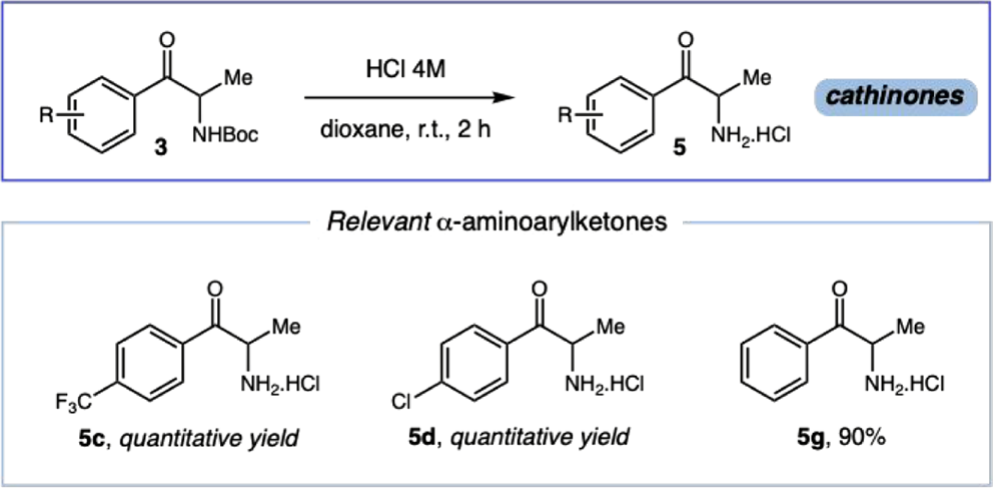
Synthesis of Relevant Cathinones

## Conclusions

In conclusion, we have developed the first nickel-catalyzed photochemical strategy for the direct synthesis of α-amino arylketones from readily accessible α-amino acid-derived aldehydes and aryl bromides. This method demonstrates that ketone formation is feasible under a HAT-based photochemical approach with α-amino aldehyde, challenging previous assumptions in the literature.

Our results highlight the crucial role of the electronic nature of aryl bromides in dictating the competition between acyl–aryl coupling and decarbonylation, while also suggesting a new mechanistic pathway for these transformations. Ongoing experimental and computational studies will further elucidate the reaction pathway, with the aim of developing strategies to control selectivity and mitigate racemization. Notably, leveraging decarbonylation as a strategy for C–C bond formation significantly expands the synthetic scope of this methodology, which could be further optimized using CO-scavenging additives, for example.

Altogether, these insights not only advance our mechanistic understanding but also open new avenues for designing powerful synthetic strategies, expanding access to valuable molecular architectures.

## Experimental Section

### General Information

All commercially available reagents were used as received or were purified according to reported procedures. Reactions involving anhydrous conditions were done under an argon atmosphere. Light-driven reactions were performed using a 10 W blue LED. Flash chromatography was performed on silica gel 60 (200–400 mesh) and thin layer chromatography was performed on Silicycle TLC plates precoated with silica gel 60 F254 using UV light as the visualizing agent or ethanolic phosphomolybdic acid and heating as developing agents. Eluents used for flash chromatography are described in each experimental procedure. NMR spectra were obtained on a Varian VNMRS 500 (499.90 MHz for ^1^H; 125.70 MHz for ^13^C), Varian Inova 400 (399.96 MHz for ^1^H; 100.57 MHz for ^13^C) spectrometer. ^1^H NMR chemical shifts are reported in parts per million (ppm) relative to TMS, with the residual solvent peak used as an internal reference. Multiplicities are reported as follows: singlet (s), doublet (d), doublet of doublets (dd), doublet of doublets of doublets (ddd), doublet of triplets (dt), triplet (t), quartet (q), quintet (quin), multiplet (m), and broad resonance (br). HRMS data were obtained on a microTOF-II Bruker mass spectrometer. Enantiomeric excesses were determined on a Shimadzu Prominence LC-20A equipped with a PDA detector, using a Lux 5 mm Cellulose-2 LC column (250 mm x 4.6 cm).

### Synthesis of Substrate and Photocatalyst

The α-amino aldehydes **2** ^30,31^ and photocatalyst [Ir­(dF­(CF_3_)­ppy)_2_(dtbbpy)]­(PF_6_)^32^ were synthesized according to previously reported procedures.

### Procedure for Optimization of Acyl–Aryl Coupling

To a 2-dram clear vial were added nickel catalyst (0.012 mmol, 0.11 equiv), ligand (0.012 mmol, 0.11 equiv), and dioxane (3 mL). The mixture was sonicated for 15 min until a clear solution was obtained. In another 2-dram clear vial equipped with a magnetic stirring bar, the photocatalyst (Ir­[dF­(CF_3_)­ppy]_2_(dtbbpy))­PF_6_, 1.2 mg, 0.0011 mmol, 1 mol %), aldehyde **2** (2–4 equiv), HAT catalyst, K_2_CO_3_ (22 mg, 0.165 mmol, 1.5 equiv), bromobenzonitrile (**1a**, 20 mg, 0.11 mmol, 1 equiv), and additive were added. The nickel catalyst solution was then transferred to the vial containing the other reagents. This vial was sealed with a rubber septum and sparged with argon for 15 min. The reaction was irradiated with one blue LED (1 cm distance from the light source) under stirring for 20 h at room temperature. Afterward, 13 μL of 1,3-benzodioxole (0.11 mmol) was added to the reaction vial as an internal standard. The solution was stirred for 2 min, filtered through Celite, and analyzed by ^1^H NMR to determine crude yield.

### General Procedure for Synthesis of α-Amino Ketones **3a**–**3x**

To a 2-dram clear vial were added NiBr_2_·DME (4 mg, 0.01 mmol, 0.1 equiv), 4,4′-di*tert*-butyl-2,2′-dipyridyl (*dtbbpy*, 3 mg, 0.01 mmol, 0.1 equiv), and dioxane (3 mL). The mixture was sonicated for 15 min until a clear yellowish solution was obtained. In another 2-dram clear vial equipped with a magnetic stirring bar, the photocatalyst (Ir­[dF­(CF_3_)­ppy]_2_(dtbbpy))­PF_6_, 1.2 mg, 0.0011 mmol, 1 mol %), aldehyde **2** (1–3 equiv), K_2_CO_3_ (22 mg, 0.165 mmol, 1.5 equiv), aryl bromide if solid (0.11 mmol, 1 equiv), and NaBr (2.3 mg, 0.02 mmol, 0.2 equiv), for reactions with electron poor aryl bromides **1**, or quinuclidine (1.2 mg, 0.01 mmol, 0.1 equiv), for reactions with electron rich aryl bromides **1**, were added. The nickel catalyst solution was then transferred to the vial containing the other reagents. This vial was sealed with a rubber septum and sparged with argon for 15 min. If the aryl bromide is volatile, it should be added only after the solution has been sparged. The reaction was sealed with parafilm and irradiated with one 10 W blue LED (at 1 cm distance from the light source) under stirring for 20 h at room temperature. After that time, 13 μL of 1,3-benzodioxole (0.11 mmol) was added to the reaction vial as an internal standard. The solution was stirred for 2 min, filtered through Celite, and analyzed by ^1^H NMR to determine crude yield. The product was isolated by flash column chromatography to afford the ketone or ketone/decarbonylated mixture as specified for each product.

## Supplementary Material



## Notes

General information, preparation of photocatalyst [Ir­(dF­(CF_3_)­ppy)_2_(dtbbpy)]­(PF_6_); synthesis of α-amino aldehydes; optimization of the reaction conditions for the synthesis of α-amino aryl ketones; synthesis of α-amino aryl ketones from *N*-Boc-l-alanine; synthesis of α-amino aryl ketones from other α-amino aldehydes; synthesis of cathinones derivatives; chiral HPLC analysis; alternative cycle pathway for acyl–aryl cross-coupling from α-aminoaldehydes; spectra (PDF)
