# Supplementary material for: Direct Synthesis of α‑Amino Ketones via Photochemical Nickel-Catalyzed Acyl–Aryl Cross-Coupling
Source: ACS Omega. 2025 Oct 28;10(44):53479–85. doi: 10.1021/acsomega.5c08932 (PMC12612937; doi:10.1021/acsomega.5c08932)
Supplement: Supplementary file 1 [file ao5c08932_si_001.pdf]

Supporting Information for:

Direct Synthesis of α-Amino Ketones via Photochemical Nickel-Catalyzed Acyl-Aryl Cross Coupling

Mariana dos S. Dupim[a], Gustavo dos S. Martins[a], Thais G. Silva,[a] and Fernanda G. Finelli[a]*

[a]Instituto de Pesquisas de Produtos Naturais, Universidade Federal do Rio de Janeiro, Rio de Janeiro 21941-599, Brazil

*Corresponding author: finelli@ippn.ufrj.br

Table of contents

1. General information S2
2. Preparation of photocatalyst [Ir(dF(CF$_3$)ppy)$_2$(dtbbpy)](PF$_6$).. S2
3. Synthesis of α-amino aldehydes S4
4. Optimization of the reaction conditions for the synthesis of α-amino aryl ketones.. S9
5. Synthesis of α-amino aryl ketones from *N*-Boc-L-alanine.. S12
6. Synthesis of α-amino aryl ketones from other α-amino aldehydes S19
7. Synthesis of cathinones derivatives S22
8. Chiral HPLC analysis.. S24
9. Alternative Cycle pathway for Acyl-Aryl Cross-Coupling from α-aminoaldehydes S27
10. Spectra.. S28

1. General information

All commercially available reagents were used as received or were purified according to reported procedures. Reactions involving anhydrous conditions were done under an argon atmosphere. Light-driven reactions were performed using a 10 W blue LED.

Flash chromatography was performed on silica gel 60 (200–400 mesh) and thin layer chromatography was performed on Silicycle TLC plates pre-coated with silica gel 60 F254 using UV light as the visualizing agent or ethanolic phosphomolybdic acid and heating as developing agents. Eluents used for flash chromatography are described in each experimental procedure.

NMR spectra were obtained on a Varian VNMRS 500 (499.90 MHz for ^{1}H; 125.70 MHz for ^{13}C), Varian Inova 400 (399.96 MHz for ^{1}H; 100.57 MHz for ^{13}C) spectrometer. ^{1}H NMR chemical shifts are reported in parts per million (ppm) relative to TMS, with the residual solvent peak used as an internal reference. Multiplicities are reported as follows: singlet (s), doublet (d), doublet of doublets (dd), doublet of doublets of doublets (ddd), doublet of triplets (dt), triplet (t), quartet (q), quintet (quin), multiplet (m), and broad resonance (br).

HRMS data were obtained on a microTOF-II Bruker mass spectrometer. Enantiomeric excesses were determined on a Shimadzu Prominence LC-20A equipped with a PDA detector, using a Lux 5 mm Cellulose-2 LC column (250 mm x 4.6 cm).

Irradiation setup: Reactions were carried out using a blue light lamp with a peak emission in the 450-460 nm range. The light source was manually set to a power of 10 W equipped with a cooling fan to maintain the temperature at 25 ºC, monitored and controlled via a temperature-regulated vial.

2. Preparation of photocatalyst $[Ir(dF(CF_3)ppy)_2(dtbbpy)](PF_6)$[1]

2.1 - Synthesis of ligand 2-(2,4-difluorophenyl)-5-(trifluoromethyl)pyridine

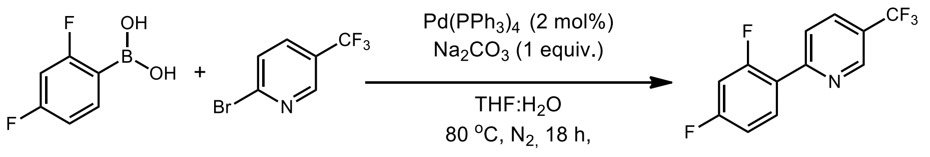

To a round-bottom flask containing a magnetic stirring bar were added 2,4-difluorophenylboronic acid (3.84 g, 24.3 mmol, 1.1 equiv.), 2-bromo-5-(trifluoromethyl)pyridine (5.0 g, 22.1 mmol, 1.0 equiv.), Na_2CO_3 (2.34 g, 22.1 mmol, 1.0 equiv.) and $Pd(PPh_3)_4$ (0.51 g, 0.44 mmol, 0.02 equiv.). The flask was evacuated and filled with argon three times, THF (70 mL) and water (22 mL) were added, and the reaction mixture was stirred under reflux for 18 hours. After that, the reaction mixture was concentrated under vacuum, diluted with CH_2Cl_2,

[1]Lowry, M. S.; Goldsmith, J. I.; Slinker, J. D.; Rohl, R.; Pascal, R. A.; Malliaras, G. G.; Bernhard, S. *Chem. Mater.* **2005**, *17,* 5712–5719.

and washed with water and brine. The organic phase was dried with $MgSO_4$, concentrated in vacuum, and purified by flash column chromatography using 5% CH_2Cl_2/Hexanes to furnish the product as a crystalline colorless solid in 92% yield (5.27 g).

^{1}H NMR (500 MHz, CDCl$_3$) δ 8.96 (s, 1H), 8.12 – 8.07 (m, 1H), 7.99 (dd, *J* = 8.4, 2.2 Hz, 1H), 7.91 (d, *J* = 8.3 Hz, 1H), 7.06 – 7.03 (m, 1H), 6.95 (ddd, *J* = 11.3, 8.7, 2.5 Hz, 1H).
^{13}C NMR (126 MHz, CDCl$_3$) δ 164.0 (dd, *J* = 252.9; 12.2 Hz); 161.1 (dd, *J* = 253.7; 12.0 Hz); 155.9 (s), 146.7 (q, *J* = 4.0 Hz); 133.9 (q, *J* = 3.3 Hz); 132.6 (dd, *J* = 9.8; 4.1 Hz); 125.3 (q, *J* = 33.1 Hz); 123.8 (d, *J* = 10.8 Hz; 123.7 (q, *J* = 272.2 Hz); 122.6 (dd, *J* = 11.4; 3.8 Hz); 112.4 (dd, *J* = 21.2; 3.6 Hz); 104.8 (dd, *J* = 26.8; 25.5 Hz).

2.2 - Synthesis of photocatalyst [Ir(dF(CF$_3$)ppy)$_2$(dtbbpy)](PF$_6$)[1]

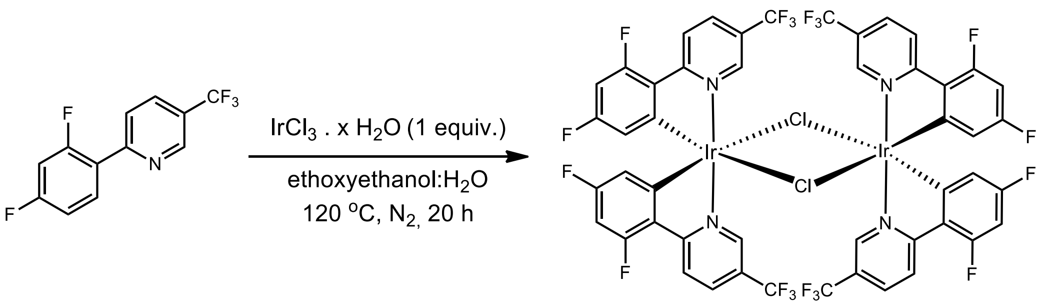

Step 1: To a round-bottom flask equipped with a magnetic stirring bar were added 2-(2,4-difluorophenyl)-5-(trifluoromethyl)pyridine (4.9 g, 18.8 mmol, 2.2 equiv.), $IrCl_3 \cdot H_2O$ (2.71 g, 8.5 mmol, 1.0 equiv.), ethoxyethanol (100 mL), and water (50 mL). The flask was equipped with a condenser, sealed with a rubber septum and the reaction mixture was sparged with nitrogen under stirring for 30 minutes. After that, the reaction was heated to 120 °C and stirred for 20 hours. Then, 200 mL of water was added. The precipitated solid was filtered and washed with diethyl ether, furnishing the complex [(dF(CF$_3$)ppy)$_2$-Ir-μ-Cl]$_2$ as a yellow solid (4.36 g) in 69% yield. The product was used in the next step without further purification.

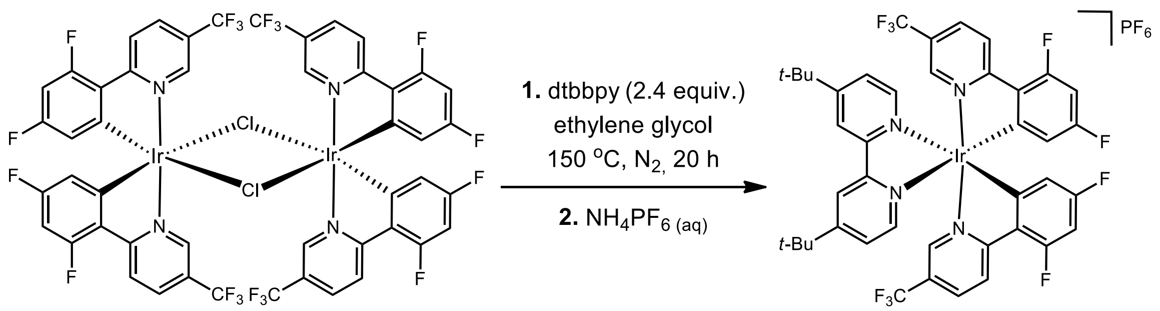

Step 2: To a round-bottom flask equipped with a magnetic stirring bar were added complex [(dF(CF$_3$)ppy)$_2$-Ir-μ-Cl]$_2$ (2.0 g, 1.34 mmol, 1.0 equiv.), 4,4'-di-*tert*-buthyl-2,2'-dipyridyl (dtbbpy, 0.505 g, 3.22 mmol, 2.4 equiv.) and ethylene glycol (80 mL). The flask was equipped with a condenser, sealed with a rubber septum and the mixture was sparged with nitrogen. The reaction was stirred under nitrogen atmosphere at 150 °C for 20 hours. The crude mixture was diluted with water (120 mL) and extracted three times with hexanes. The

aqueous phase was stirred at 85 °C for 5 minutes to remove any hexanes left. Then, an aqueous solution of NH_4PF_6 (15 g in 150 mL H_2O) was added, and the precipitate was filtered and washed with water and diethyl ether. Purification by recrystallization in acetone/pentane, furnished the product as a yellow solid (1.03 g) in 65% yield.

^{1}H NMR (400 MHz, $(CD_3)_2CO$) δ 8.93 (d, *J* = 1.8 Hz, 2H), 8.61 (dd, *J* = 8.8, 2.4 Hz, 2H), 8.40 (d, *J* = 8.8; 1.9 Hz, 2H), 8.18 (d, *J* = 5.9 Hz, 2H), 7.92 – 7.73 (m, 4H), 6.86 (ddd, *J* = 12.7; 9.4; 2.3 Hz, 2H), 5.97 (dd, *J* = 8.4; 2.3 Hz, 2H), 1.43 (s, 18H).

3. Synthesis of α-amino aldehydes

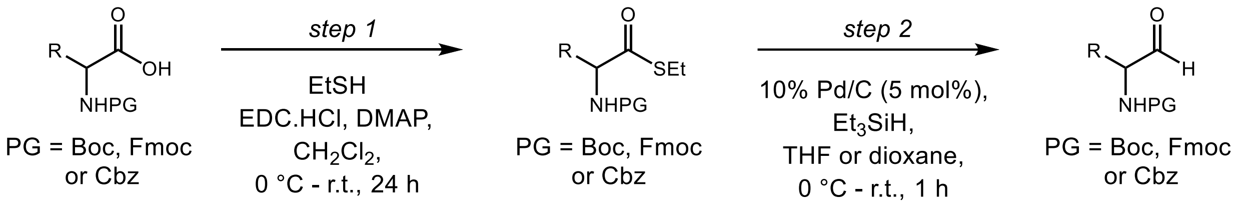

3.1 -Step 1: To a round-bottom flask equipped with a magnetic stirring bar, the amino acid (10 mmol, 1 equiv.), DMAP (122 mg, 1 mmol, 0,1 equiv.), and CH_2Cl_2 (30 mL) were added. Then, at 0 °C, EDC.HCl (2.30 g, 12 mmol, 1.2 equiv.) was added to the reaction flask. The flask was evacuated, backfilled with argon, and the mixture was stirred at 0 °C for 5 minutes. After this period, ethanethiol (2.20 mL, 30 mmol, 3 equiv.) was added, and the reaction was stirred for 24 hours at room temperature. Then, crude mixture was washed with brine (3 x 20 mL), dried with Mg_2SO_4 and concentrated under vacuum. Following this, the crude material was purified by flash column chromatography using a gradient elution (0-30% ethyl acetate in hexanes). The α-amino thioesters from *N*-Boc-L-alanine and D-alanine were isolated in 61% yield; from *N*-Cbz-L-alanine and D-alanine were isolated, respectively, in 50% yield and 55% yield; from *N*-Fmoc-L-alanine was prepared according to this procedure without DMAP, furnishing thioester in 45% yield; from *N*-Boc-L-phenylalanine was isolated in 65% yield; from $(N\text{-Boc})_2$-L-lysine was isolated in 50% yield; from *N*-Cbz-D-proline was isolated in 62% yield; from *N*-Boc-L-methionine was isolated in 81% yield, from *N*-Boc-L-tyrosine was isolated in 53% yield; from $(N\text{-Boc})_2$-L-histidine was isolated in 50% yield.

3.2 - Step 2: To a round-bottom flask equipped with a magnetic stirring bar, α-amino thioester (1 mmol, 1 equiv.), 10% Pd/C (0.05 equiv.), and THF or dioxane (1 mL) were added. The flask was sealed with a rubber septum, evacuated, backfilled with argon, and cooled to 0 °C in an ice bath. Then, Et_3SiH (3 equiv.) was added, and the mixture was stirred for 1 hour. The crude mixture was filtered through celite, concentrated under vacuum, and purified by flash column chromatography using a gradient elution as specified for each aldehyde.

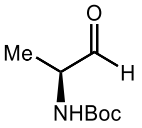

***tert*-butyl (*S*)-(1-oxopropan-2-yl)carbamate** (**2a**)

Prepared according to the General Procedure described in 3.2 *step 2*, using *S*-ethyl (*S*)-2-((tert-butoxycarbonyl)amino)propanethioate (233 mg, 1 mmol, 1 equiv.). The α-amino aldehyde was purified by flash column chromatography using a gradient elution (10-30% ethyl acetate in hexanes), affording compound **2** in 73% yield (126 mg) as a white solid.

^{1}H NMR (400 MHz, $CDCl_3$) δ 9.55 (s, 1H), 5.12 (s, 1H), 4.21 (s, 1H), 1.44 (s, 9H), 1.32 (d, *J* = 7.4 Hz, 3H).

^{13}C NMR (101 MHz, $CDCl_3$) δ 199.9, 155.5, 80.2, 55.7, 28.4, 15.0.

HRMS (ESI) m/z: [M+Na]$^+$ Calcd. for $C_8H_{15}NO_4Na$ 212.0893; Found 212.0873.

$[\alpha]_D^{21}$ = –22.40° (c=1.3 in acetone)

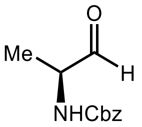

benzyl (*S*)-(1-oxopropan-2-yl)carbamate (**2p**)

Prepared according to the General Procedure described in 3.2 *step 2*, using *S*-ethyl (*S*)-2-(((benzyloxy)carbonyl)amino)propanethioate (267 mg, 1 mmol, 1 equiv.). The α-amino aldehyde was purified by flash column chromatography using a gradient elution (10-50% ethyl acetate in hexanes), affording compound **2p** in 48% yield (98 mg) as a white solid.

^{1}H NMR (400 MHz, $CDCl_3$) δ 9.58 (s, 1H), 7.40 – 7.34 (m, 5H), 5.38 (br s, 1H), 5.13 (s, 2H), 4.34-4.31(m, 1H), 1.38 (d, *J* = 7.4 Hz, 3H).

^{13}C NMR (126 MHz, $CDCl_3$) δ 199.2, 156.0, 136.2, 128.7, 128.4, 128.2, 96.3, 67.2, 14.9.

HRMS (ESI) m/z: [M+K]$^+$ Calcd. for $C_{11}H_{13}NO_4K$ 262.0476; Found 262.0491.

$[\alpha]_D^{21}$ = –1.68° (c=1.4 in acetone)

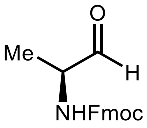

(9H-fluoren-9-yl)methyl (*S*)-(1-oxopropan-2-yl)carbamate (2q)

Prepared according to the General Procedure described in 3.2 *step 2*, using *S*-ethyl (*S*)-2-((((9H-fluoren-9-yl)methoxy)carbonyl)amino)propanethioate (134 mg, 0.38 mmol, 1 equiv.). The α-amino aldehyde was purified by flash column chromatography using a gradient elution (10-30% ethyl acetate in hexanes), affording compound **2q** in 71% yield (80 mg) as a white solid.

^{1}H NMR (500 MHz, CDCl3) δ 9.57 (s, 1H), 7.77 (d, *J* = 7.6, 2H), 7.60 (d, J = 7.2 Hz, 2H), 7.41 (t, *J* = 7.5 Hz, 2H), 7.32 (td, *J* = 7.5, 1.2 Hz, 2H), 5.39 (br s, 1H), 4.45 – 4.42 (m, 2H), 4.37 – 4.28 (m, 1H), 4.23 (t, *J* = 6.8 Hz, 1H), 1.39 (d, *J* = 7.4 Hz, 3H).

^{13}C NMR (126 MHz, $CDCl_3$) δ 199.1, 155.9, 141.5, 127.9, 127.2, 125.2, 120.2, 67.2, 56.1, 47.3, 15.0.

HRMS (ESI) m/z: [M+K]$^+$ Calcd. for $C_{18}H_{17}NO_4K$ 350.0789; Found 350.0787.

$[\alpha]_D^{21}$ = –4.77° (c=1.0 in acetone)

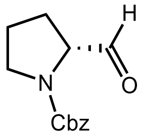

benzyl (*R*)-2-formylpyrrolidine-1-carboxylate (2r)

Prepared according to the General Procedure described in 3.2 *step 2*, using benzyl (*R*)-2-((ethylthio)carbonyl)pyrrolidine-1-carboxylate (293 mg, 1 mmol, 1 equiv.). The α-amino aldehyde was purified by flash column chromatography using a gradient elution (10-40% ethyl acetate in hexanes), affording compound **2r** in 50% yield (116 mg) as a yellowish oil.

^{1}H NMR (400 MHz, CDCl3) δ 9.57 (s, 1H), 9.47 (s, 1H), 7.39 – 7.27 (m, 5H), 5.14 (m, 2H), 4.33 – 4.16 (m, 1H), 3.63 – 3.48 (m, 2H), 2.15 – 1.80 (m, 4H).

^{13}C NMR (126 MHz, $CDCl_3$) δ 200.0, 199.9, 155.4, 154.5, 136.5, 136.2, 128.5, 128.0, 67.3, 65.3, 64.9, 47.3, 46.7, 27.8, 26.6, 24.5, 23.7.

HRMS (ESI) m/z: [M+K]$^+$ Calcd. for $C_{10}H_{17}NO_3K$ 238.0840; Found 238.0825.

$[\alpha]_D^{21}$ = +65.65° (c=1.3 in acetone)

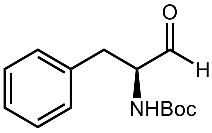

***tert*-butyl (*S*)-(1-oxo-3-phenylpropan-2-yl)carbamate (2s)**

Prepared according to the General Procedure described in 3.2 *step 2*, using *S*-ethyl (*S*)-2-((tert-butoxycarbonyl)amino)-3-phenylpropanethioate (309 mg, 1 mmol, 1 equiv.). The α-amino aldehyde was purified by flash column chromatography using a gradient elution (10-30% ethyl acetate in hexanes), affording compound **2s** in 50% yield (125 mg) as a white solid.

^{1}H NMR (400 MHz, $CDCl_3$) δ 9.63 (s, 1H), 7.31 (t, *J* = 7.2 Hz, 2H), 7.27 – 7.24 (m, 1H), 7.17 (d, *J* = 6.9 Hz, 2H), 5.06 (br s, 1H), 4.46 – 4.39 (m, 1H), 3.14 –3.10 (m, 2H), 1.43 (s, 9H).

^{13}C NMR (126 MHz, $CDCl_3$) δ 199.6, 155.5, 135.9, 129.5, 128.9, 127.2, 80.3, 60.9, 35.6, 28.4.

HRMS (ESI) m/z: $[M+Na]^+$ Calcd. for $C_{14}H_{19}NO_3Na$ 272.1257; Found 272.1262.

$[\alpha]_D^{21}$ = –6.87° (c=1.3 in acetone)

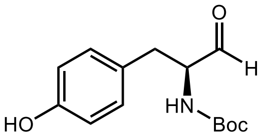

tert-butyl (*S*)-(1-(4-hydroxyphenyl)-3-oxopropan-2-yl)carbamate (2t)

Prepared according to the General Procedure describe in 3.2 *step 2,* using *S*-ethyl (*S*)-2-((tert-butoxycarbonyl)amino)-3-(4-hydroxyphenyl)propanethioate (500 mg, 1.54 mmol, 1 equiv.). The α-amino aldehyde was purified by flash column chromatography using a gradient elution (30-50% ethyl acetate in hexanes), affording compound **2t** in 52% yield (212 mg) as a white solid.

^{1}H NMR (500 MHz, $CDCl_3$) δ 9.60 (s, 1H), 7.00 (m, *J* = 8.0 Hz, 2H), 6.80 – 6.72 (m, 2H), 5.12 (bs, *J* = 7.2 Hz, 1H), 4.41 (bs, *J* = 6.8 Hz, 1H), 3.02 (d, *J* = 6.6 Hz, 2H), 1.44 (s, *J* = 0.9 Hz, 9H).

^{13}C NMR (126 MHz, $CDCl_3$) δ 199.87, 155.75, 130.57, 127.27, 115.83, 99.46, 80.64, 61.05, 34.84.

HRMS (ESI) m/z: $[M+Na]^+$ Calcd. for $C_{14}H_{19}NO_4Na$ 288.1206; Found 288.1203.

$[\alpha]_D^{21}$= –54.33° (c=1.0 in acetone)

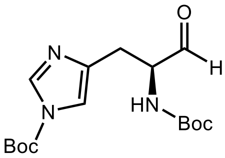

tert-butyl (*S*)-4-(2-((tert-butoxycarbonyl)amino)-3-oxopropyl)-1H-imidazole-1-carboxylate (2u)

Prepared according to the General Procedure describe in 3.2 *step 2,* using tert-butyl (*S*)-4-(2-((tert-butoxycarbonyl)amino)-3-(ethylthio)-3-oxopropyl)-1H-imidazole-1-carboxylate (411 mg, 1.03 mmol, 1 equiv.). The α-amino aldehyde was purified by flash column chromatography using a gradient elution (30-60% ethyl acetate in hexanes), affording compound **2u** in 40% yield (140 mg) as a white solid.

^{1}H NMR (400 MHz, CDCl$_3$) δ 9.67 (s, 1H), 7.98 (d, *J* = 1.3 Hz, 1H), 7.15 (d, *J* = 1.3 Hz, 1H), 5.78 (bs, 1H), 4.40 (m, *J* = 7.9 Hz, 1H), 3.18 – 2.95 (m, 2H), 1.61 (s, 9H), 1.45 (s, 9H).

^{13}C NMR (101 MHz, CDCl$_3$) δ 200.55, 155.83, 146.95, 138.41, 137.01, 114.93, 85.87, 80.21, 59.31, 34.10, 28.44, 28.02.

HRMS (ESI) m/z: [M+H]$^+$ Calcd. for $C_{16}H_{26}N_3O_5$ 340.1867; Found 340.1878.

$[\alpha]_D^{21}$ = –40.03° (c=1.2 in acetone)

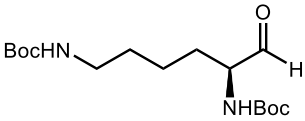

di-tert-butyl (6-oxohexane-1,5-diyl)(*S*)-dicarbamate (**2v**)

Prepared according to the General Procedure described in 3.2 *step 2*, using *S*-ethyl (*S*)-2,6-bis((tert-butoxycarbonyl)amino)hexanethioate (195 mg, 0.5 mmol, 1 equiv.). The α-amino aldehyde was purified by flash column chromatography using a gradient elution (10-50% ethyl acetate in hexanes), affording compound **2t** in 55% yield (91 mg) as a white solid.

^{1}H NMR (400 MHz, CDCl$_3$) δ 9.56 (s, 1H), 5.22 (br s, 1H), 4.60 (br s, 1H), 4.18 (dd, *J* = 10.8, 7.0 Hz, 1H), 3.10 (s, 2H), 1.79 (m, 6H), 1.44 (s, 9H), 1.43 (s, 9H).

^{13}C NMR (101 MHz, CDCl$_3$) δ 200.2, 156.3, 155.7, 80.3, 79.2, 59.9, 40.0, 29.9, 28.8, 28.6, 28.4, 22.3.

HRMS (ESI) m/z: [M+H]$^+$ Calcd. for $C_{16}H_{30}N_2O_5$ for 353.2047; Found 353.2041.

$[\alpha]_D^{21}$ = –22.25° (c=1.0 in acetone)

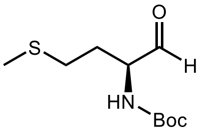

tert-butyl (S)-(4-(methylthio)-1-oxobutan-2-yl)carbamate 2x

Prepared according to the General Procedure describe in 3.2 *step 2,* using (*S*)-2-((tert-butoxycarbonyl)amino)-4-(methylthio)butanethioate (293.4 mg, 1 mmol, 1 equiv.). The α-amino aldehyde was purified by flash column chromatography using a gradient elution (10-30% ethyl acetate in hexanes), affording compound **2x** in 63% yield (150 mg) as a yellowish oil.

^{1}H NMR (400 MHz, $CDCl_3$) δ 9.65 (s, 1H), 5.22 (s, 1H), 4.32 (d, *J* = 8.4 Hz, 1H), 2.57 (td, *J* = 7.1, 3.0 Hz, 2H), 2.29 – 2.18 (m, 1H), 2.09 (s, 3H), 2.00 – 1.87 (m, 1H), 1.46 (d, *J* = 1.0 Hz, 9H).

^{13}C NMR (126 MHz, $CDCl_3$) δ 199.18, 155.11, 80.45, 57.98, 31.36, 28.91, 27.54, 15.53.

HRMS (ESI) m/z: $[M+H]^+$ Calcd. for $C_{10}H_{19}NO_3SNa$ 256.0978; Found 256.0964.

$[\alpha]_D^{21}$ = –25.06º (c=1.2 in acetone)

4. Optimization of the reaction conditions for the synthesis of α-amino aryl ketones

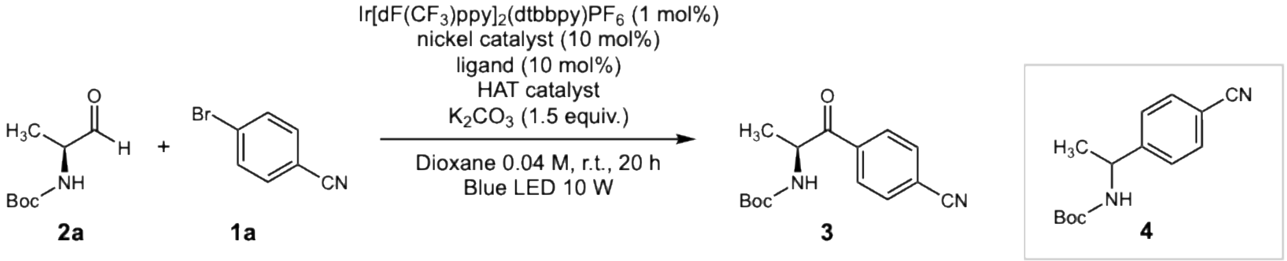

Procedure: To a 2-dram clear vial were added nickel catalyst (0.012 mmol, 0.11 equiv.), ligand (0.012 mmol, 0.11 equiv.), and dioxane (3 mL). The mixture was sonicated for 15 minutes until a clear yellowish solution was obtained. In another 2-dram clear vial equipped with a magnetic stirring bar, the photocatalyst ($Ir[dF(CF_3)ppy]_2(dtbbpy))PF_6$, 1.2 mg, 0.0011 mmol, 1 mol%), aldehyde **2a** (2-4 equiv.), HAT catalyst (table **1**), K_2CO_3 (22 mg, 0.165 mmol, 1.5 equiv.), and bromobenzonitrile (20 mg, 0.11 mmol, 1 equiv.) were added. The nickel catalyst solution was then transferred to the vial containing the other reagents. This vial was sealed with a rubber septum and sparged with argon for 15 minutes. The reaction was irradiated with one 10W blue LED (1 cm distance from the light source) under stirring for 20 hours at room temperature. After that time, 13 µL of 1,3-benzodioxole (0.11 mmol) was added to the reaction vial as an internal standard. The solution was stirred for 2 minutes, filtered through Celite, and analyzed by ^{1}H NMR.

Table S1: Evaluation of HAT catalyst

Entry	HAT catalyst	Yield (%)	decarbonylated product 4 (%)	remaining Aryl-Br 1a (%)
1	**Quinuclidine (10 mol%)**	23	22	18
2	**Quinuclidine (30 mol%)**	14	30	5
3	**DABCO (50 mol%)^b**	16	23	10
4	**no HAT catalyst**	32	20	14

Reaction conditions: **2a** (0.22 mmol), **1a** (0.11 mmol), **PC** (1 mol%), $NiBr_2$.DME (10 mol%), dtbbpy (10 mol%), K_2CO_3 (1.5 equiv.) in dioxane (3 mL) under 10W blue LED irradiation for 24 h. Yields were determined by ^1H NMR using 1,3-benzodioxole as internal standard. $^b NaHCO_3$ (1.5 equiv.) instead K_2CO_3

Preliminary tests (Table S1) were done under previously reported conditions for metallaphotoredox arylation of aldehydes.[2] Surprisingly, after evaluation of the influence of HAT catalysts on reaction, our best result was achieved without the addition of HAT catalyst (entry 4). Based on literature precedents, we speculated that bromine radical generated from Ni-Br bond homolysis could act as HAT catalyst in the absence of quinuclidine or DABCO. Therefore, we decided to evaluate the influence of critical reaction parameters that can affect nickel activity and deeper our understanding of the decarbonylation process (*i.e.*, screening of ligand, nickel catalyst, and additives).

Table S2. Evaluation of nickel catalyst and ligand

Entry	Nickel catalyst and ligand	Yield (%)	decarbonylated product 4 (%)	Remaining Aryl-Br 1a (%)
1	**$NiBr_2$. DME; dtbbpy (30 mol%)**	22	18	n.d.
2	**$NiBr_2$. DME; 2,2'-bpy**	-	-	72
3	**$NiBr_2$. DME; rac BINAP**	-	-	75
4	**$Ni(acac)_2$; dtbbpy**	-	-	80
5	**$NiBr_2$. DME; 1,10-phen**	-	-	49

Reaction conditions: **2a** (0.22 mmol), **1a** (0.11 mmol), **PC** (1 mol%), **ligand** (10 mol%), **nickel** (10 mol%), K_2CO_3 (1.5 equiv.) in dioxane (3 mL) under 10W blue LED irradiation for 24 h. Yields were determined by ^1H NMR using 1,3-benzodioxole as internal standard.

[2] Zhang, X.; MacMillan, D. W. C. *J. Am. Chem. Soc.* **2017**, *139,* 11353.

Table S3. Evaluation of additives

Entry	HAT catalyst	Yield (%)	decarbonylated product 4 (%)	remaining Aryl-Br 1a (%)
1	**NaBr (20 mol%)**	33	4	10
2	**TBAB (20 mol%)**	10	-	60
3	**LiCl (20 mol%)**	24	10	5
4	**NaBr (50 mol%)**	44	15	5
5	**no additive**	30	18	8

Reaction conditions: **2a** (0.22 mmol), **1a** (0.11 mmol), **PC** (1 mol%), $NiBr_2$.DME (10 mol%), dtbbpy (10 mol%), K_2CO_3 (1.5 equiv.), and **additive** in dioxane (3 mL) under 10W blue LED irradiation for 24 h. Yields were determined by ^1H NMR using 1,3-benzodioxole as internal standard.

Table S4. Evaluation of aldehyde equivalents

Entry	Aldehyde equivalents	Yield (%)	decarbonylated product 4 (%)	remaining Aryl-Br 1a (%)
1	**2 equivalents**	30	20	10
2	**3 equivalents**	40	14	6
3	**4 equivalents**	42	18	5
4	**1 equiv. (as limiting reagent)[a]**	34	10	25

Reaction conditions: **1a** (0.11 mmol), **PC** (1 mol%), $NiBr_2$.DME (10 mol%), dtbbpy (10 mol%), K_2CO_3 (1.5 equiv.) in dioxane (3 mL) under 10W blue LED irradiation for 24 h. Yields were determined by ^1H NMR using 1,3-benzodioxole as internal standard. [a]aldehyde (0.11 mmol), **1a** (0.22 mmol).

Table S5. Control tests

Entry	Deviations	Yield (%)	decarbonylated product 4 (%)
1	**no Ir photocatalyst**	-	-
2	**in the dark**	-	-
3	**no Ni catalyst**	-	-
4	**no HAT catalyst**	32	20

Reaction conditions: **2a** (0.22 mmol), **1a** (0.11 mmol), **PC** (1 mol%), $NiBr_2$.DME (10 mol%), dtbbpy (10 mol%), K_2CO_3 (1.5 equiv.) in dioxane (3 mL) under 10W blue LED irradiation for 24 h. Yields were determined by ^1H NMR using 1,3-benzodioxole as internal standard.

5. Synthesis of α-amino aryl ketones from *N*-Boc-L-alanine

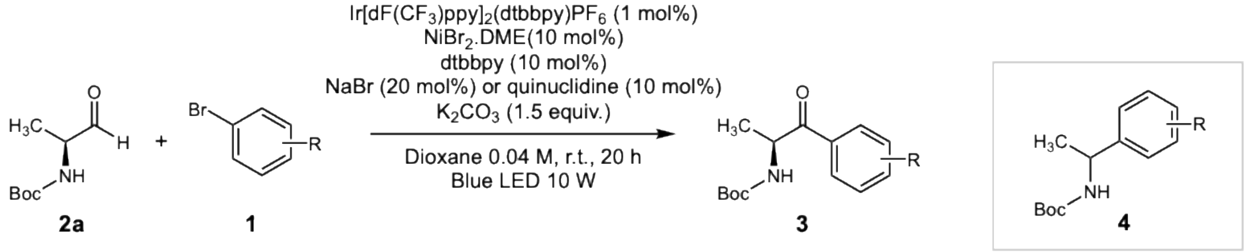

To a 2-dram clear vial were added $NiBr_2$·DME (4 mg, 0.01 mmol, 0.1 equiv.), 4,4′-Di-tert-butyl-2,2′-dipyridyl (dtbbpy, 3 mg, 0.01 mmol, 0.1 equiv.), and dioxane (3 mL). The mixture was sonicated for 15 minutes until a clear yellowish solution was obtained. In another 2-dram clear vial equipped with a magnetic stirring bar, the photocatalyst (Ir[dF(CF_3)ppy]$_2$(dtbbpy))PF_6, 1.2 mg, 0.0011 mmol, 1 mol%), N-Boc-L-alanine (38 mg, 0.22 mmol, 2 equiv.), K_2CO_3 (22 mg, 0.165 mmol, 1.5 equiv.), aryl bromide if solid (0.11 mmol, 1 equiv.), and NaBr (2,3 mg, 0.02 mmol, 0.2 equiv.) or quinuclidine (1,2 mg, 0.01 mmol, 0,1 equiv.) were added. The nickel catalyst solution was then transferred to the vial containing the other reagents. This vial was sealed with a rubber septum and sparged with argon for 15 minutes. If the aryl bromide is volatile, it should be added only after the solution has been sparged. The reaction was sealed with parafilm and irradiated with one 10W blue LED (at 1 cm distance from the light source) under stirring for 20 hours at room temperature. After that time, 13 µL of 1,3-benzodioxole (0.11 mmol) was added to the reaction vial as an internal standard. The solution was stirred for 2 minutes, filtered through Celite, and analyzed by ^{1}H NMR. The product was isolated by flash column chromatography to afford the ketone or ketone/decarbonylated mixture.

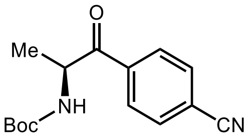

***tert*-butyl (*S*)-(1-(4-cyanophenyl)-1-oxopropan-2-yl)carbamate (3a)**

Prepared according to the General Procedure described in sub-section 5, using N-Boc-L-alanine (57 mg, 0.33 mmol, 3 equiv.), 4-bromobenzonitrile (20 mg, 0.11 mmol, 1 equiv.), and NaBr (20 mol%). The crude mixture was purified by flash column chromatography using a gradient elution (0-30% ethyl acetate in hexanes), affording a mixture of α-amino aryl ketone **3a** and decarbonylated product **4a** in 50% yield (4:1) as a white solid.

^{1}H NMR of α-amino aryl ketone (3a) (500 MHz, $CDCl_3$) δ 8.07 (d, *J* = 8.2 Hz, 2H), 7.79 (d, *J* = 8.2 Hz, 2H), 5.38 (d, *J* = 7.4 Hz, 1H), 5.29 – 5.21 (m, 1H), 1.46 (s, 9H), 1.39 (d, *J* = 7.1 Hz, 3H).
^{1}H NMR of decarbonylated product (4a) (500 MHz, $CDCl_3$) δ 7.62 (d, *J* = 8.2 Hz, 2H), 7.40 (d, *J* = 8.2 Hz, 2H), 5.08 (br s, 1H), 4.81 (br s, 1H), 1.45 (s, 9H), 1.34 (d, *J* = 7.4 Hz, 3H).

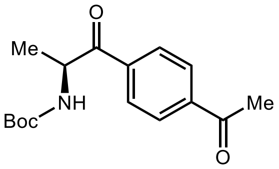

***tert*-butyl (*S*)-(1-(4-acetylphenyl)-1-oxopropan-2-yl)carbamate (3b)**

Prepared according to the General Procedure described in sub-section 5, using 4-bromoacetophenone (21.9 mg, 0.11 mmol, 1 equiv.) and NaBr (20 mol%). The crude mixture was purified by flash column chromatography using a gradient elution (0-20% ethyl acetate in hexanes), affording the α-amino aryl ketone **3b** in 48% yield (15 mg) as a white solid.

^{1}H NMR (400 MHz, $CDCl_3$) δ 8.04 (s, 4H), 5.49 (d, *J* = 6.8 Hz, 1H), 5.34 – 5.22 (m, 1H), 2.64 (s, 3H), 1.44 (s, 9H), 1.39 (d, *J* = 7.2 Hz, 3H).

^{13}C NMR (101 MHz, $CDCl_3$) δ 199.3, 197.5, 155.3, 140.7, 137.8, 129.0, 128.8, 80.1, 51.6, 28.5, 27.1, 19.6.

HRMS (ESI) *m/z:* $[M+H]^+$ calcd. for $C_{16}H_{22}NO_4$ 292.1543; found 292.1544.

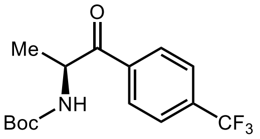

***tert*-butyl (*S*)-(1-oxo-1-(4-(trifluoromethyl)phenyl)propan-2-yl)carbamate (3c)**

Prepared according to the General Procedure described in sub-section 5, using 4-bromobenzotrifluoride (25 mg, 16 μL, 0.11 mmol, 1 equiv.) and NaBr (20 mol%). The crude mixture was purified by flash column chromatography using a gradient elution (0-30% ethyl acetate in hexanes), affording the α-amino aryl ketone **3c** in 60% yield (21 mg) as a white solid.

^{1}H NMR (500 MHz, $CDCl_3$) δ 8.08 (d, *J* = 8.1 Hz, 2H), 7.75 (d, *J* = 8.2 Hz, 2H), 5.46 (d, *J* = 6.7 Hz, 1H), 5.35 – 5.20 (m, 1H), 1.45 (s, 9H), 1.39 (d, *J* = 7.1 Hz, 3H).

^{13}C NMR (126 MHz, $CDCl_3$) δ 198.9, 155.3, 137.3, 135.0 (q, *J* = 32.8 Hz), 129.1, 126.0 (q, *J* = 3.7 Hz), 123.6 (q, *J* = 272.8 Hz), 80.2, 51.5, 28.5, 19.4.

HRMS (ESI) *m/z*: $[M+K]^+$calcd. for $C_{15}H_{18}F_3NO_3$ 356,0870; found 356.0867.

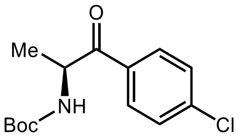

***tert*-butyl (*S*)-(1-(4-chlorophenyl)-1-oxopropan-2-yl)carbamate (3d)**

Prepared according to the General Procedure described in sub-section 5, using 1-bromo-4-chlorobenzene (21 mg, 0.11 mmol, 1 equiv.) and NaBr (20 mol%). The crude mixture was purified by flash column chromatography using a gradient elution (0-30% ethyl acetate in hexanes), affording the α-amino aryl ketone **3c** in 44% yield (13 mg) as a white solid.

^{1}H NMR (500 MHz, $CDCl_3$) δ 7.92 (d, *J* = 8.5 Hz, 2H), 7.46 (d, *J* = 8.6 Hz, 2H), 5.48 (d, *J* = 6.9 Hz, 1H), 5.30 – 5.16 (m, 1H), 1.45 (s, 9H), 1.38 (d, *J* = 7.1 Hz, 3H).

^{13}C NMR (126 MHz, $CDCl_3$) δ 198.5, 155.3, 140.4, 132.7, 130.2, 129.3, 80.0, 51.1, 28.5, 19.8.

HRMS (ESI) *m/z*: $[M+Na]^+$ calcd. for $C_{14}H_{18}ClNO_3$ 306.0867; found 306.0873.

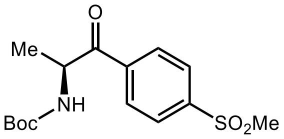

***tert*-butyl (*S*)-(1-(4-(methylsulfonyl)phenyl)-1-oxopropan-2-yl)carbamate (3e)**

Prepared according to the General Procedure described in sub-section 5, using 4-bromophenyl methyl sulfone (25.8 mg, 0.11 mmol, 1 equiv.) and NaBr (20 mol%). The crude mixture was purified by flash column chromatography using a gradient elution (0-30% ethyl acetate in hexanes), affording a mixture of α-amino aryl ketone **3e** and decarbonylated product **4e** in 73% yield (3:1) as a white solid.

^{1}H NMR of α-amino aryl ketone (3e) (500 MHz, $CDCl_3$) δ 8.15 (d, *J* = 8.4 Hz, 2H), 8.06 (d, *J* = 8.5 Hz, 2H), 5.41 (d, *J* = 7.2 Hz, 1H), 5.32 – 5.22 (m, 1H), 3.08 (s, 3H), 1.44 (s, 9H), 1.39 (d, *J* = 7.1 Hz, 3H).

^{1}H NMR of decarbonylated product (4e) (500 MHz, $CDCl_3$) δ 7.89 (d, *J* = 8.4 Hz, 2H), 7.49 (d, *J* = 8.3 Hz, 2H), 4.91 (br s, 1H), 4.82 (br s, 1H), 3.03 (s, 3H), 1.44 (s, 9H), 1.39 (d, *J* = 7.1 Hz, 3H).

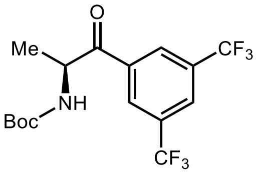

***tert*-butyl (*S*)-(1-(3,5-bis(trifluoromethyl)phenyl)-1-oxopropan-2-yl)carbamate (3f)**

Prepared according to the General Procedure described in sub-section 5, using 1-bromo-3,5-bis(trifluoromethyl)benzene (32 mg, 19 μL, 0.11 mmol, 1 equiv.) and NaBr (20 mol%). The crude mixture was purified by flash column chromatography using a gradient elution (0-30% ethyl acetate in hexanes), affording a mixture of α-amino aryl ketone **3f** and decarbonylated product **4f** in 43% yield (1:1.4) as a yellowish solid.

^{1}H NMR of α-amino aryl ketone (3f) (400 MHz, $CDCl_3$) δ 8.43 (s, 2H), 7.76 (s, 1H), 5.33 (br s, 1H), 4.90 (br s, 1H), 1.48 (s, *J* = 6.8 Hz, 3H), 1.45 (s, 9H).
^{1}H NMR of decarbonylated product (4f) (400 MHz, $CDCl_3$) δ 8.09 (s, 1H), 7.75 (s, 2H), 5.30 – 5.27 (m, 1H), 4.90 (br s, 1H), 1.45 (s, 9H), 1.44 (d, *J* = 6.3 Hz, 3H).

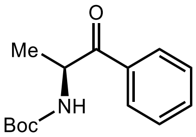

tert-butyl (*S*)-(1-oxo-1-phenylpropan-2-yl)carbamate (3g)

Prepared according to the General Procedure described in sub-section 5, using 4-bromobenzeno (17 mg, 11 μL, 0.11 mmol, 1 equiv.) and quinuclidine (10 mol%). The crude mixture was purified by flash column chromatography using a gradient elution (0-20% ethyl acetate in hexanes), affording the α-amino aryl ketone **3g** in 15% yield (4 mg) as a colorless oil. When performed at same conditions, using N-Boc-L-alanine (57 mg, 0.33 mmol, 3 equiv.), the α-amino aryl ketone **3g** was obtained in 36% yield (10 mg) as a colorless oil.

^{1}H NMR (500 MHz, $CDCl_3$) δ 7.97 (d, *J* = 7.4 Hz, 2H), 7.62 – 7.57 (m, *J* = 1H), 7.49 (t, *J* = 7.7 Hz, 2H), 5.56 (d, *J* = 7.2 Hz, 1H), 5.33 – 5.25 (m, 1H), 1.46 (s, 9H), 1.40 (d, *J* = 7.1 Hz, 3H).

^{13}C NMR (126 MHz, $CDCl_3$) δ 199.62, 155.33, 134.37, 133.85, 128.97, 128.80, 79.86, 51.25, 28.53, 20.07.

HRMS (ESI) *m/z*: [M+Na]$^+$ calcd. for $C_{14}H_{19}NO_3$ 272.1257; found 272.1266.

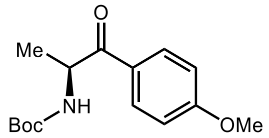

***tert*-butyl (*S*)-(1-(4-methoxyphenyl)-1-oxopropan-2-yl)carbamate (3h)**

Prepared according to the General Procedure described in sub-section 5, using *N*-Boc-L-alanine (57 mg, 0.33 mmol, 3 equiv.), 4-bromoanisole (21 mg, 13 µL, 0.11 mmol, 1 equiv.) and quinuclidine (10 mol%). The crude mixture was purified by flash column chromatography using a gradient elution (0-30% ethyl acetate in hexanes), affording the α-amino aryl ketone **3h** in 20% yield (6 mg) as a colorless oil.

^{1}H NMR (400 MHz, $CDCl_3$) δ 7.96 (d, *J* = 8.8 Hz, 2H), 6.96 (d, *J* = 8.9 Hz, 2H), 5.59 (d, *J* = 6.6 Hz, 1H), 5.28 – 5.23 (m, 1H), 3.88 (s, 3H), 1.45 (s, 9H), 1.39 (d, *J* = 7.0 Hz, 3H).

^{13}C NMR (101 MHz, $CDCl_3$) δ 198.0, 164.1, 155.3, 131.2, 127.2, 114.2, 79.7, 55.7, 50.8, 28.5, 20.4.

HRMS (ESI) *m/z*: $[M+H]^+$ calcd. for $C_{15}H_{21}NO_4$ 280.1543; found 280.1558.

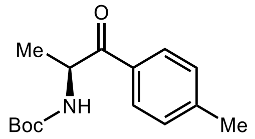

tert-butyl (*S*)-(1-oxo-1-(p-tolyl)propan-2-yl)carbamate (3i)

Prepared according to the General Procedure described in sub-section 5, using 1-bromo-4-methylbenzene (19 mg, 0.11 mmol, 1 equiv.) and quinuclidine (10 mol%). The yield was determined by ^{1}H quantitative NMR using 1,3-benzodioxole as the internal standard. Isolation of the product was not possible due to its very low yield.

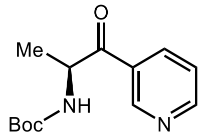

tert-butyl (*S*)-(1-oxo-1-(pyridin-3-yl)propan-2-yl)carbamate (3j)

Prepared according to the General Procedure described in sub-section 5, using 3-bromopyridine (17 mg, 11 µL, 0.11 mmol, 1 equiv.) and NaBr (20 mol%). The crude mixture was purified by flash column chromatography using a gradient elution (20-60% ethyl acetate in hexanes), affording the α-amino aryl ketone **3j** in 28% yield (8 mg) as a colorless oil.

^{1}H NMR (500 MHz, CDCl$_3$) δ 9.18 (d, *J* = 1.8 Hz, 1H), 8.80 (dd, *J* = 4.8, 1.5 Hz, 1H), 8.25 (dt, *J* = 8.0, 1.9 Hz, 1H), 7.44 (dd, *J* = 7.9, 4.9 Hz, 1H), 5.46 (d, *J* = 6.9 Hz, 1H), 5.29 – 5.19 (m, 1H), 1.44 (s, 9H), 1.41 (d, *J* = 7.1 Hz, 3H).

^{13}C NMR (101 MHz, CDCl$_3$) δ 198.7, 155.3, 154.1, 150.1, 136.1, 130.0, 123.9, 80.2, 51.5, 28.5, 19.4.

HRMS (ESI) *m/z*: [M+Li]$^+$ calcd. for $C_{13}H_{18}N_2O_3$ 257.1472; found 257.1472.

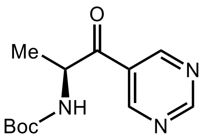

tert-butyl (*S*)-(1-oxo-1-(pyrimidin-5-yl)propan-2-yl)carbamate (3k)

Prepared according to the General Procedure described in sub-section 5,5-bromopyrimidine (18 mg, 0.11 mmol, 1 equiv.) and NaBr (20 mol%). The crude mixture was purified by flash column chromatography using a gradient elution (20-60% ethyl acetate in hexanes), affording the α-amino aryl ketone **3k** in 25% yield (7 mg) as a yellowish oil.

^{1}H NMR (500 MHz, CDCl$_3$) δ 9.39 (s, 1H), 9.27 (s, 2H), 5.33 (d, *J* = 6.6 Hz, 1H), 5.21 – 5.11 (m, 1H), 1.48 (d, *J* = 6.1 Hz, 3H), 1.44 (s, 9H).

^{13}C NMR (101 MHz, CDCl$_3$) δ 197.5, 161.8, 158.5, 157.1, 155.3, 127.9, 80.6, 51.8, 28.4, 18.6.

HRMS (ESI) *m/z*: [M+H]$^+$ calcd. for $C_{12}H_{18}N_3O_3$ 252.1343; found 252.1357.

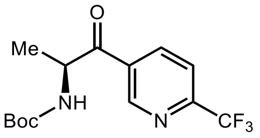

tert-butyl (*S*)-(1-oxo-1-(6-(trifluoromethyl)pyridin-3-yl)propan-2-yl)carbamate (3l)

Prepared according to the General Procedure described in sub-section 5, using 5-bromo-2-(trifluoromethyl)pyridine (25 mg, 0.11 mmol, 1 equiv.) and NaBr (20 mol%). The crude mixture was purified by flash column chromatography using a gradient elution (20-60% ethyl acetate in hexanes), affording a mixture of α-amino aryl ketone **3l** and decarbonylated product **4l** in 35% yield (1:3) as a colorless oil.

^{1}H NMR of α-amino aryl ketone (3l) (400 MHz, CDCl$_3$) δ 9.28 (s, 1H), 8.46 (d, *J* = 8.0 Hz, 1H), 7.84 (d, *J* = 8.2 Hz, 1H), 5.39 – 5.32 (m, 1H), 5.28 – 5.20 (m, 1H), 1.51 (d, *J* = 6.1 Hz, 9H), 1.40 (m, 3H)

^{1}H NMR of decarbonylated product (4l) (400 MHz, CDCl$_3$) δ 8.70 (s, 1H), 7.81 (d, *J* = 7.9 Hz, 1H), 7.66 (d, *J* = 8.1 Hz, 1H), 5.08 (br s, 1H), 4.88 (br s, 2H), 1.45 (d, *J* = 3.3 Hz, 9H), 1.43 (m, 3H).

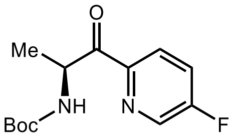

tert-butyl (*S*)-(1-(5-fluoropyridin-2-yl)-1-oxopropan-2-yl)carbamate (3m)

Prepared according to the General Procedure described in sub-section 5, using 2-bromo-5-fluoropyridine (19.3 mg, 0.11 mmol, 1 equiv.) and NaBr (20 mol%). The crude mixture was purified by flash column chromatography using a gradient elution (20-60% ethyl acetate in hexanes), affording a mixture of α-amino aryl ketone **3a** and decarbonylated product **4a** in 42% yield (1.6:1) as a white solid.

^{1}H NMR of α-amino aryl ketone (3m) (500 MHz, $CDCl_3$) δ 8.53 (d, *J* = 2.8 Hz, 1H), 8.14 (dd, *J* = 8.7, 4.6 Hz, 1H), 7.53 (td, *J* = 8.4, 2.6 Hz, 1H), 5.70 – 5.65 (m, 1H), 5.40 (br s, 1H), 1.46 – 1.42 (m, 12H).

^{1}H NMR of decarbonylated product (4m) (500 MHz, $CDCl_3$) δ 8.39 (d, *J* = 2.8 Hz, 1H), 7.36 (td, *J* = 8.4, 2.8 Hz, 1H), 7.31 (dd, *J* = 5.2, 1.8 Hz, 1H), 5.52 (br s, 1H), 4.86 (br s, 1H), 1.44 – 1.43 (m, 3H), 1.39 (s, 9H).

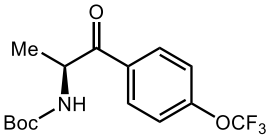

tert-butyl (*S*)-(1-oxo-1-(4-(trifluoromethoxy)phenyl)propan-2-yl)carbamate (3n)

Prepared according to the General Procedure described in sub-section 5, 1-Bromo-4-(trifluoromethoxy)benzene (27 mg, 16 μL, 0.11 mmol, 1 equiv.) and NaBr (20 mol%). The crude mixture was purified by flash column chromatography using a gradient elution (0-20% ethyl acetate in hexanes), affording the α-amino aryl ketone **3n** in 25% yield (9 mg) as a yellowish oil.

^{1}H NMR (400 MHz, $CDCl_3$) δ 8.04 (d, *J* = 8.8 Hz, 2H), 7.31 (d, *J* = 8.3 Hz, 2H), 5.47 (d, *J* = 7.3 Hz, 1H), 5.31 – 5.20 (m, 1H), 1.45 (s, 9H), 1.40 (d, *J* = 7.2 Hz, 3H).

^{13}C NMR (126 MHz, $CDCl_3$) δ 198.2, 155.3, 153.2, 149.2, 132.7, 130.9, 120.7, 120.4 (q, *J* = 259.1 Hz), 80.1, 51.2, 30.8, 28.5, 19.7.

HRMS (ESI) *m/z*: [M+Na]$^+$calcd. for $C_{15}H_{18}F_3NO_4$ 356.1080; found 356.1084.

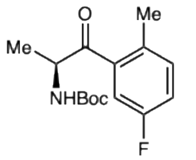

tert-butyl (*S*)-(1-(3,5-bis(trifluoromethyl)phenyl)-1-oxopropan-2-yl)carbamate (3o)

Prepared according to the General Procedure described in sub-section 5, using di-tert-butyl (6-oxohexane-1,5-diyl)(S)-dicarbamate (73 mg, 0.22 mmol, 2 equiv.) and 2-bromo-4-fluoro-1-methylbenzene (21 mg, 14 µL, 0.11 mmol, 1 equiv.). Product formation was not observed.

6. Synthesis of α-amino aryl ketones from other α-amino aldehydes

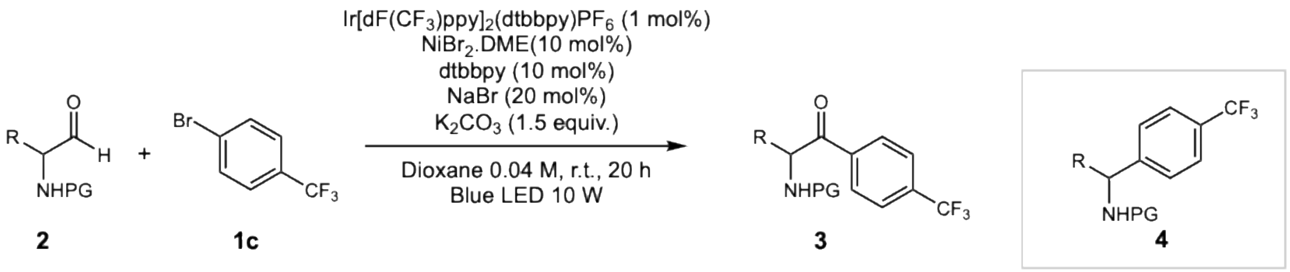

To a 2-dram clear vial were added $NiBr_2$·DME (4 mg, 0.01 mmol, 0.1 equiv.), 4,4′-di-tert-butyl-2,2′-dipyridyl (dtbbpy, 3 mg, 0.01 mmol, 0.1 equiv.), and dioxane (3 mL). The mixture was sonicated for 15 minutes until a clear yellowish solution was obtained. In another 2-dram clear vial equipped with a magnetic stirring bar, the photocatalyst (Ir[dF(CF_3)ppy]$_2$(dtbbpy))PF_6, 1.2 mg, 0.0011 mmol, 1 mol%), α-amino aldehyde (0.11-0.22 mmol, 1-2 equiv.), K_2CO_3 (22 mg, 0.165 mmol, 1.5 equiv.), and NaBr (2.3 mg, 0.02 mmol, 0.2 equiv.) were added. The nickel catalyst solution was then transferred to the vial containing the other reagents. This vial was sealed with a rubber septum and sparged with argon for 15 minutes. After sparging, 4-bromobenzotrifluoride (25 mg, 16 µL, 0.11 mmol, 1 equiv.) was added and the reaction vial was sealed with parafilm and irradiated with one 10W blue LED (at 1 cm distance from the light source) under stirring for 20 hours at room temperature. Then, 13 µL of 1,3-benzodioxole (0.11 mmol) was added to the reaction vial as an internal standard. The solution was stirred for 2 minutes, filtered through Celite, and analyzed by ^{1}H NMR. The product was isolated by flash column chromatography to afford the ketone or ketone/decarbonylated mixture.

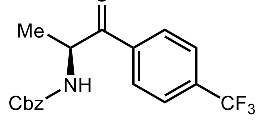

benzyl (1-oxo-1-(4-(trifluoromethyl)phenyl)propan-2-yl)carbamate (3p)

Prepared according to the General Procedure described in sub-section 6, using benzyl (1-oxopropan-2-yl)carbamate (46 mg, 0.22 mmol, 2 equiv.) and 4-bromobenzotrifluoride (25 mg, 16 µL, 0.11 mmol, 1 equiv.).

The crude mixture was purified by flash column chromatography using a gradient elution (0-40% ethyl acetate in hexanes), affording the α-amino aryl ketone **3p** in 72% yield (4:1) as a white solid.

^{1}H NMR (500 MHz, $CDCl_3$) of mixture of compounds 3p + 4p δ 8.09 (d, *J* = 8.3 Hz, 2H), 7.77 (d, *J* = 8.1 Hz, 2H), 7.38 – 7.30 (m, 5H), 5.76 (d, *J* = 7.1 Hz, 1H), 5.40 - 5.31 (m, 1H), 5.13 (s, 2H), 1.44 (d, *J* = 7.1 Hz, 3H).

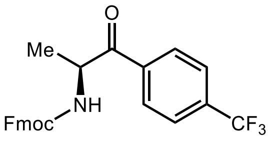

(9H-fluoren-9-yl)methyl (1-oxo-1-(4-(trifluoromethyl)phenyl)propan-2-yl)carbamate (3q)

Prepared according to the General Procedure described in sub-section 6, using (9H-fluoren-9-yl)methyl (*S*)-(1-oxopropan-2-yl)carbamate (65 mg, 0.22 mmol, 2 equiv.) and 4-bromobenzotrifluoride (25 mg, 16 µL, 0.11 mmol, 1 equiv.). Product formation was not observed.

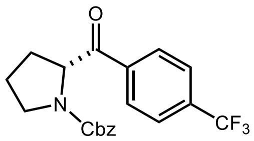

benzyl (*R*)-2-(4-(trifluoromethyl)benzoyl)pyrrolidine-1-carboxylate (3r)

Prepared according to the General Procedure described in sub-section 6, using benzyl (*R*)-2-formylpyrrolidine-1-carboxylate (51 mg, 0.22 mmol, 2 equiv.) and 4-bromobenzotrifluoride (25 mg, 16 µL, 0.11 mmol, 1 equiv.). The crude mixture was purified by flash column chromatography using a gradient elution (0-40% ethyl acetate in hexanes), affording the α-amino aryl ketone **3r** in 45% yield (1:1) as a white solid.

^{1}H NMR of mixture of compounds 3r+ 4r (500 MHz, $CDCl_3$) δ 8.10 (d, *J* = 8.1 Hz, 2H), 7.74 (d, *J* = 8.3 Hz, 2H), 7.40 – 7.29 (m, 5H), 5.36 (dd, *J* = 9.1, 3.5 Hz, 1H), 5.21 – 5.08 (m, 2H), 3.81 – 3.52 (m, 2H), 2.06 – 1.89 (m, 4H).

HRMS (ESI) *m/z*: [M+Na]$^+$ calcd. for $C_{20}H_{18}F_3NaNO_3$ 400.1131; found 400.1130.

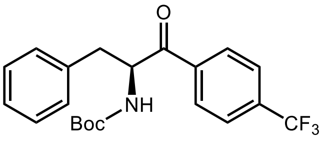

tert-butyl (*S*)-(1-oxo-3-phenyl-1-(4-(trifluoromethyl)phenyl)propan-2-yl)carbamate (3s)

Prepared according to the General Procedure described in sub-section 5, using *tert*-butyl-(*S*)-(1-oxo-3-phenylpropan-2-yl)carbamate (56 mg, 0.22 mmol, 2 equiv.) and 4-bromobenzotrifluoride (25 mg, 16 µL, 0.11 mmol, 1 equiv.). The crude mixture was purified by flash column chromatography using a gradient elution (0-30% ethyl acetate in hexanes), affording the α-amino aryl ketone **3s** in 30% yield (13 mg) as a white solid.

^{1}H NMR (400 MHz, CDCl$_3$) δ 8.01 (d, *J* = 8.1 Hz, 2H), 7.72 (d, *J* = 8.3 Hz, 2H), 7.21 (m, 3H), 7.02 (d, *J* = 6.5 Hz, 2H), 5.55 – 5.47 (m, 1H), 5.33 (d, *J* = 8.0 Hz, 1H), 3.20 (dd, *J* = 13.9, 6.3 Hz, 1H), 2.99 (dd, *J* = 13.7, 5.9 Hz, 1H), 1.42 (s, 9H).

^{13}C NMR (126 MHz, CDCl$_3$) δ 198.2, 155.3, 138.1, 134.9 (q, *J* = 32.7 Hz), 129.6, 129.1, 128.6, 127.2, 125.9, (d, J = 3.5 Hz), 123.60 (d, *J* = 272.8 Hz), 80.3, 56.5, 39.0, 28.5.

HRMS (ESI) *m/z*: [M+H]$^+$ calcd. for $C_{21}H_{22}F_3NO_3$ 394.1552; found 394.1560.

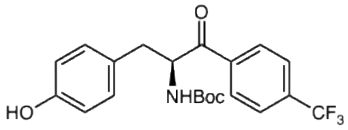

***tert*-butyl (*S*)-(3-(4-hydroxyphenyl)-1-oxo-1-(4-(trifluoromethyl)phenyl)propan-2-yl)carbamate (3t)**

Prepared according to the General Procedure described in sub-section 5, using *tert*-butyl (*S*)-(1-(4-hydroxyphenyl)-3-oxopropan-2-yl)carbamate (90 mg, 0.22 mmol, 2 equiv.) and 4-bromobenzotrifluoride (25 mg, 16 µL, 0.11 mmol, 1 equiv.). The yield was determined by ^{1}H quantitative NMR using 1,3-benzodioxole as the internal standard, revealing a mixture of **3t** and **4t** in 48% yield (1:1). Isolation of the pure products was not possible due to the formation of a complex mixture.

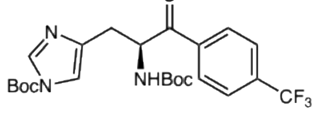

tert-butyl (*S*)-4-(2-((tert-butoxycarbonyl)amino)-3-oxo-3-(4-(trifluoromethyl)phenyl)propyl)-1H-imidazole-1-carboxylate (3u)

Prepared according to the General Procedure described in sub-section 5, using *tert*-butyl (*S*)-4-(2-((tert-butoxycarbonyl)amino)-3-oxopropyl)-1H-imidazole-1-carboxylate (**2u**) (106 mg, 0.22 mmol, 2 equiv.) and 4-bromobenzotrifluoride (25 mg, 16 µL, 0.11 mmol, 1 equiv.). The yield was determined by ^{1}H quantitative NMR using 1,3-benzodioxole as the internal standard, revealing a mixture of **3u** and **4u** in 47% yield (1:1). Isolation of the pure products was not possible due to the formation of a complex mixture.

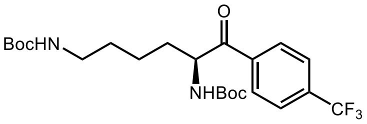

di-tert-butyl (6-oxo-6-(4-(trifluoromethyl)phenyl)hexane-1,5-diyl)(S)-dicarbamate (3v)

Prepared according to the General Procedure described in sub-section 5, using di-tert-butyl (6-oxohexane-1,5-diyl)(S)-dicarbamate (73 mg, 0.22 mmol, 2 equiv.) and 4-bromobenzotrifluoride (25 mg, 16 µL, 0.11 mmol, 1 equiv.). Product formation was not observed.

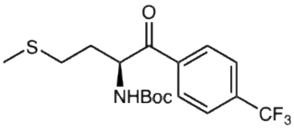

***tert*-butyl (*S*)-(4-(methylthio)-1-oxo-1-(4-(trifluoromethyl)phenyl)butan-2-yl)carbamate (3x)**

Prepared according to the General Procedure described in sub-section 5, using *tert*-butyl (*S*)-(4-(methylthio)-1-oxobutan-2-yl)carbamate (**2x**) (83 mg, 0.22 mmol, 2 equiv.) and 4-bromobenzotrifluoride (25 mg, 16 µL, 0.11 mmol, 1 equiv.). Product formation was not observed.

7. Synthesis of cathinones derivatives

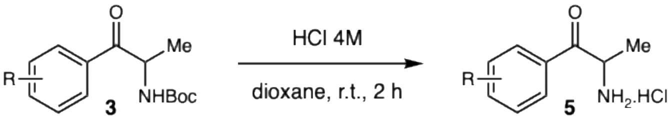

In a 4-dram vial containing the α-amino aryl ketone, a solution of 4 M HCl in dioxane was added a 0°C. After stirring for 2 h at room temperature, the mixture was concentrated in vacuum, and the hydrochloride salt was obtained in quantitative yield.

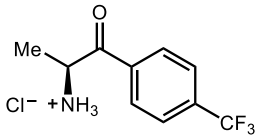

(*S*)-2-amino-1-(4-(trifluoromethyl)phenyl)propan-1-one hydrochloride (5c)

Prepared using *tert*-butyl (*S*)-(1-oxo-1-(4-(trifluoromethyl)phenyl)propan-2-yl)carbamate (**3c)** (16 mg, 0.050 mmol, 1 equiv.) and 600 µL of 4 M HCl solution in dioxane. Product was obtained without further purification as a white solid (12 mg, quantitative yield).

1H NMR (500 MHz, D2O) δ 8.24 (d, J = 8.1 Hz, 2H), 7.92 (d, J = 8.0 Hz, 2H), 5.19 (d, J = 7.3 Hz, 1H), 1.58 (d, J = 7.2 Hz, 3H).

^{13}C NMR (126 MHz, CD_3OD): δ 196.7, 137.3, 136.4 (q, J = 32.7 Hz), 130.7, 127.4 – 127.20 (m), 125.0 (q, J = 272.1 Hz), 53.2, 17.4.

HRMS (ESI) *m/z*: [M+H]$^+$ calcd. for $C_{10}H_{10}F_3NO$ 218.0787; found 218.0777.

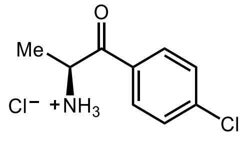

(*S*)-2-amino-1-(4-chlorophenyl)propan-1-one hydrochloride (5d)

Prepared using *tert*-butyl (*S*)-(1-(4-chlorophenyl)-1-oxopropan-2-yl)carbamate (**3d)** (17 mg, 0.058 mmol, 1 equiv.) and 660 µL of 4 M HCl solution in dioxane. Product was obtained without further purification as a white solid (13 mg, quantitative yield)

^{1}H NMR (400 MHz, D2O) δ 8.04 (d, J = 7.4 Hz, 2H), 7.59 (d, J = 7.0 Hz, 2H), 5.14 (br s, 1H), 1.58 (d, J = 5.2 Hz, 3H).

^{13}C NMR (101 MHz, CD_3OD) δ 196.2, 142.0, 132.7, 131.7, 130.6, 53.0, 17.7.

HRMS (ESI) *m/z*: [M+H]$^+$ calcd. for $C_9H_{10}ClNO$ 184.0524; found 184.0511.

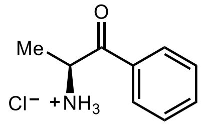

(*S*)-2-amino-1-phenylpropan-1-one hydrochloride (5g)

Prepared using *tert*-butyl (*S*)-(1-oxo-1-phenylpropan-2-yl)carbamate (**3g)** (12.9 mg, 0.052 mmol, 1 equiv.) and 600 µL of 4 M HCl solution in dioxane. Product was obtained without further purification as a slightly beige solid (8,7 mg, 90% yield).

^{1}H NMR (400 MHz, D2O) δ 7.98 – 7.93 (m, 2H), 7.62 (td, J = 7.2, 1.2 Hz, 1H), 7.49 (t, J = 7.7 Hz, 2H), 5.08 – 5.0 (m, 1H), 1.47 (d, J = 7.1 Hz, 3H).

^{13}C NMR (101 MHz, CD_3OD) δ 197.3, 135.8, 134.2, 130.3, 129.9, 52.9, 17.8.

HRMS (ESI) *m/z*: [M+H]$^+$ calcd. for $C_9H_{11}NO$ 150.0913; found 150.0919.

8. Chiral HPLC analysis

To verify the degree of racemization during reaction conditions, the product **3p,** from N-Cbz-L-alanine aminoaldehyde, was selected as a representative example and submitted to chiral HPLC analysis. The analysis was carried out on a Shimadzu Prominence LC-20A equipped with a PDA detector, using a Lux 5mm Cellulose-2 LC Column 250 x 4.6 mm. Mobile phases comprised HPLC grade hexanes and HPLC grade isopropanol. The method for chromatographic separation consisted of an isocratic run using 10% Isopropanol in hexanes for 30 minutes. The flow rate was set to 1 mL.min^{-1} and the injection volume was 2 µL. Column oven temperature was kept at 35 °C throughout the analysis. The substances were detected spectrophotometrically at =254 nm.

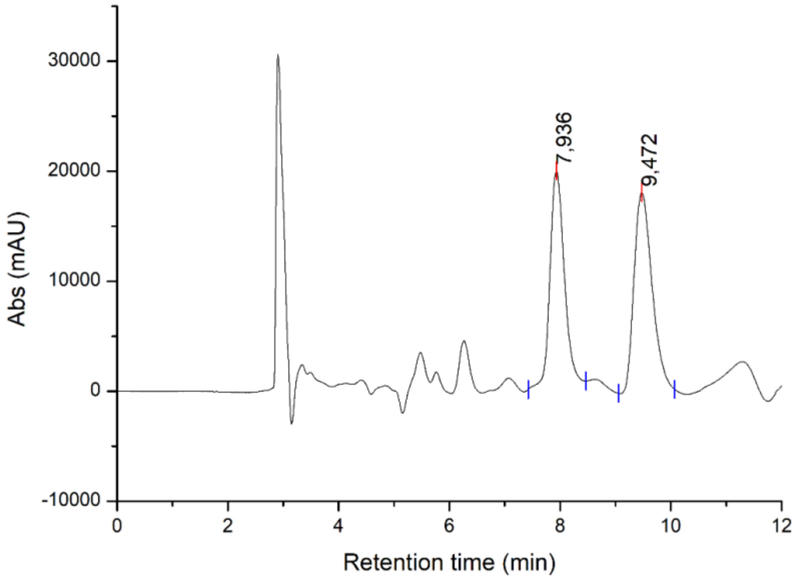

Peak	Ret. Time	Area	Area (%)
1	7.936 min	1684.617	48.22
2	9.472 min	1808.968	51.78

Figure S1: HPLC chromatogram of **rac** (±)-**2p**

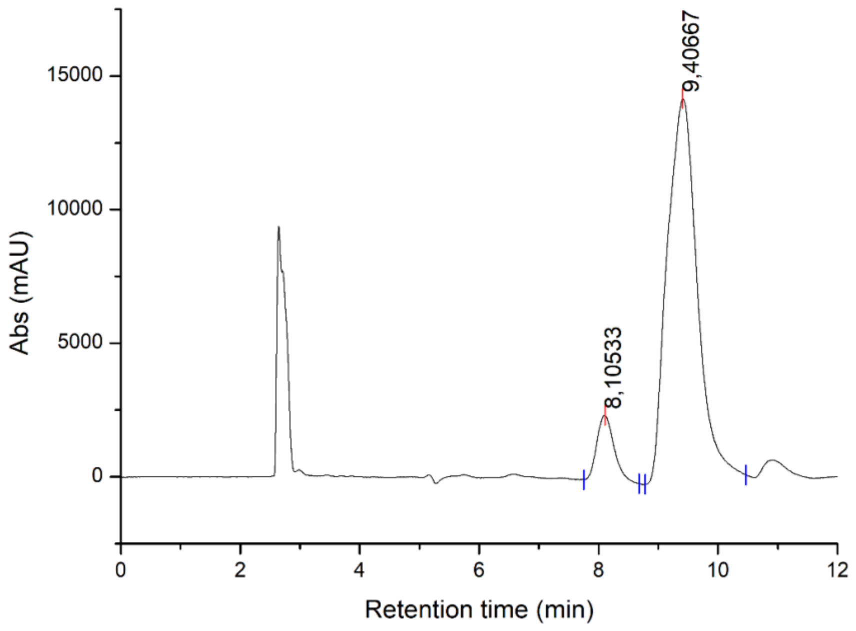

Peak	Ret. Time	Area	Area (%)
1	8.105 min	597.716	7.54
2	9.407 min	7328.662	92.46

Figure S2: HPLC chromatogram of (*S*)-**2p**

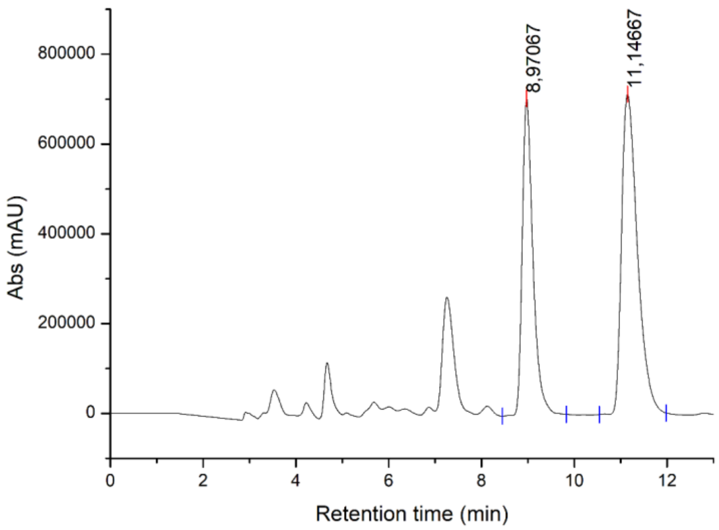

Peak	Ret. Time	Area	Area (%)
1	8.97 min	2040.448	39.7
2	11.15 min	3100.144	60.3

Figure S3: HPLC chromatogram of **rac** (±)-**3p**

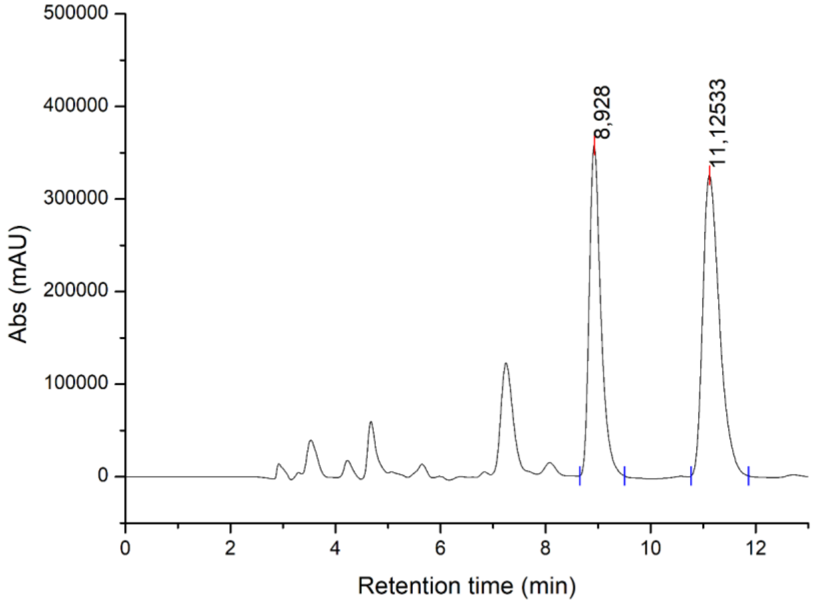

Peak	Ret. Time	Area	Area (%)
1	8.93 min	3220.336	43.2
2	11.12 min	4226.854	56.8

Figure S4: HPLC chromatogram of (*R*)-**3p**

9. Alternative Cycle pathway for Acyl-Aryl Cross-Coupling from α-aminoaldehydes

Herein, we present an alternative cycle pathway for Acyl-Aryl Cross Coupling from α-aminoaldehydes (Scheme S1). The catalytic cycle begins with the oxidative addition of the aryl bromide to form the **A**-Ni(II) complex. The reaction then proceeds via energy transfer (EnT) from photoexcited *Ir(III) to intermediate **A**, generating the excited state **B**-[Ni(II)]*, which undergoes Ni–Br bond homolysis to produce the Br• radical and the **C**-Ni(I) complex. This radical promotes the hydrogen atom transfer (HAT) from α-amino aldehyde **2**, generating the acyl radical **2’** and HBr, which is promptly neutralized by K_2CO_3. The acyl radical **2’** subsequently adds to the **C**-Ni(I) complex, affording the **D**-Ni(II) intermediate, which may equilibrate with the complex **E**-Ni(II), followed by reductive elimination, leading to the formation of products **3** or **4**, while regenerating the nickel catalyst for the next catalytic cycle.

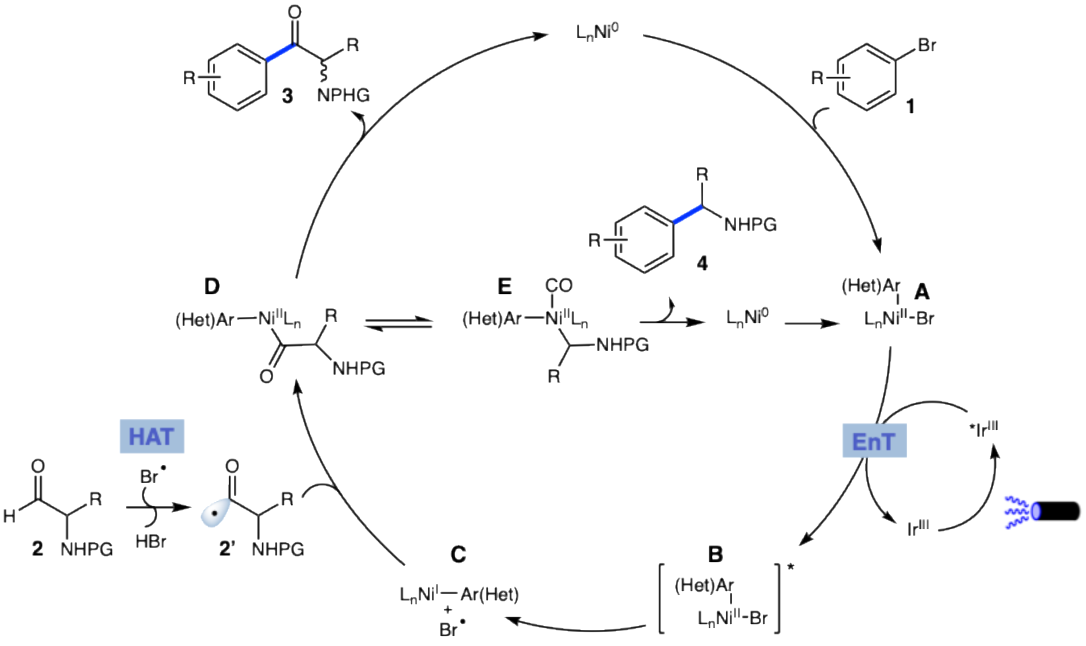

Scheme S1. Alternative mechanism for Acyl-Aryl Cross Coupling from α-aminoaldehydes

10. Spectra

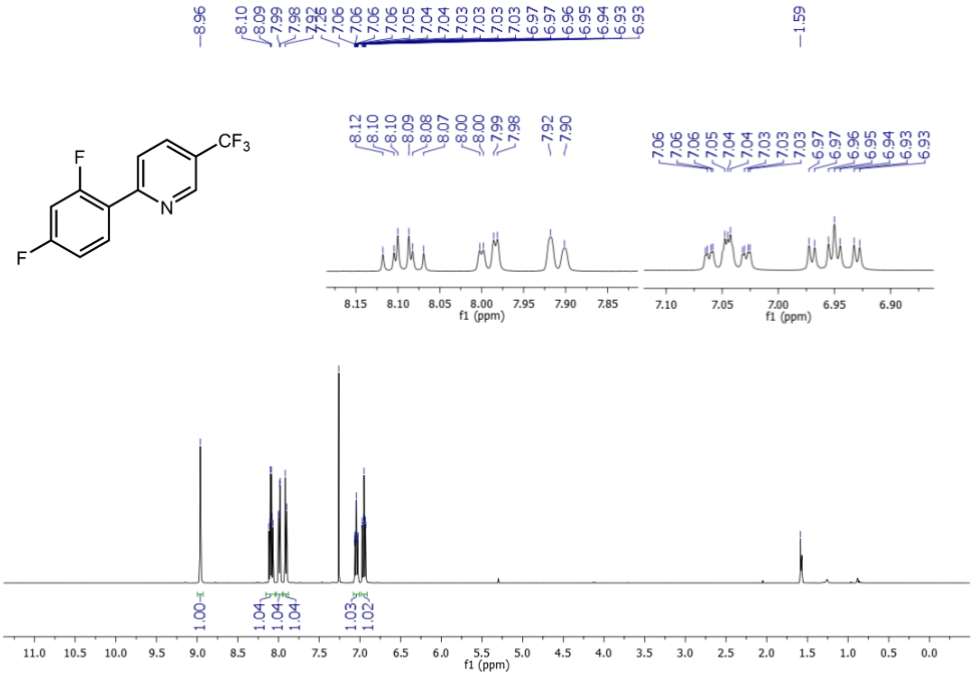

^{1}H NMR (500 MHz, CDCl$_3$) of ligand 2-(2,4-difluorophenyl)-5-(trifluoromethyl)pyridine

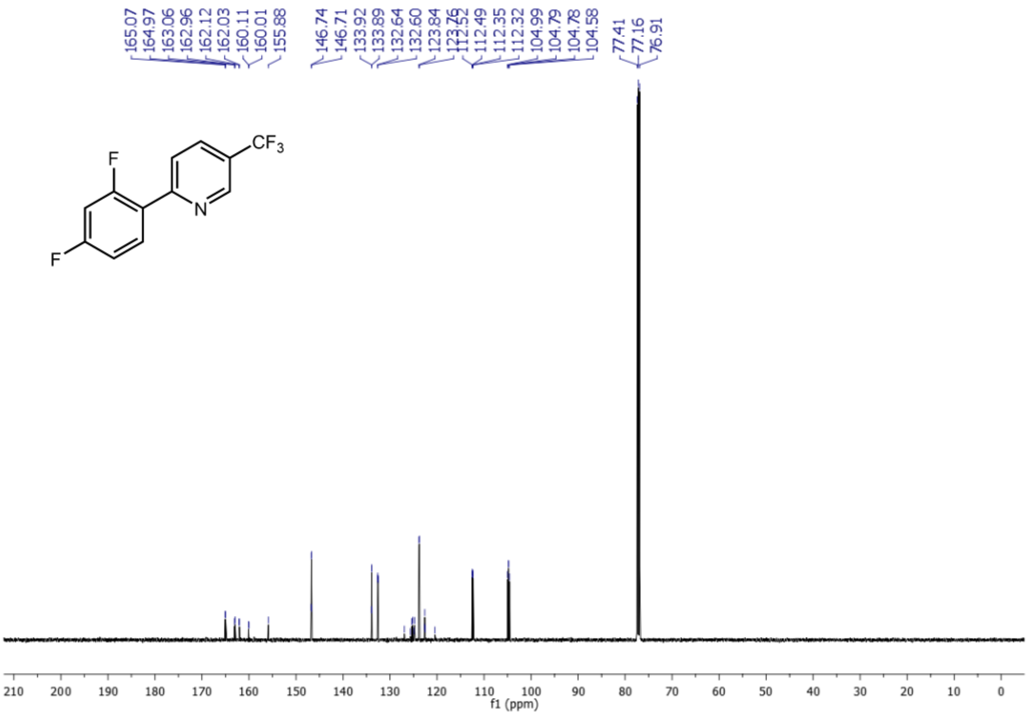

^{13}C NMR (126 MHz, CDCl$_3$) of ligand 2-(2,4-difluorophenyl)-5-(trifluoromethyl)pyridine

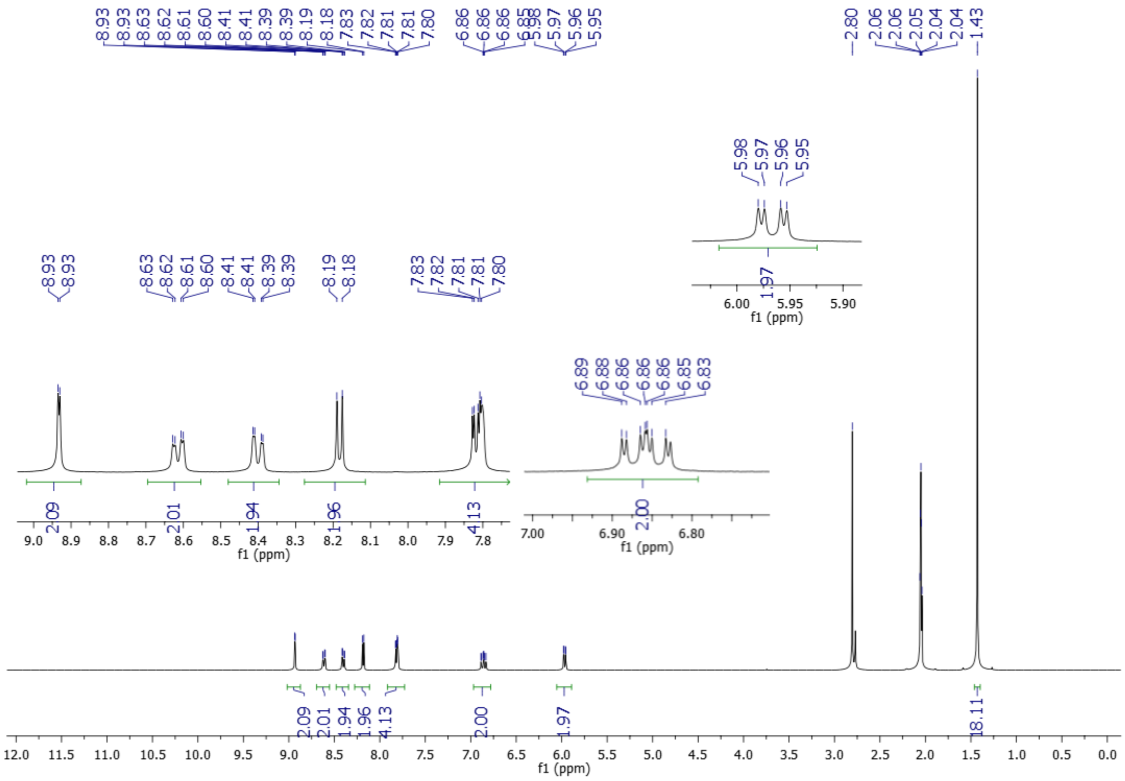

^{1}H NMR (400 MHz, $(CD_3)_2CO$) of photocatalyst $[Ir(dF(CF_3)ppy)_2(dtbbpy)](PF_6)$

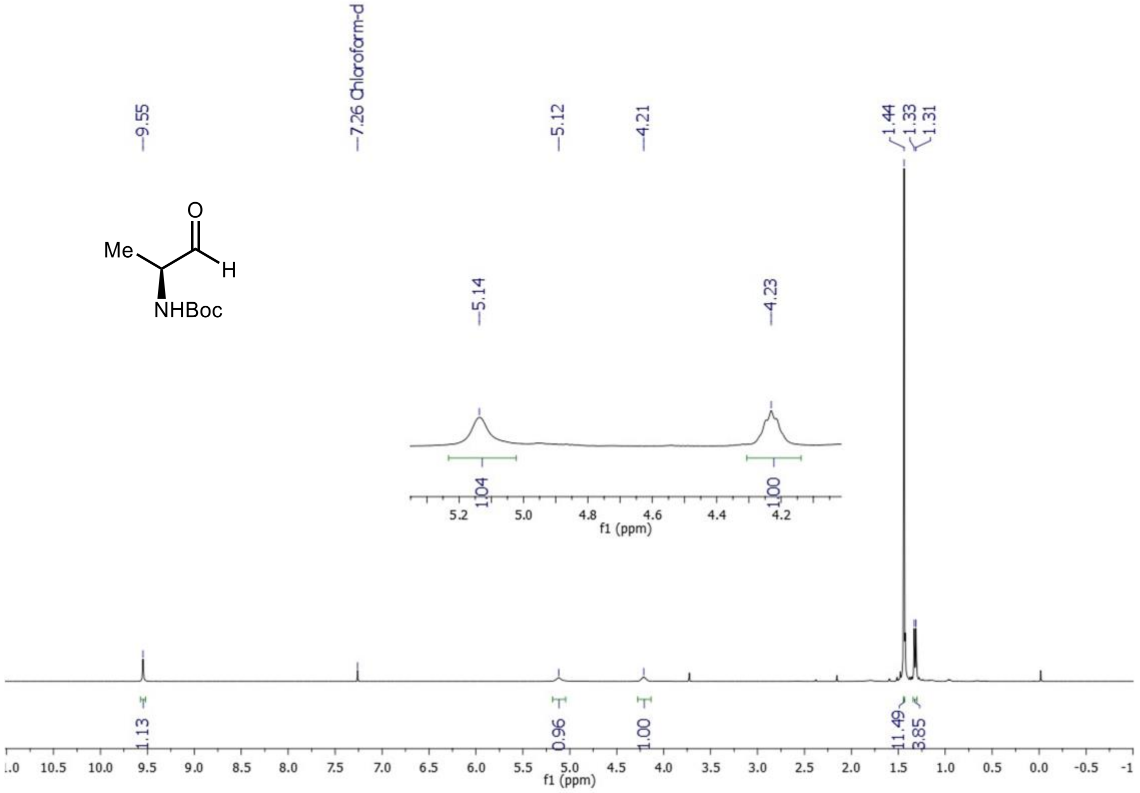

^{1}H NMR (400 MHz, $CDCl_3$) of compound 2a.

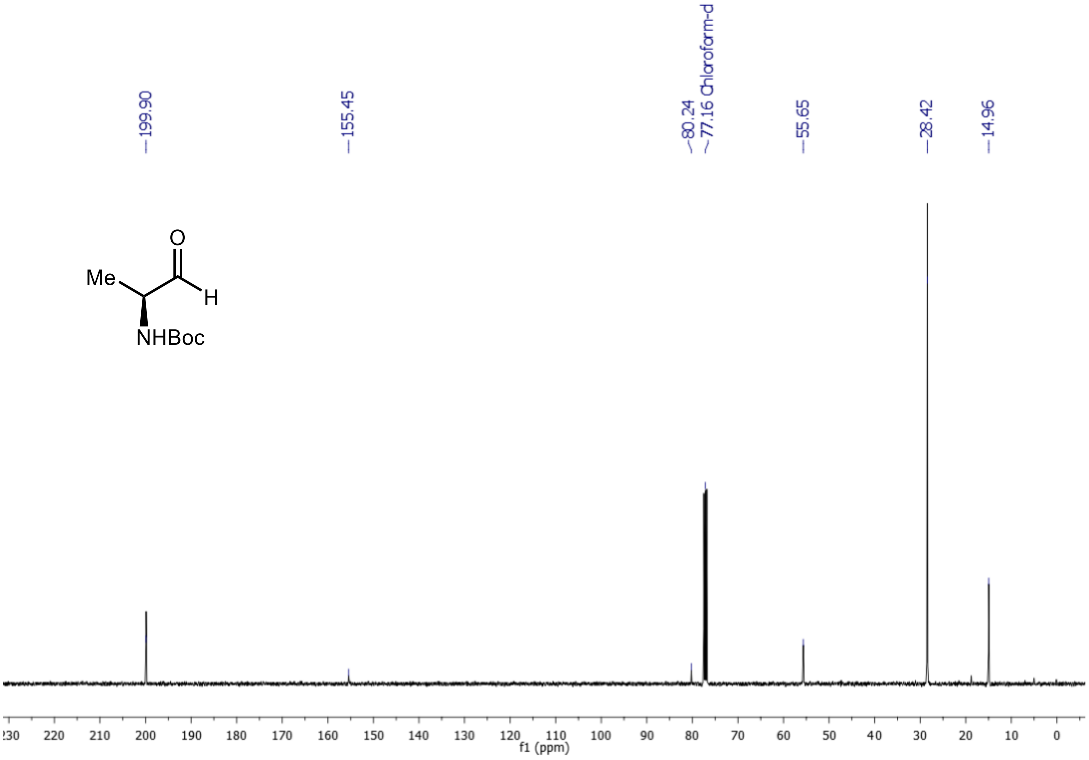

^{13}C NMR (101MHz, $CDCl_3$) of compound 2a.

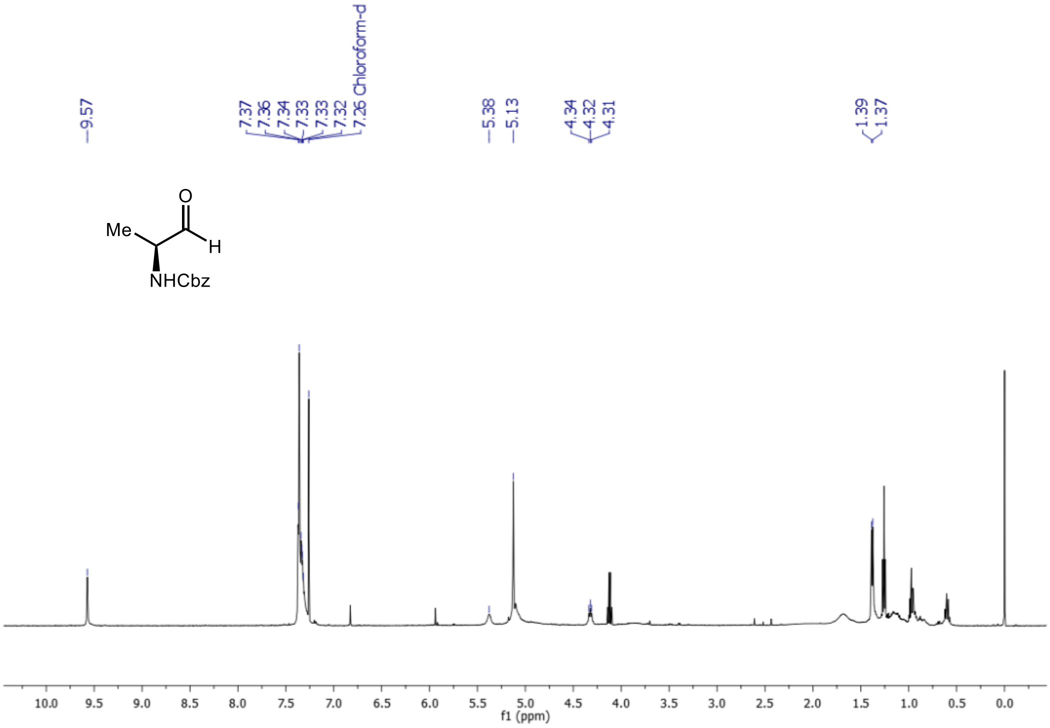

^{1}H NMR (500 MHz, $CDCl_3$) of compound 2p.

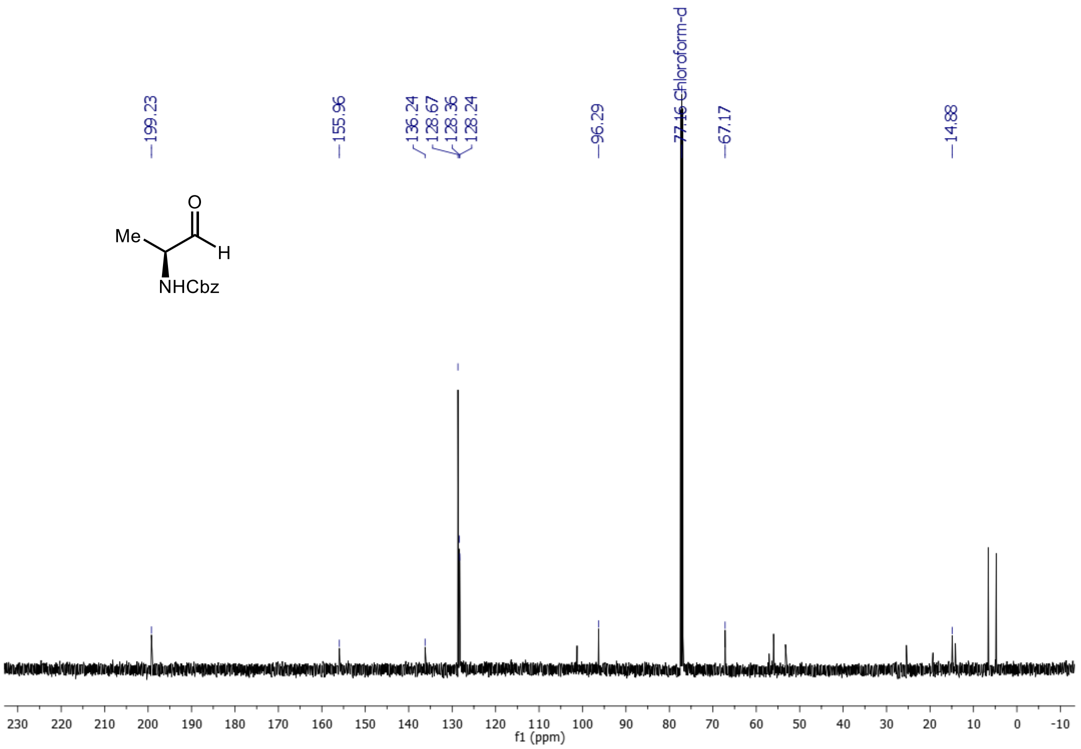

^{13}C NMR (126 MHz, $CDCl_3$) of compound 2p.

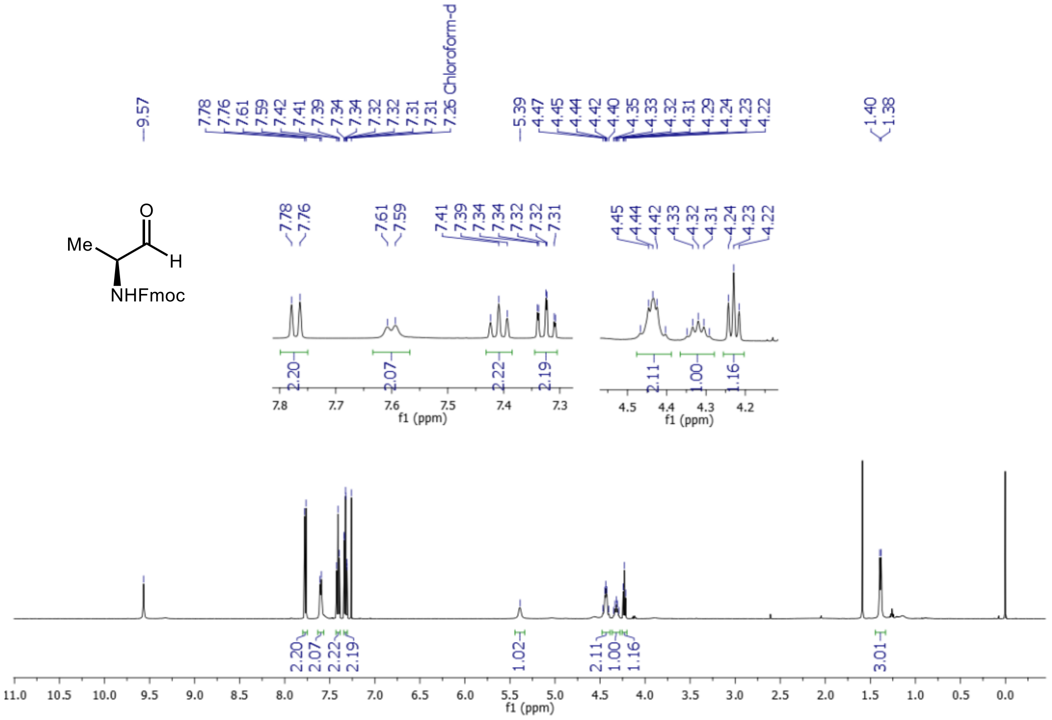

^{1}H NMR (500 MHz, $CDCl_3$) of compound 2q.

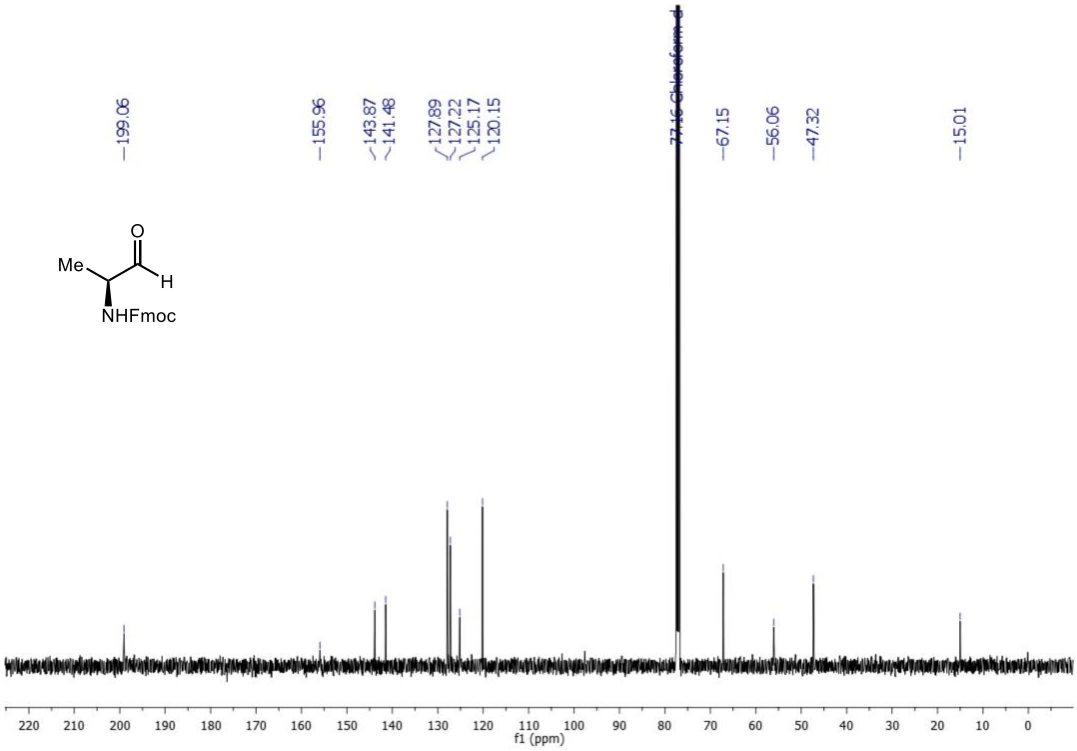

^{13}C **NMR (126 MHz, $CDCl_3$) of compound 2q.**

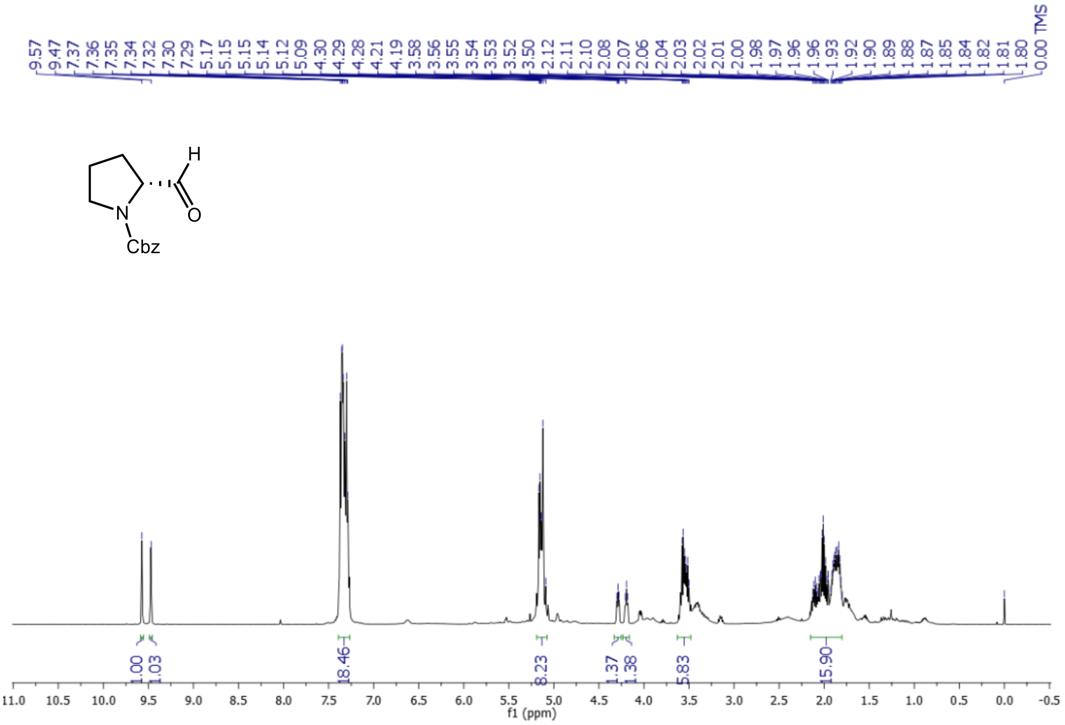

^{1}H **NMR (500 MHz, $CDCl_3$) of compound 2r.**

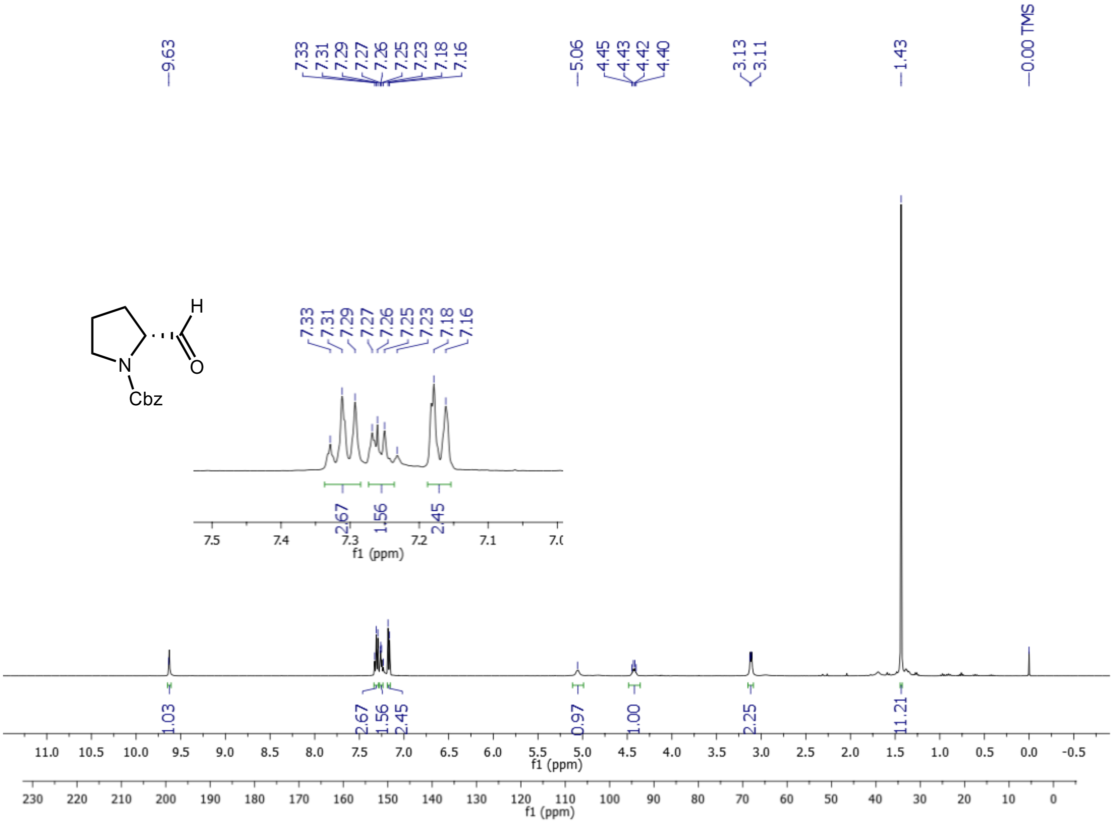

^{13}C NMR (126 MHz, CDCl$_3$) of compound 2t.

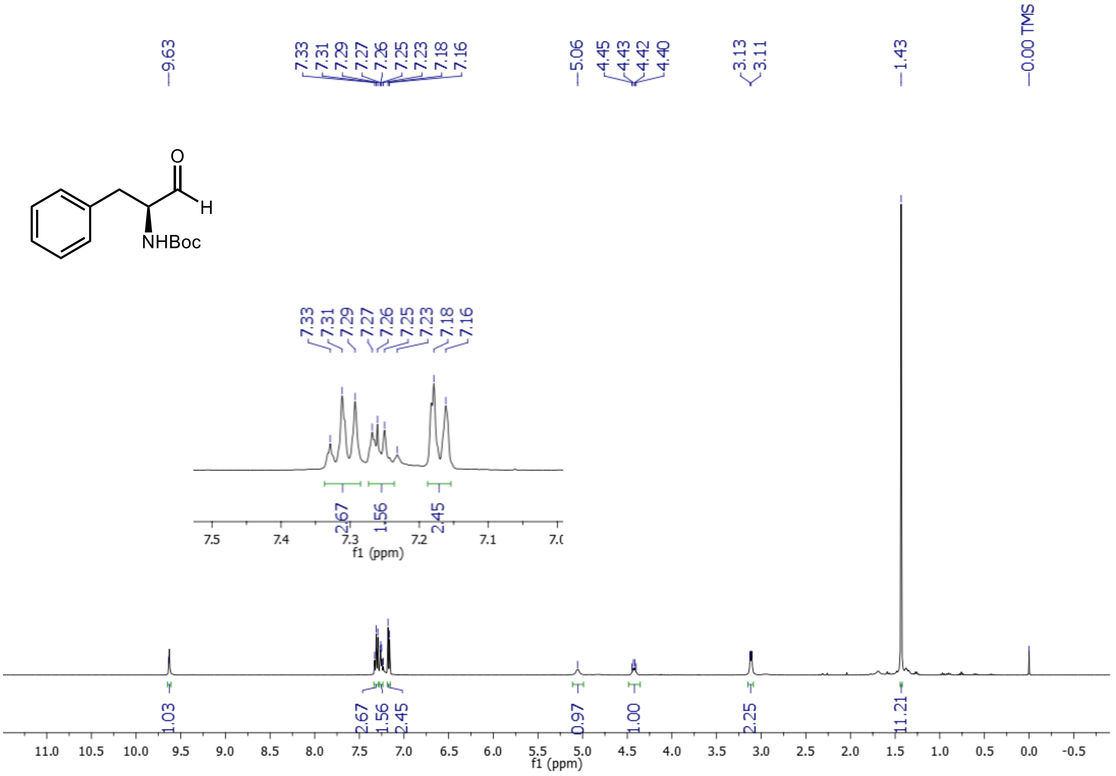

^{1}H NMR (400 MHz, CDCl$_3$) of compound 2s.

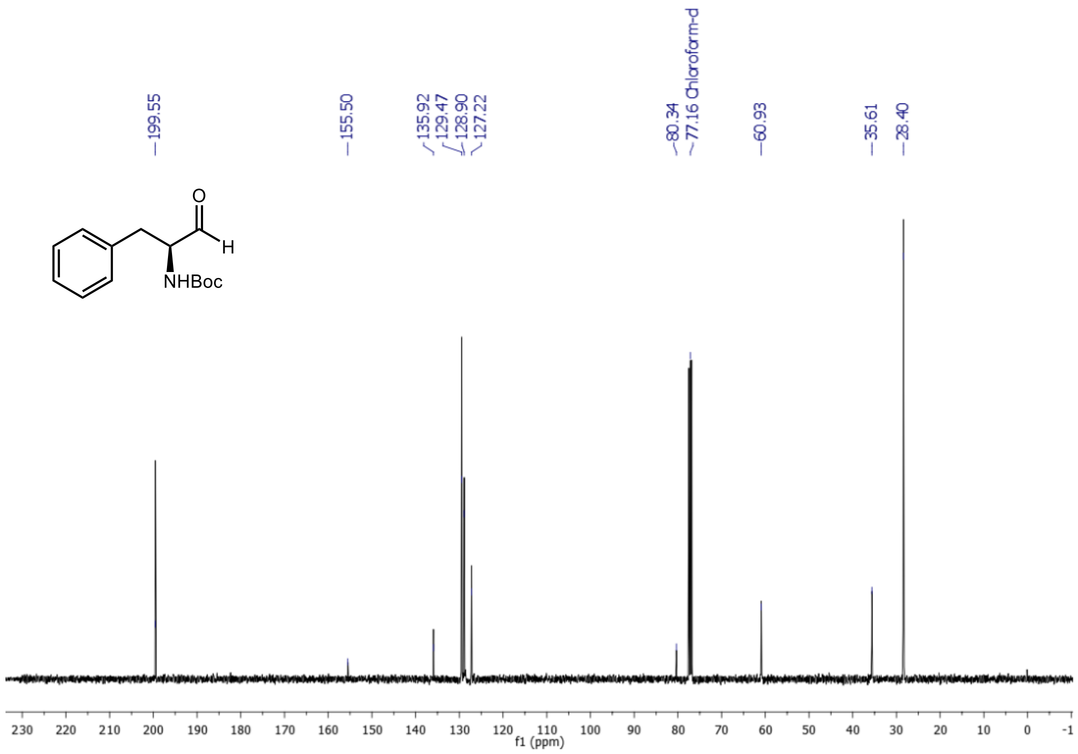

^{13}C NMR (126 MHz, CDCl$_3$) of compound 2s.

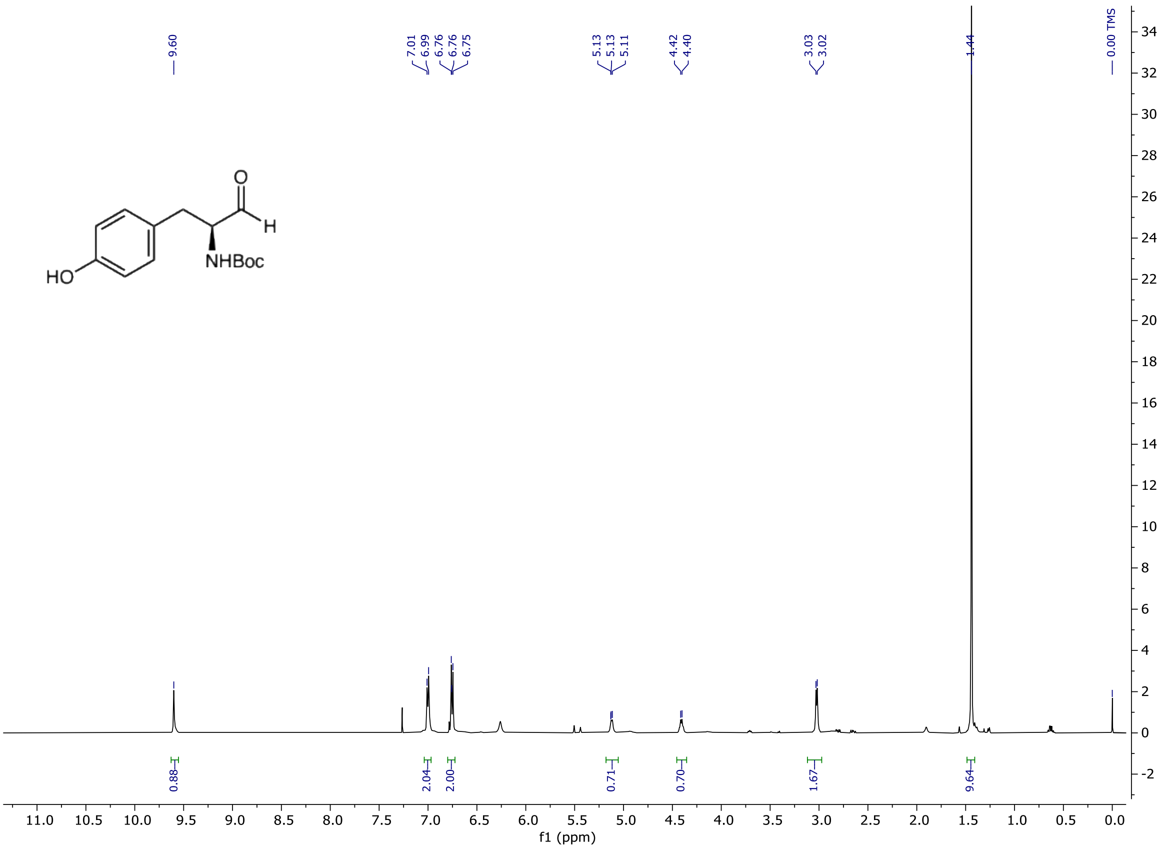

^{1}H NMR (400 MHz, CDCl$_3$) of compound 2t.

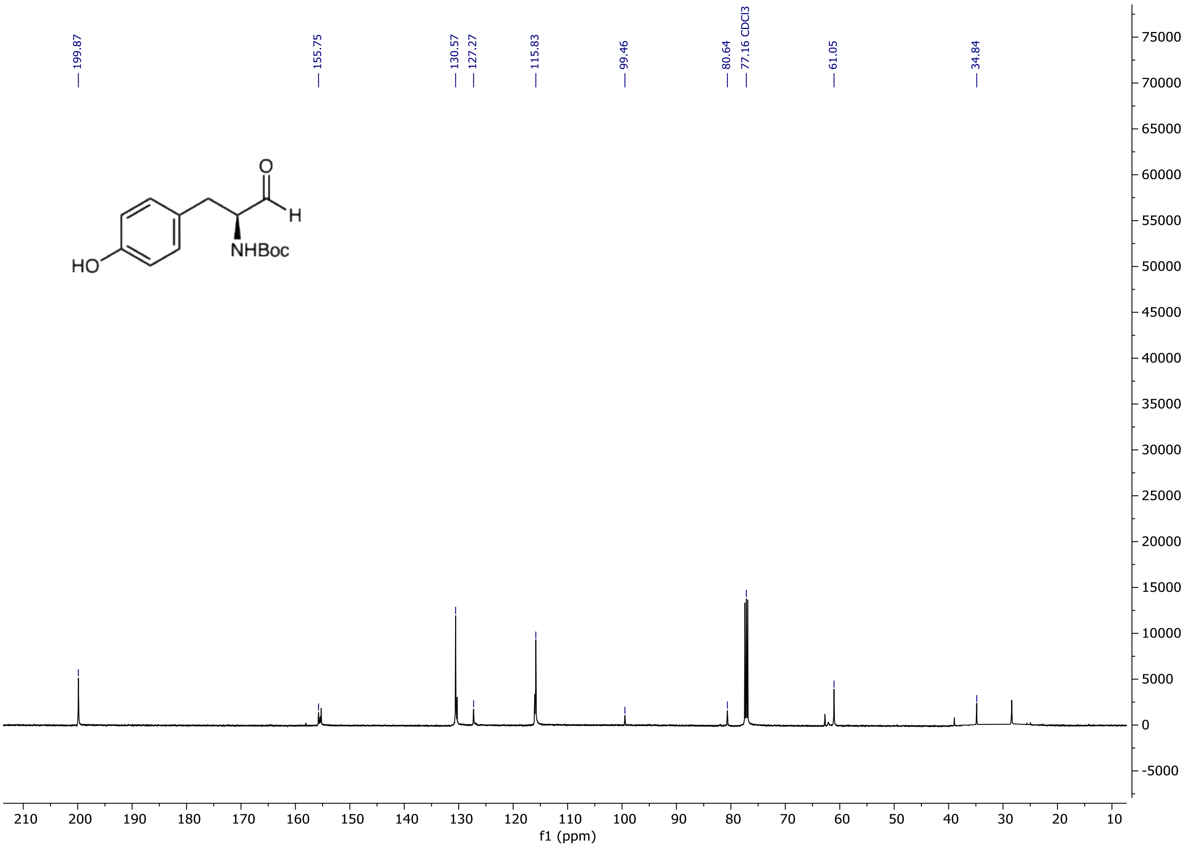

^{13}C NMR (126 MHz, CDCl$_3$) of compound 2t.

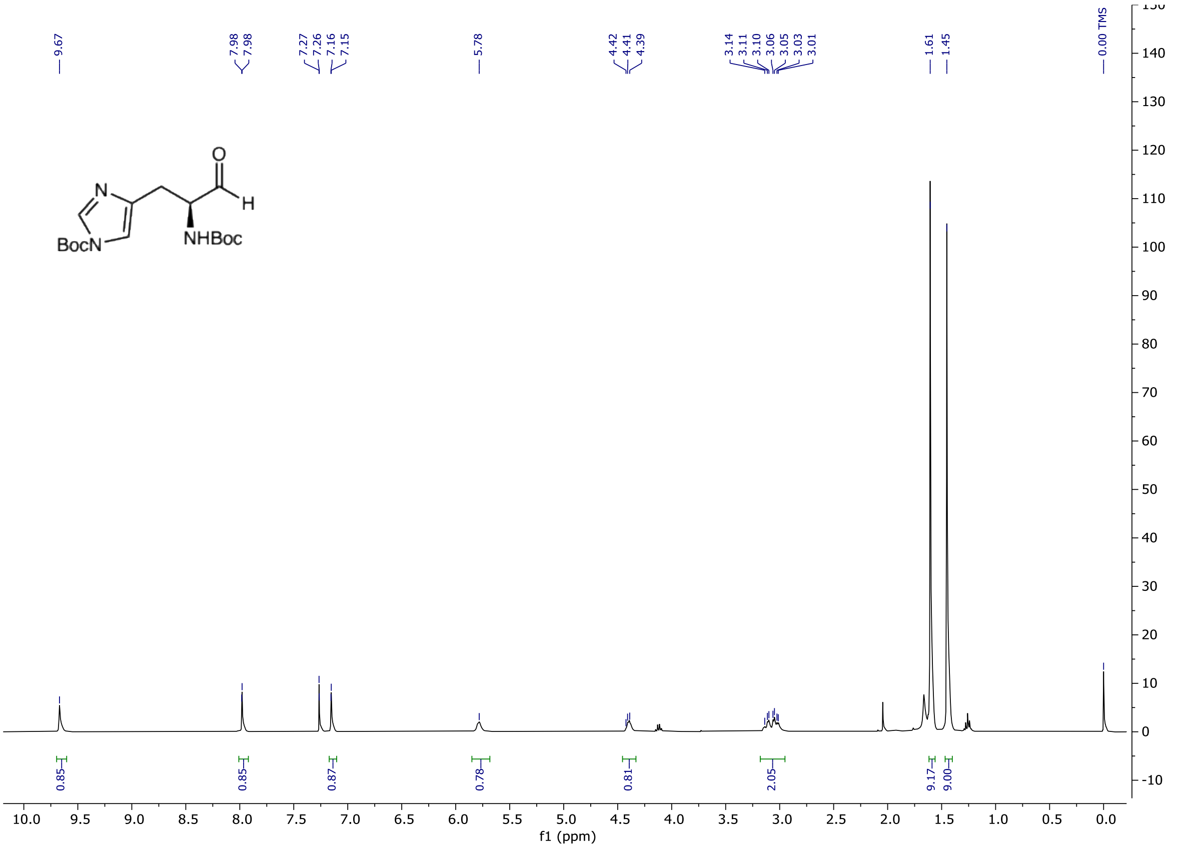

^{1}H NMR (500 MHz, CDCl$_3$) of compound 2u.

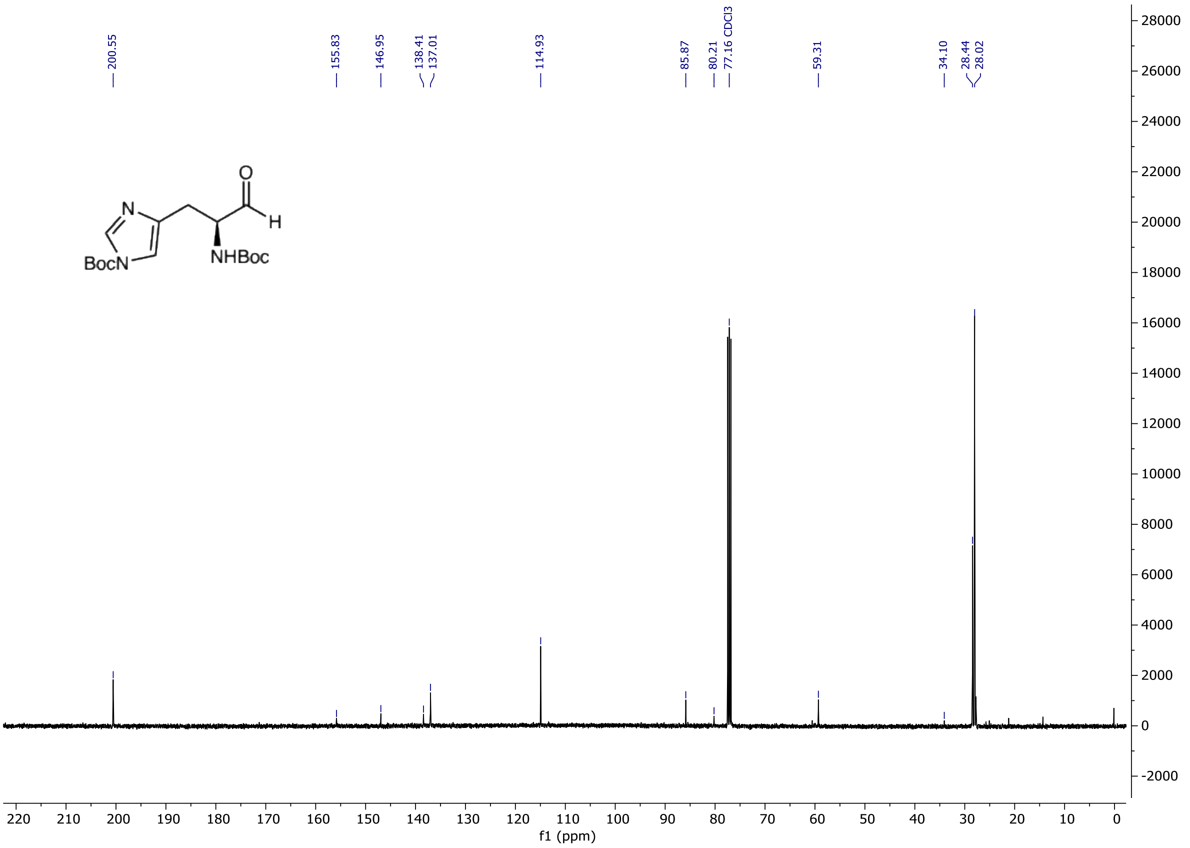

^{13}C NMR (126 MHz, $CDCl_3$) of compound 2u.

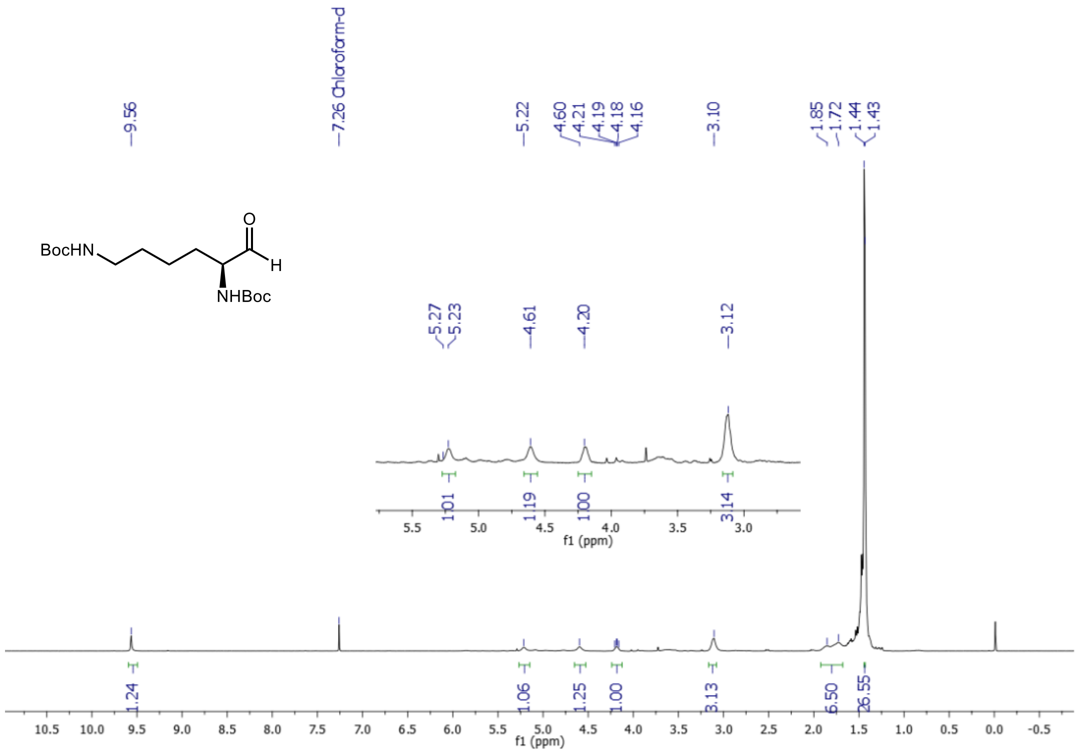

^{1}H NMR (400 MHz, $CDCl_3$) of compound 2v.

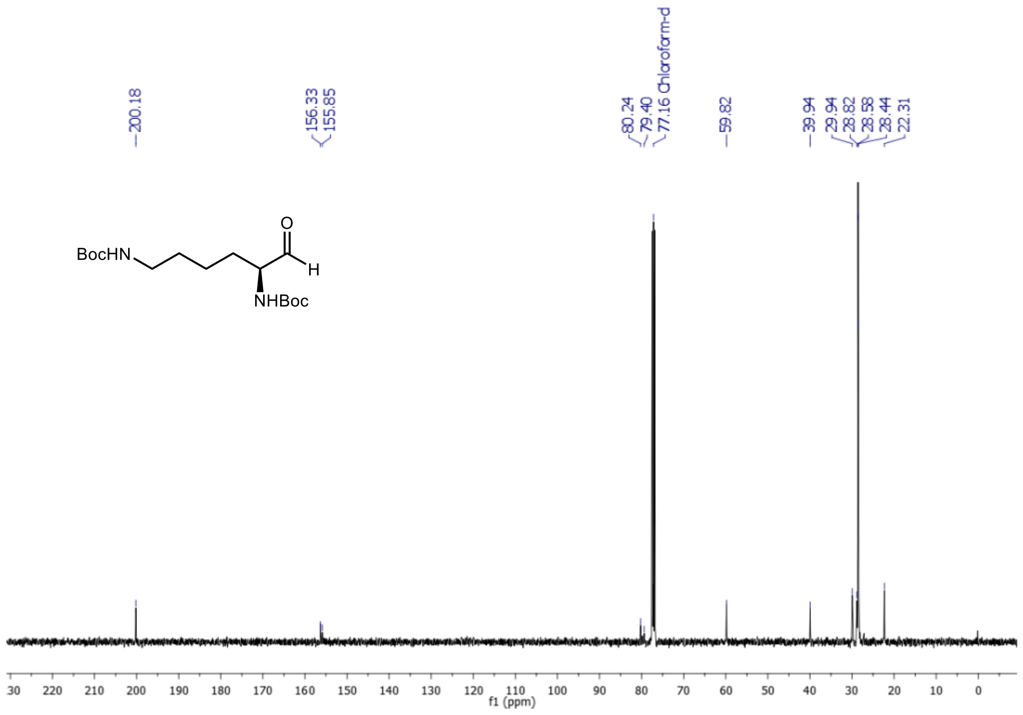

^{13}C NMR (101 MHz, $CDCl_3$) of compound 2v.

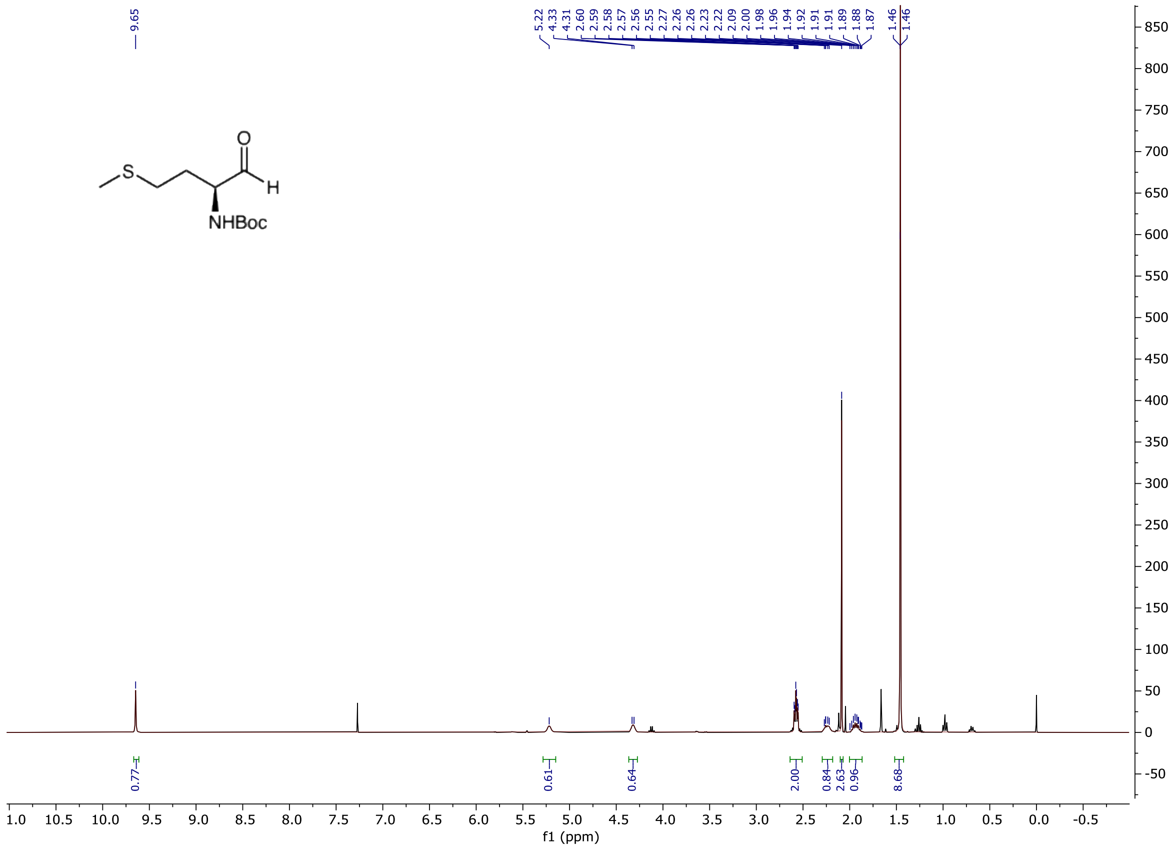

^{1}H NMR (400 MHz, $CDCl_3$) of compound 2

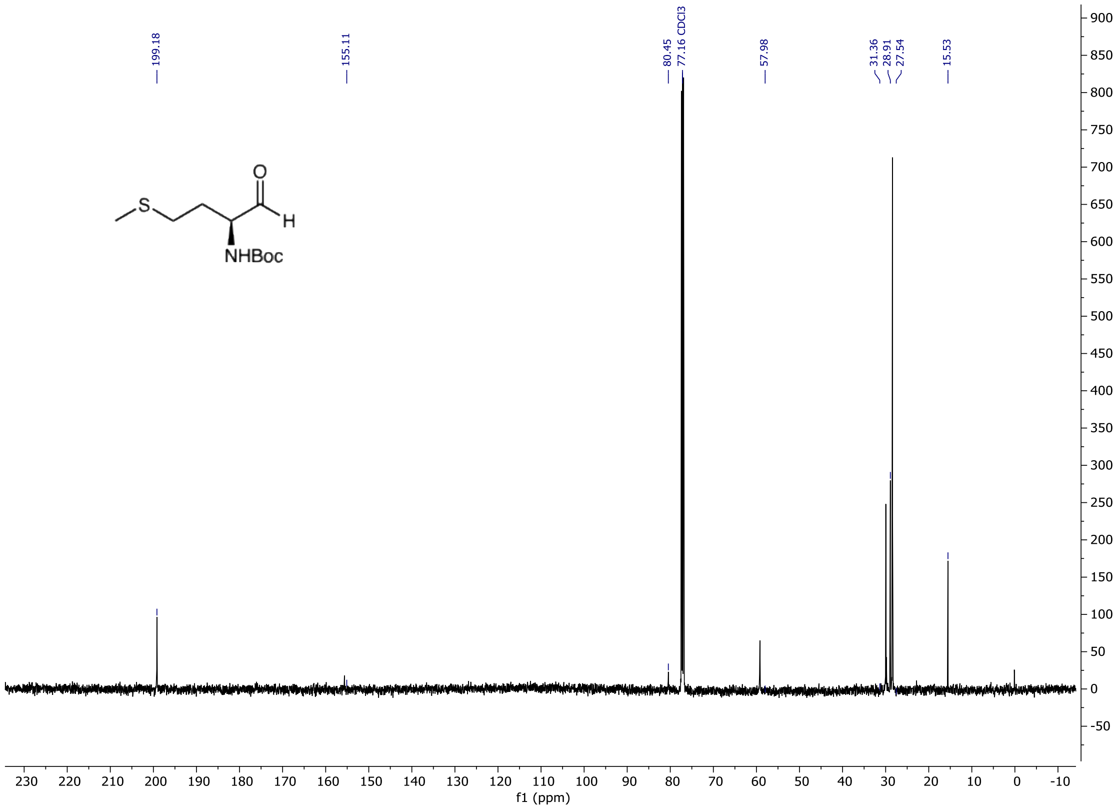

^{13}C NMR (126 MHz, $CDCl_3$) of compound 2x.

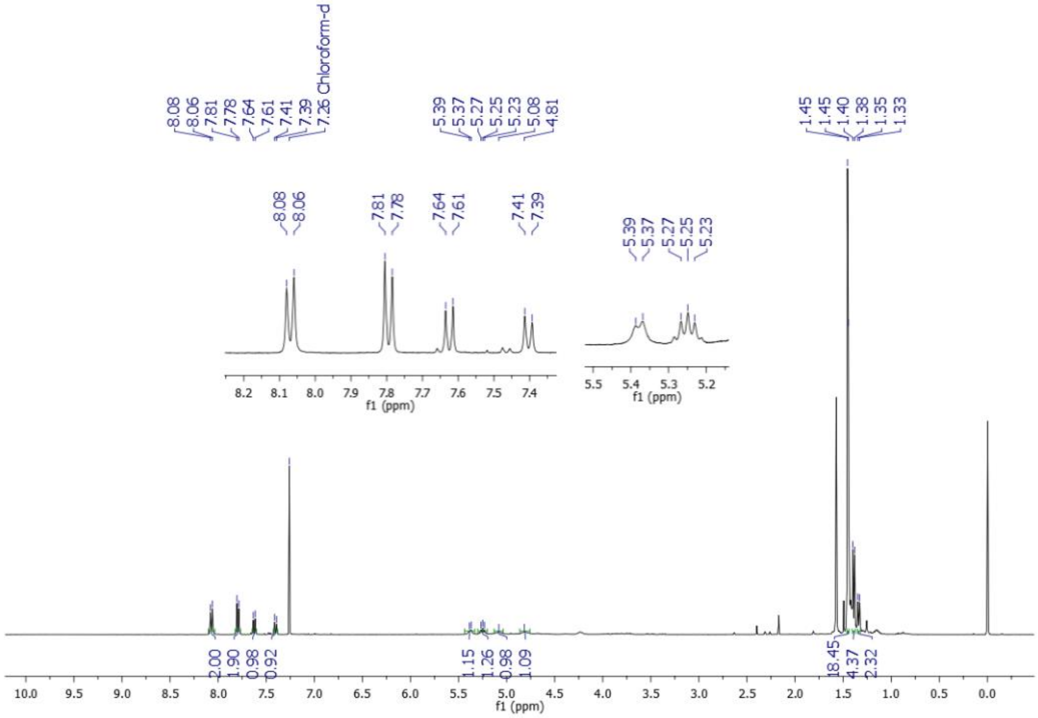

^{1}H NMR (500 MHz, $CDCl_3$) of compounds 3a + 4a.

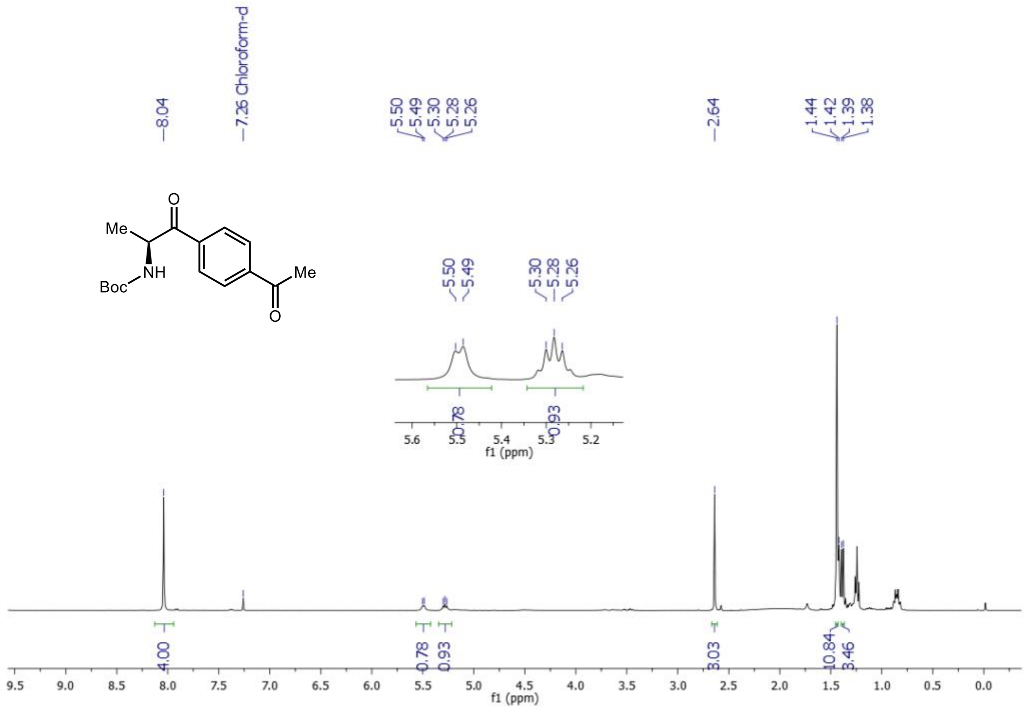

^{1}H NMR (500 MHz, $CDCl_3$) of compound 3b.

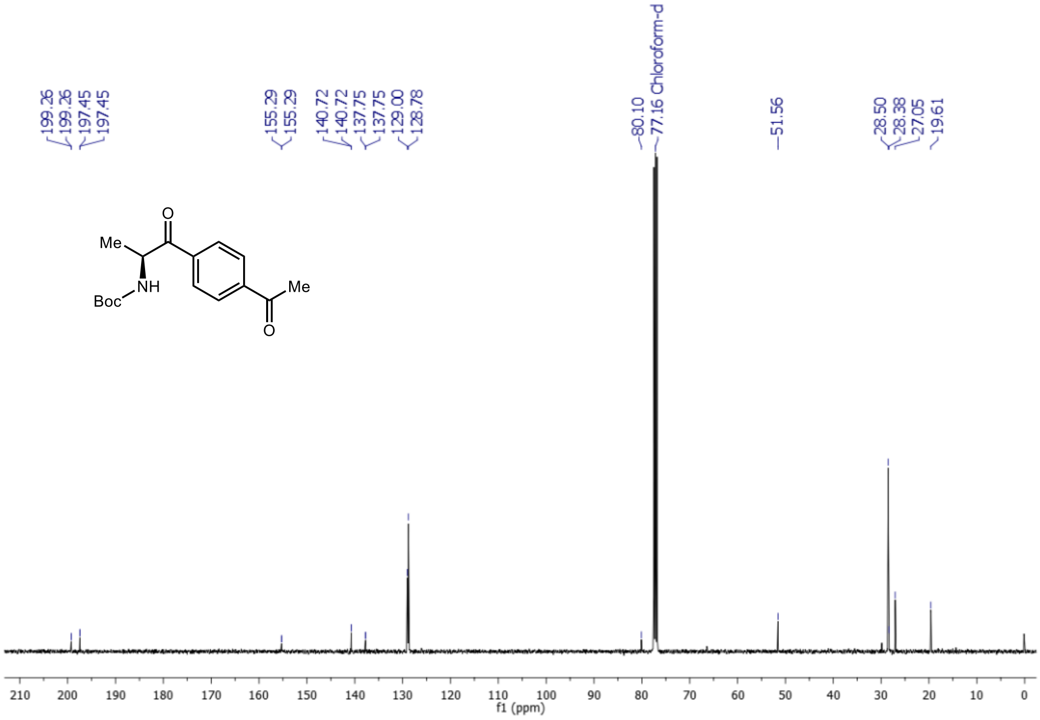

^{13}C NMR (126 MHz, $CDCl_3$) of compound 3b.

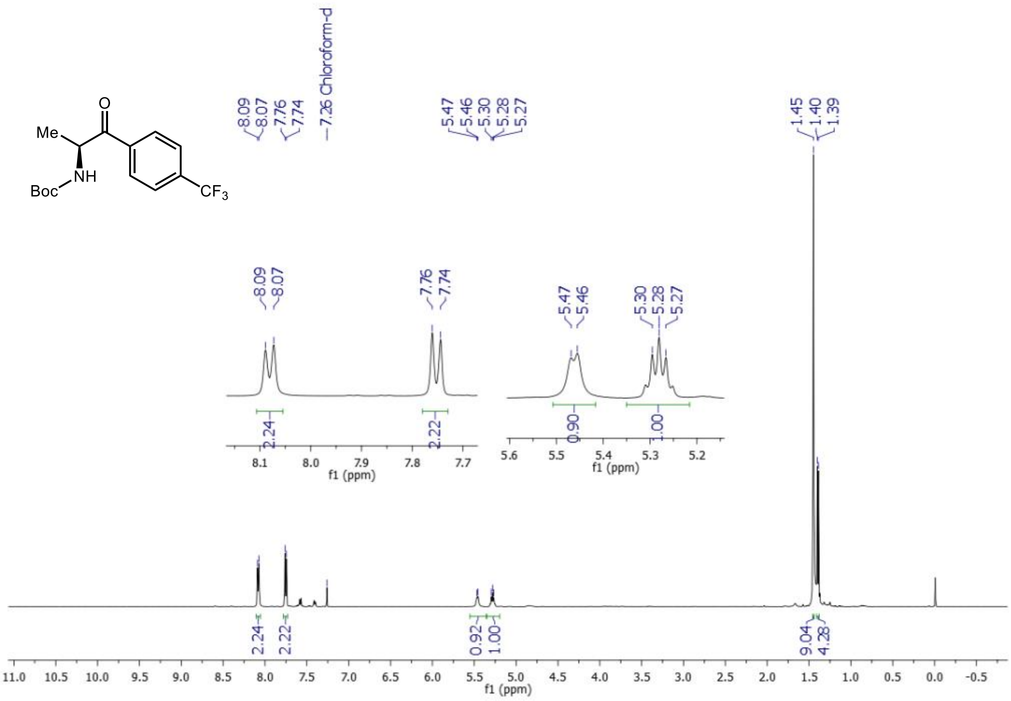

^{1}H NMR (500 MHz, $CDCl_3$) of compound 3c.

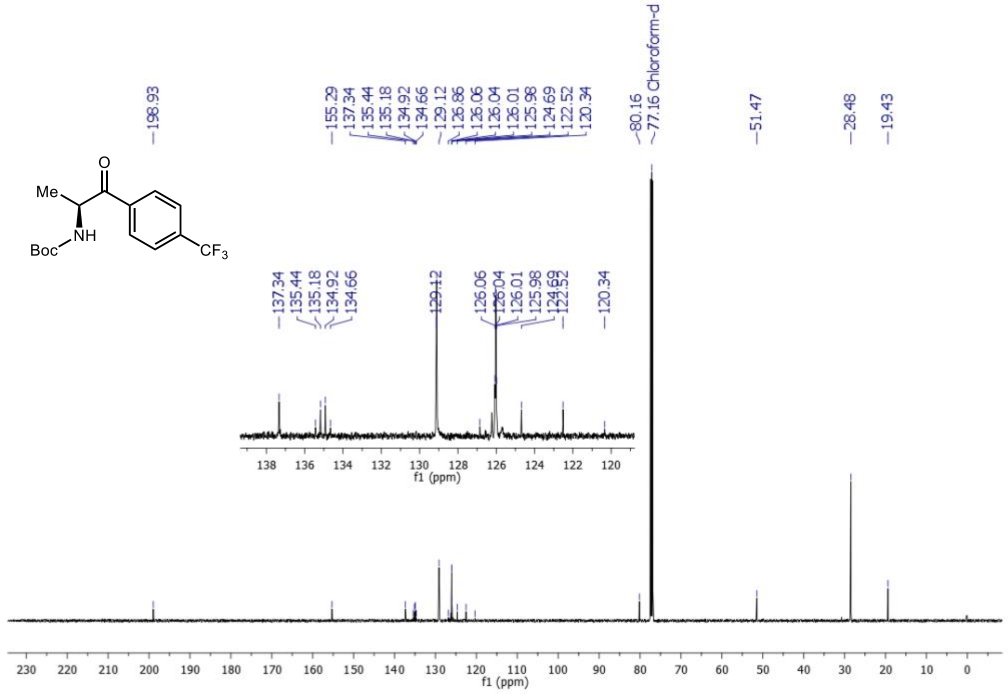

^{13}C NMR (126 MHz, $CDCl_3$) of compound 3c.

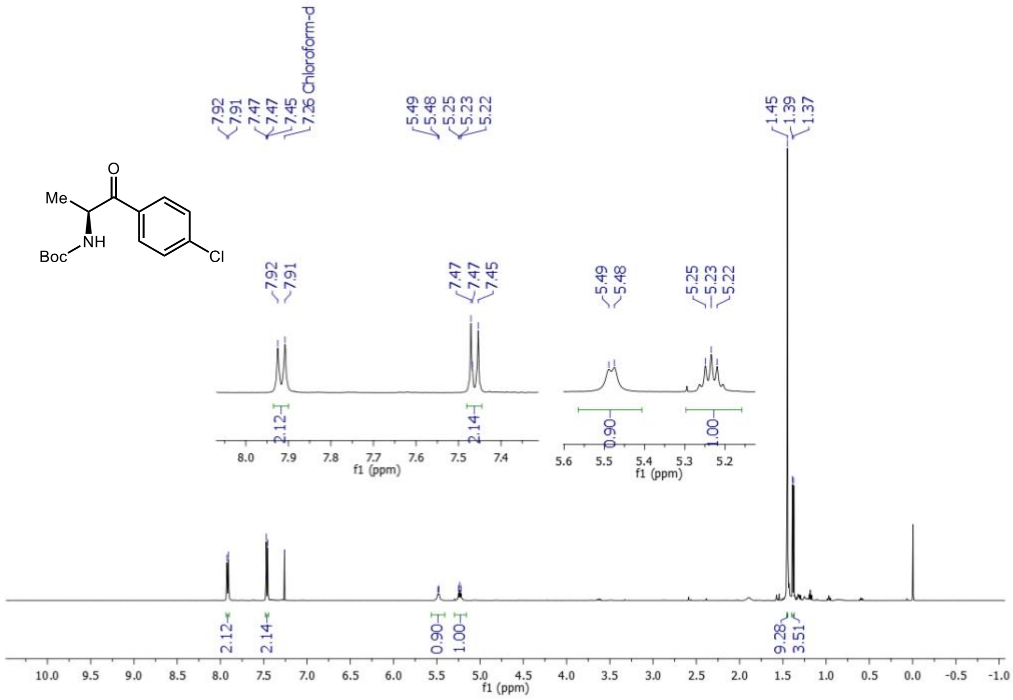

^{1}H NMR (500 MHz, $CDCl_3$) of compound 3d.

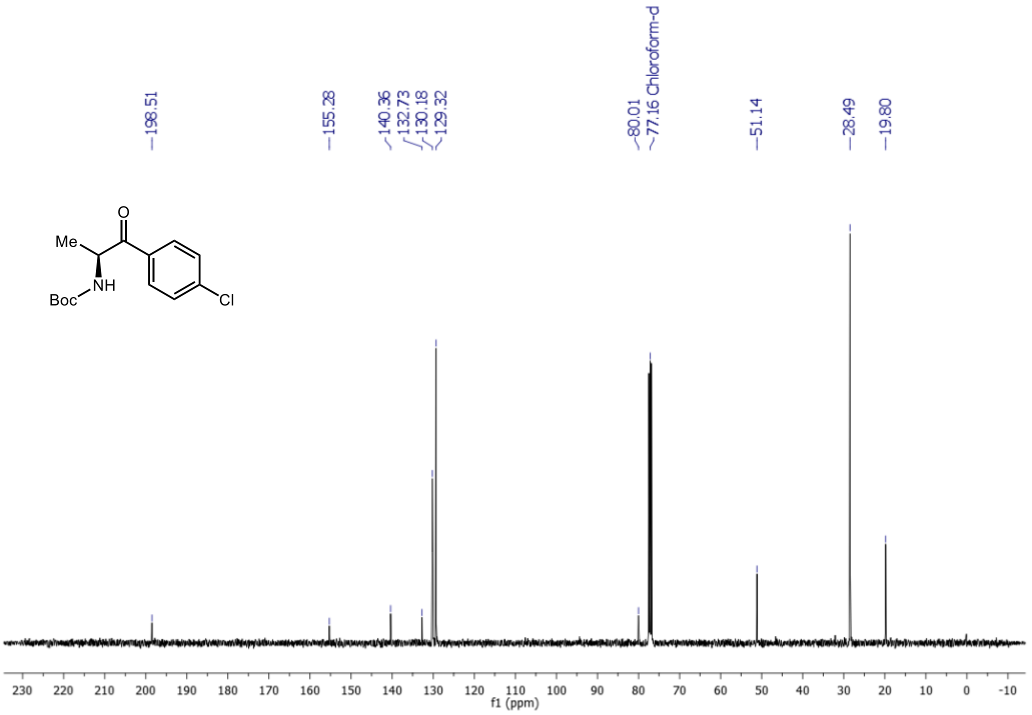

^{13}C NMR (126 MHz, $CDCl_3$) of compound 3d.

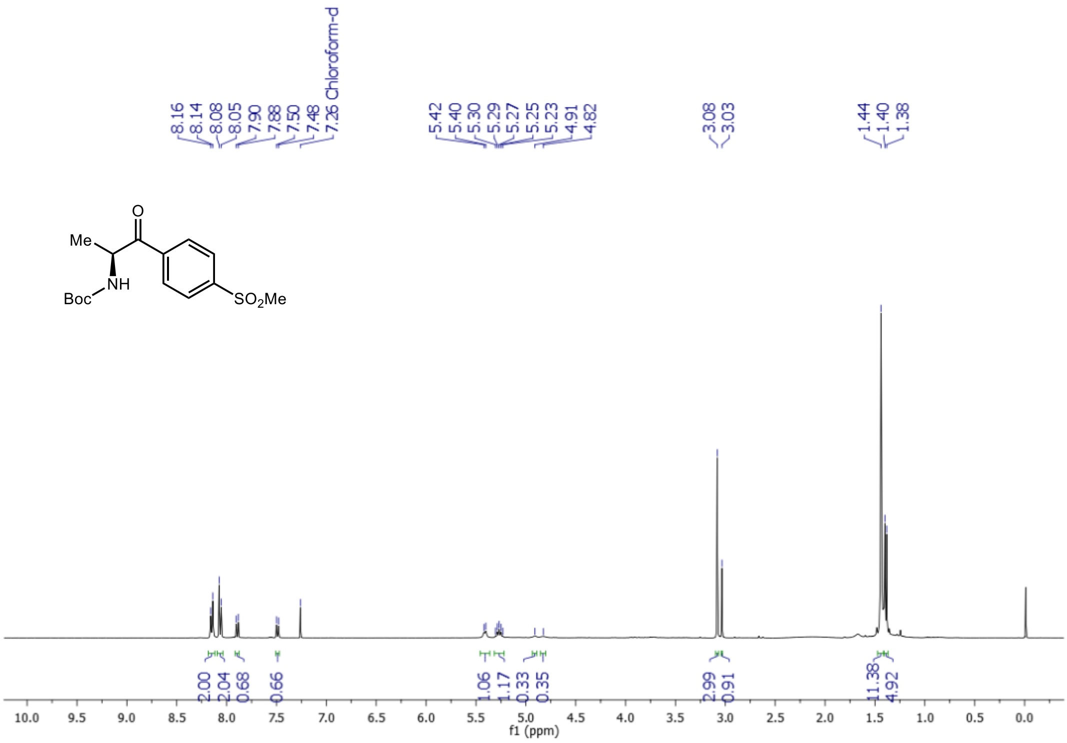

^{1}H NMR (500 MHz, $CDCl_3$) of compounds 3e + 4e.

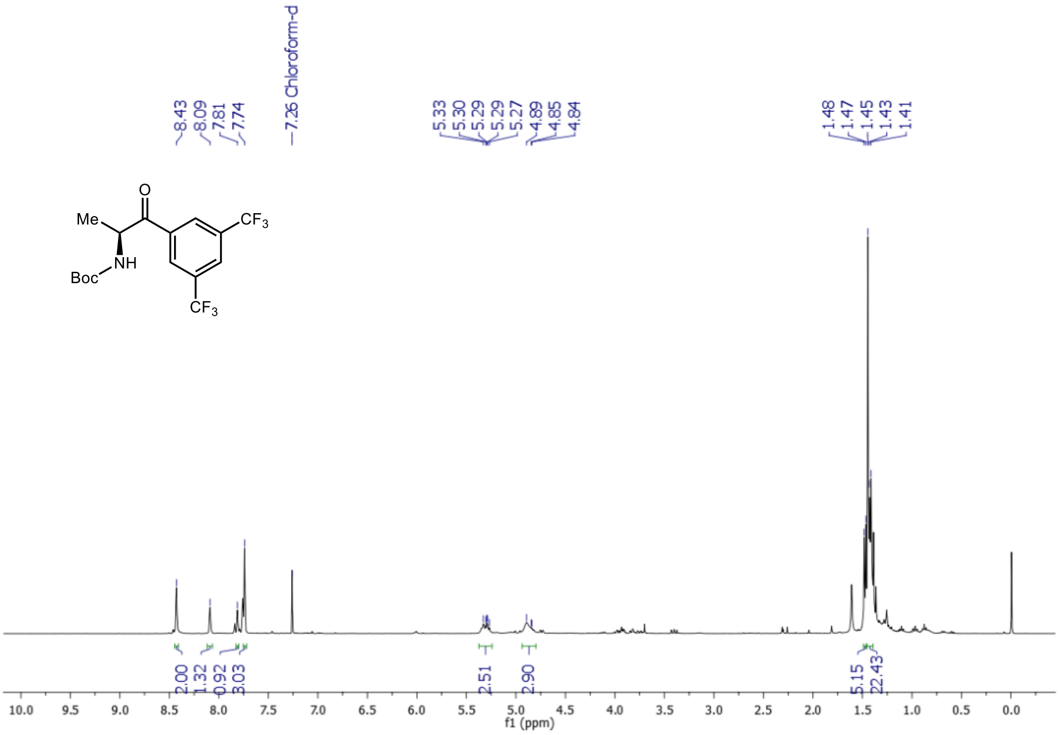

^{1}H NMR (500 MHz, $CDCl_3$) of compounds 3f + 4f.

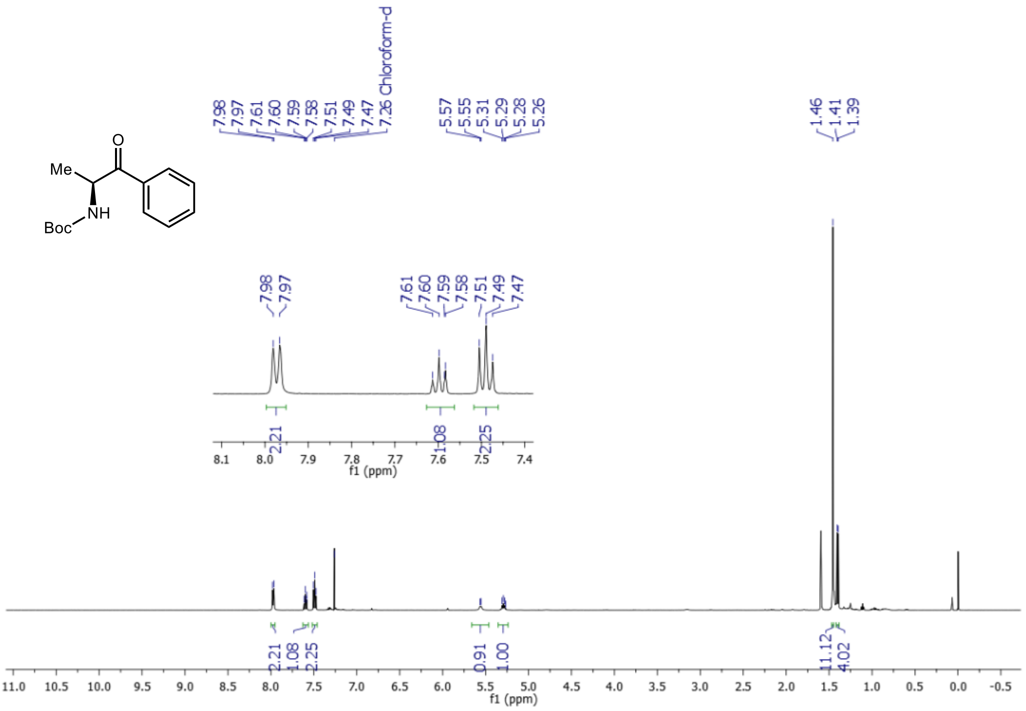

^{1}H NMR (500 MHz, $CDCl_3$) of compound 3g.

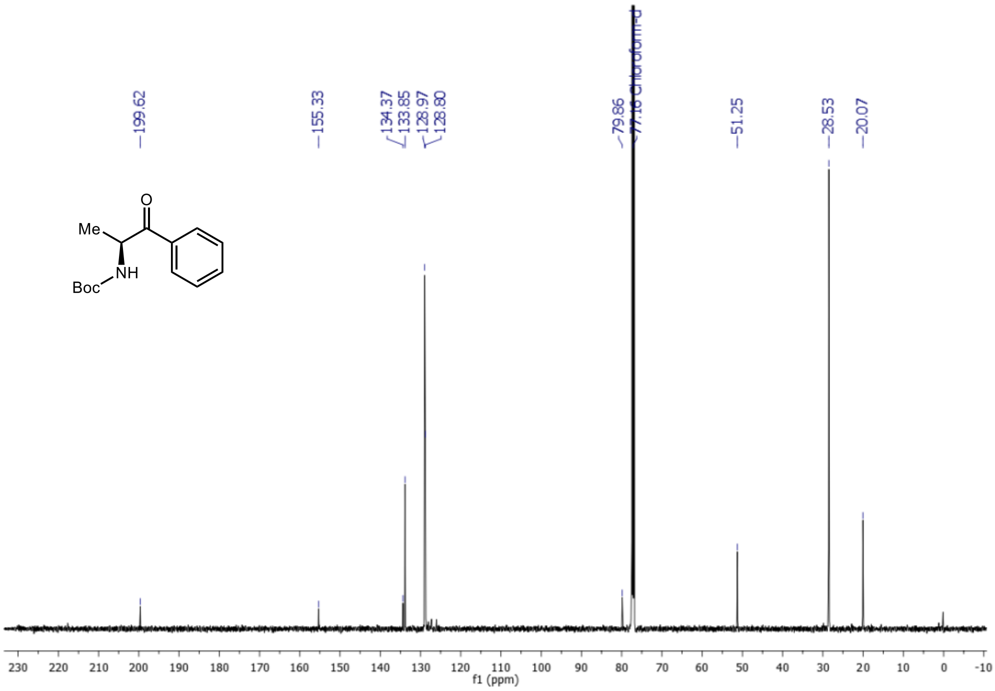

^{13}C NMR (126 MHz, $CDCl_3$) of compound 3g.

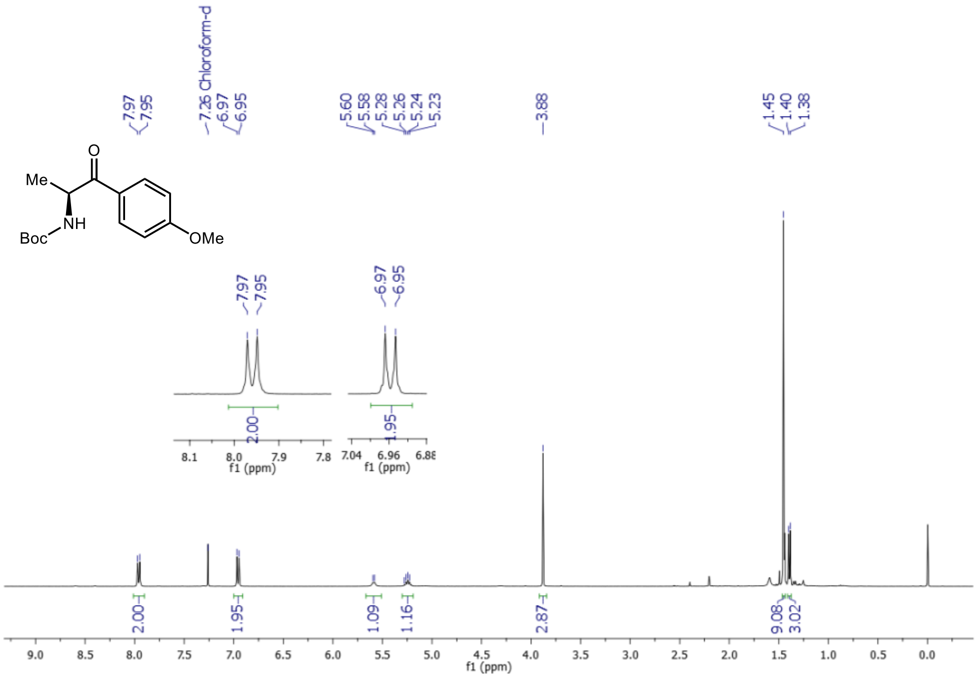

^{1}H NMR (500 MHz, $CDCl_3$) of compound 3h.

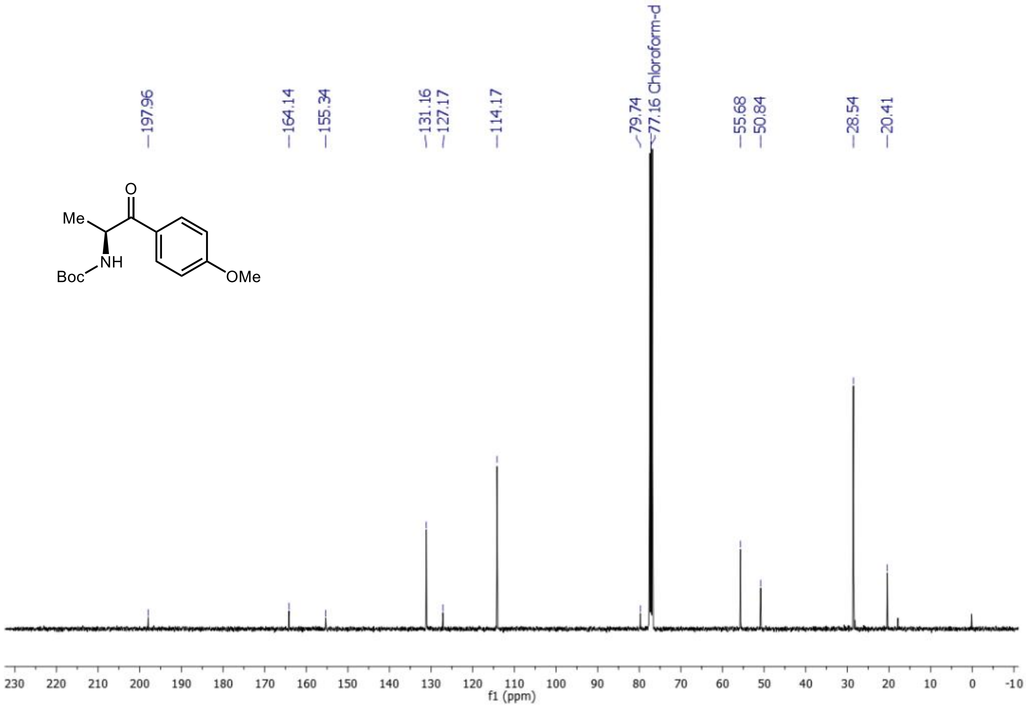

^{13}C NMR (126 MHz, $CDCl_3$) of compound 3h.

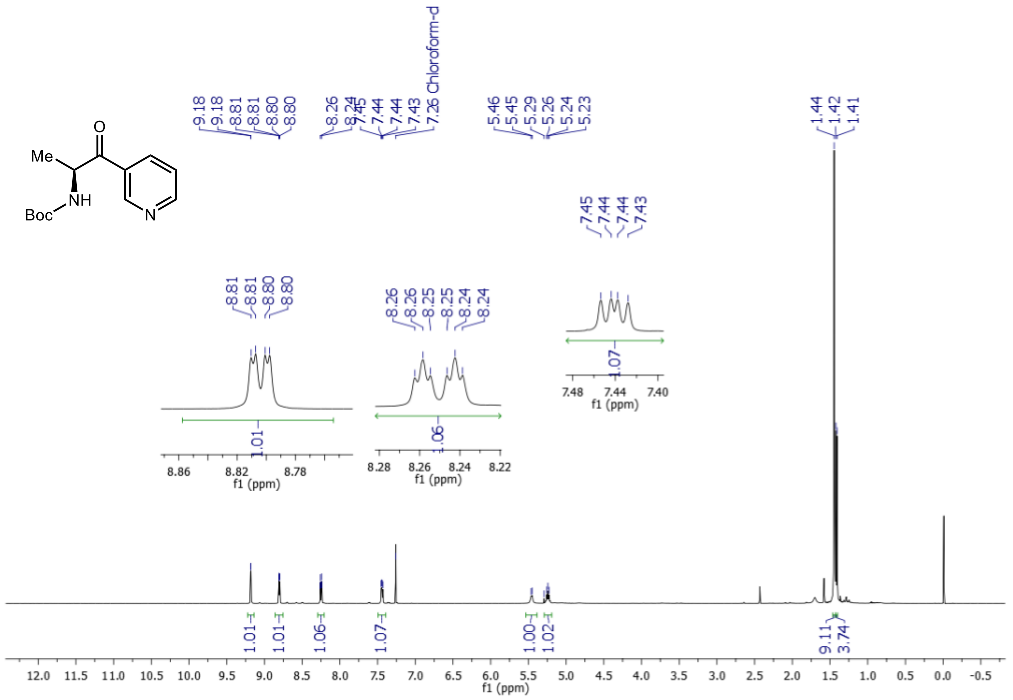

^{1}H NMR (500 MHz, $CDCl_3$) of compound 3j.

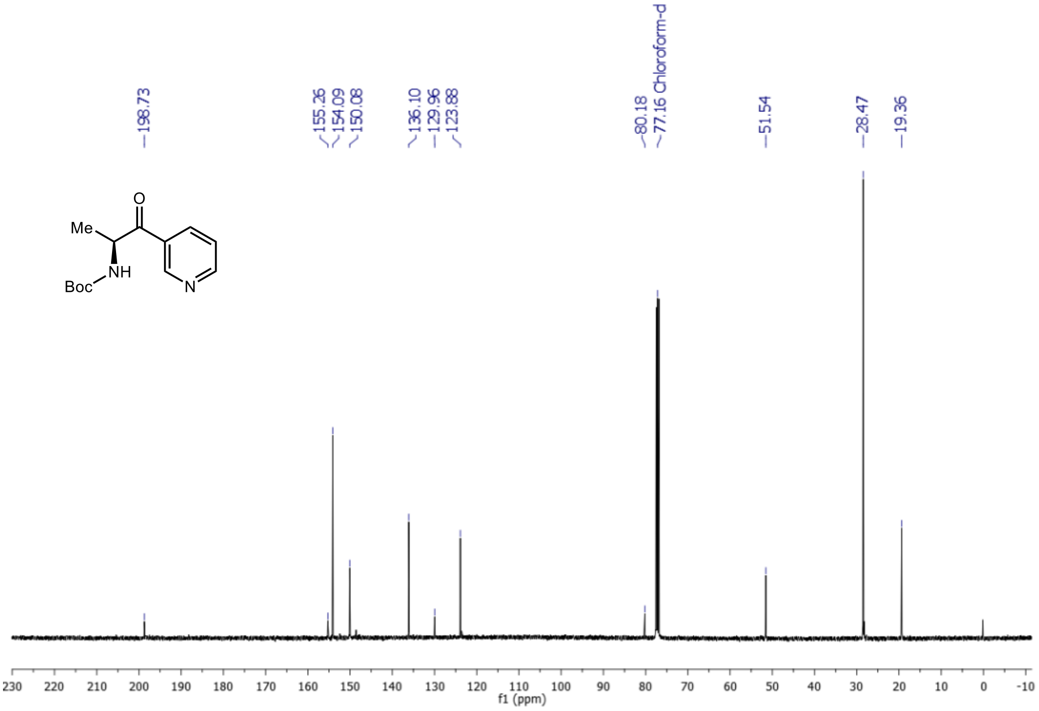

^{13}C NMR (126 MHz, $CDCl_3$) of compound 3j.

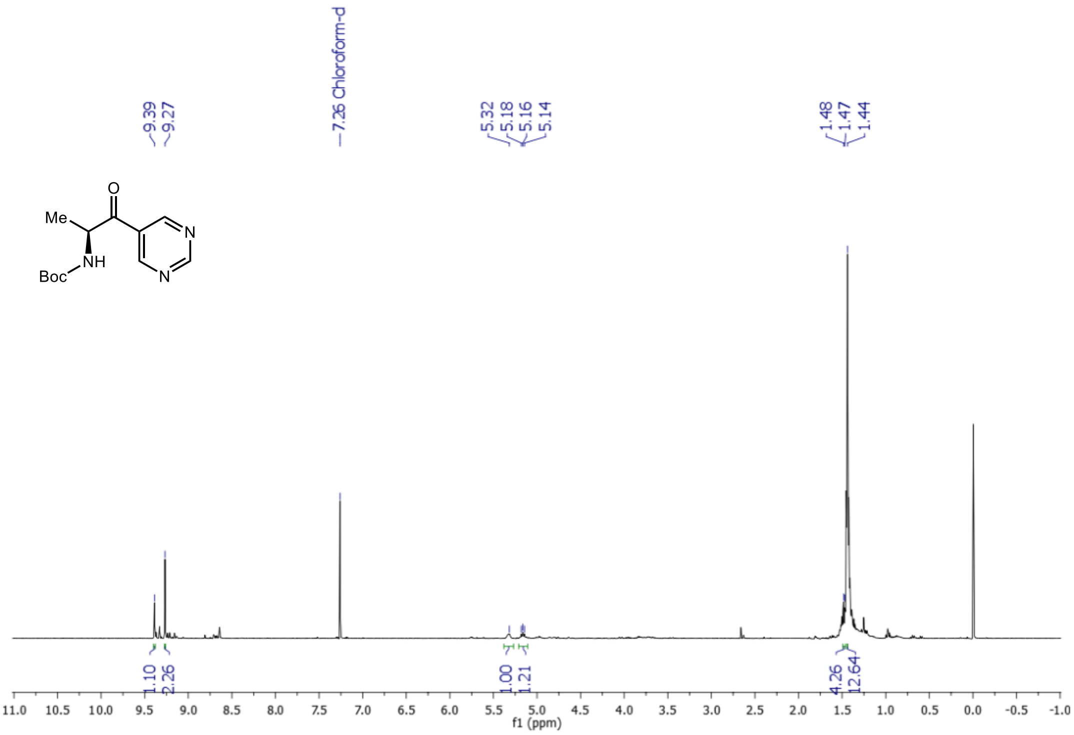

^{1}H NMR (500 MHz, CDCl$_3$) of compound 3k.

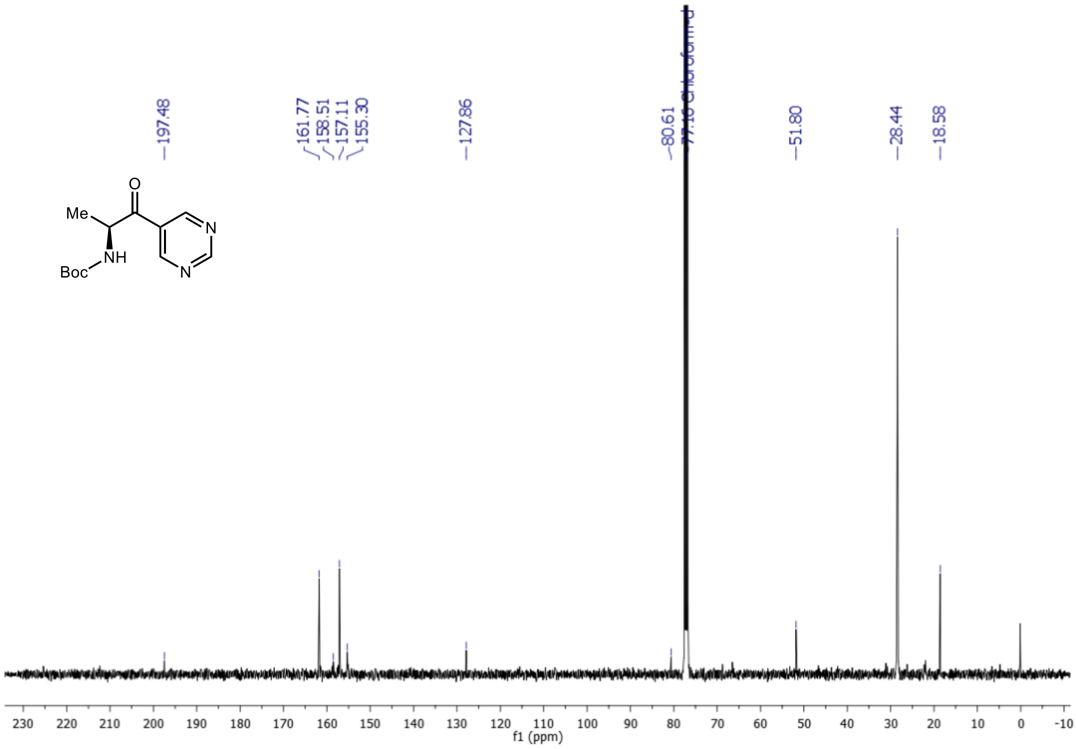

^{13}C NMR (126 MHz, CDCl$_3$) of compound 3k.

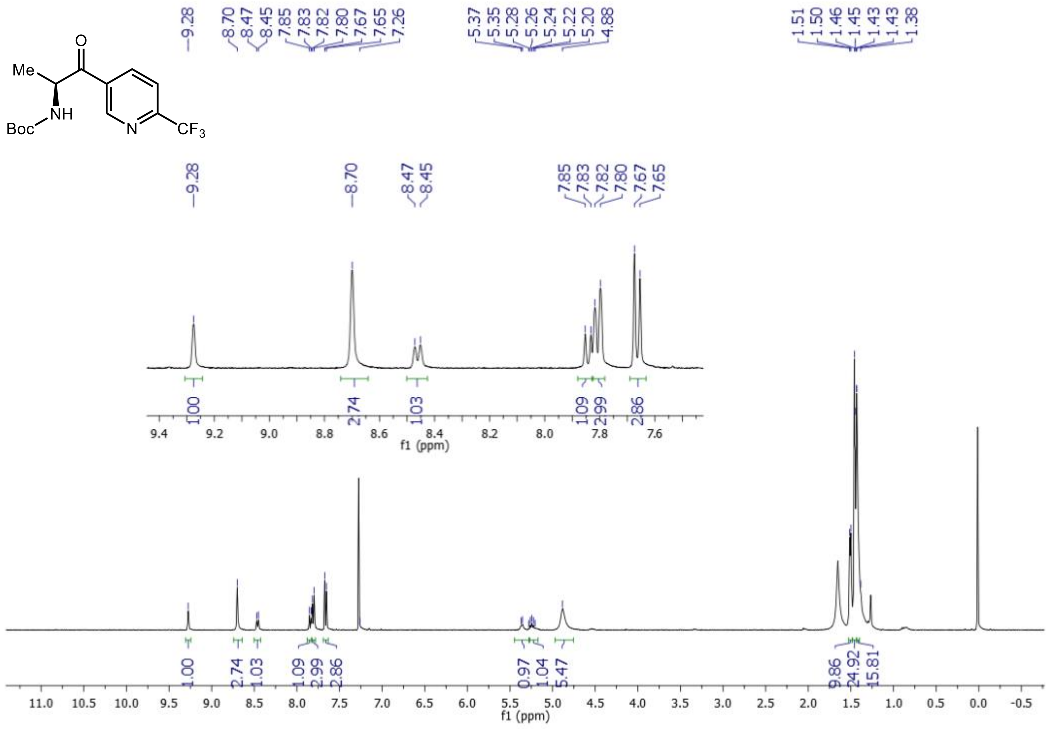

^{1}H NMR (400 MHz, CDCl$_3$) of compounds 3l+ 4l.

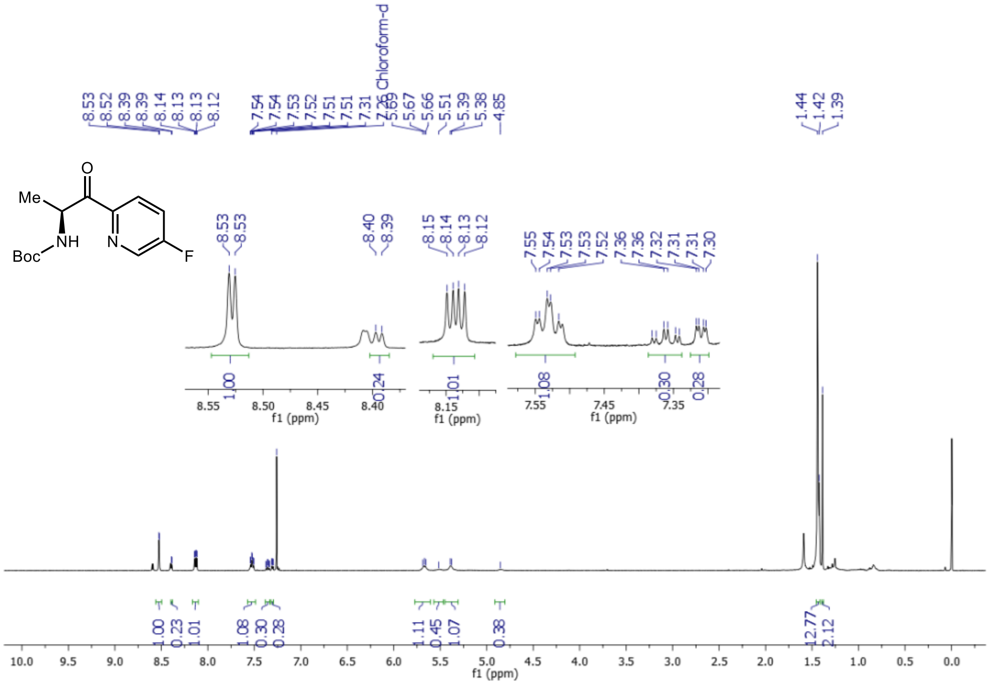

^{1}H NMR (500 MHz, CDCl$_3$) of compounds 3m + 4m.

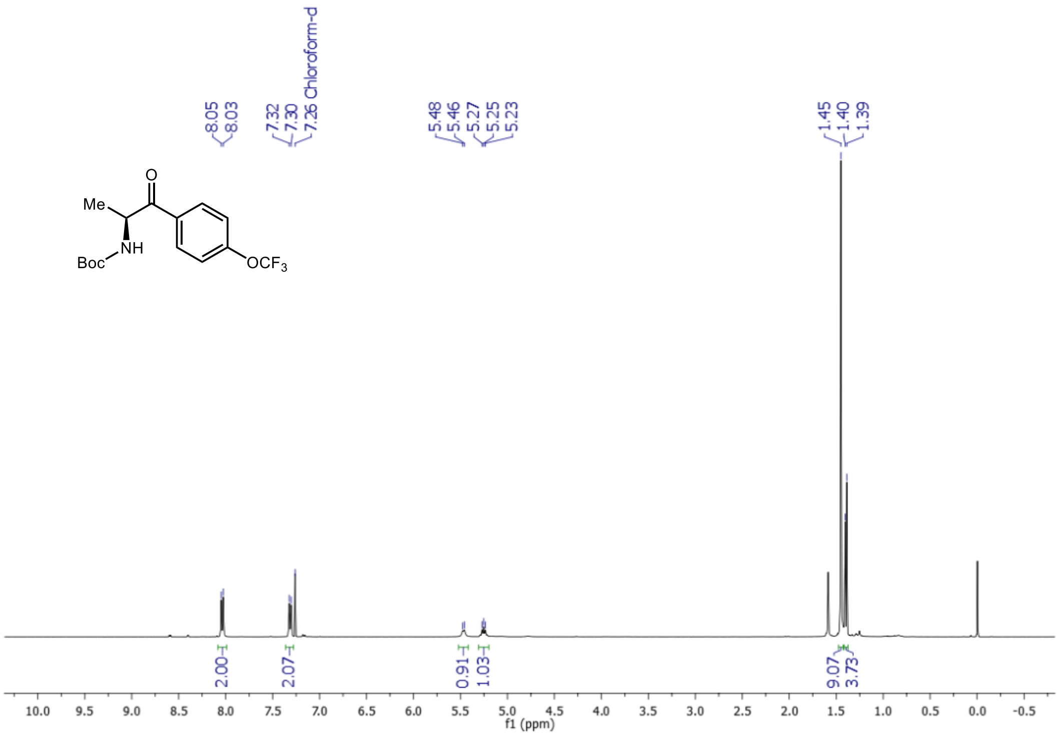

^{1}H NMR (500 MHz, $CDCl_3$) of compound 3n.

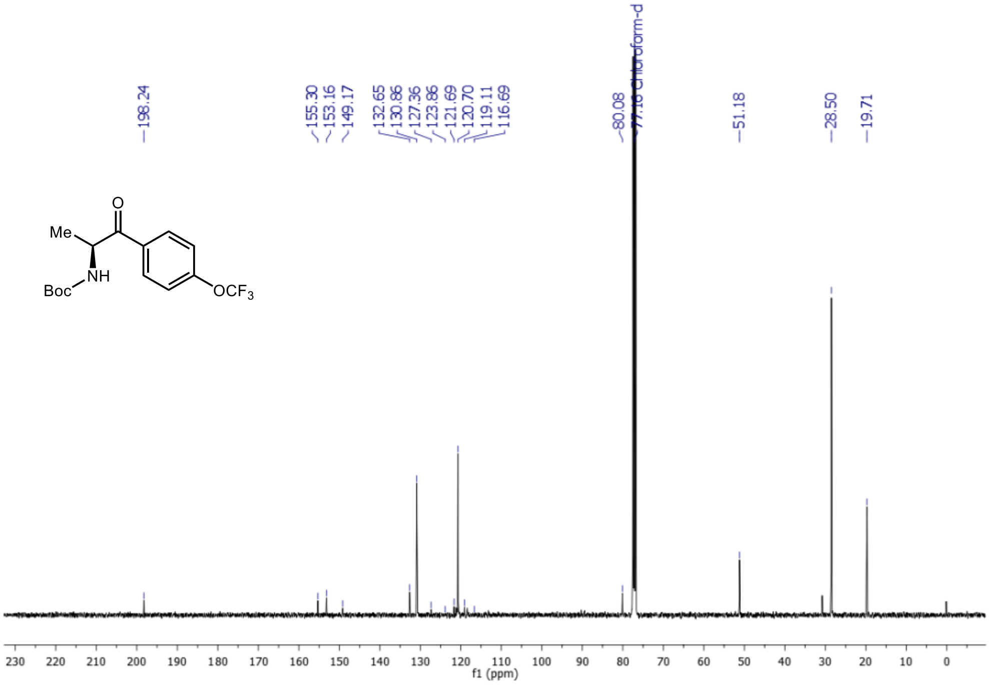

^{13}C NMR (126 MHz, $CDCl_3$) of compound 3n.

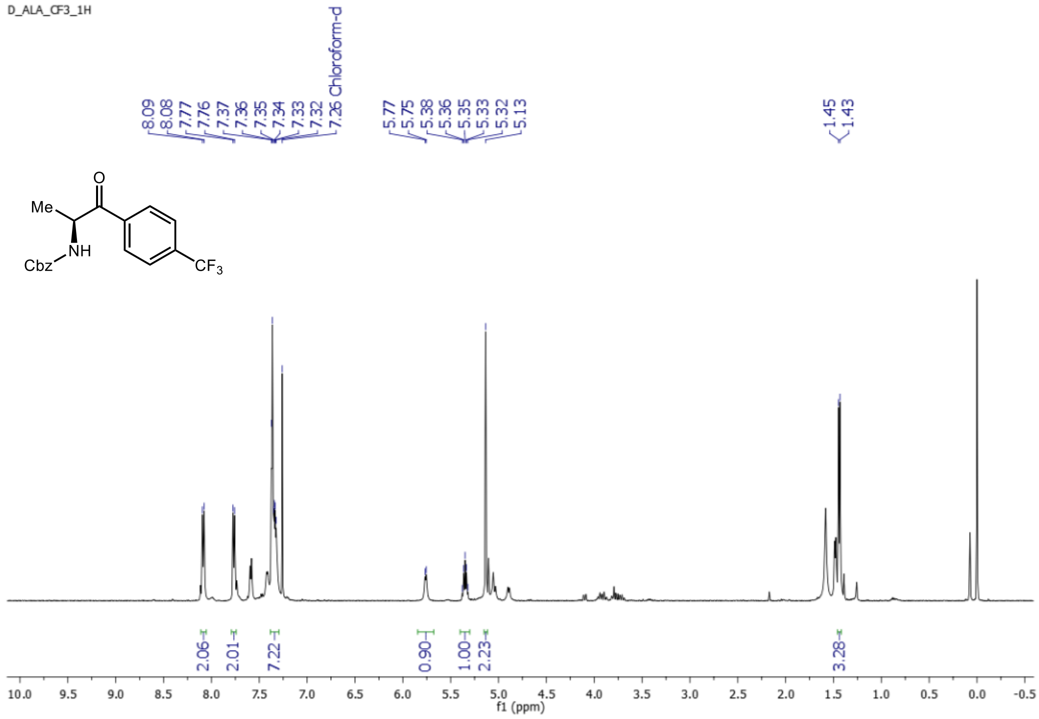

^{1}H NMR (500 MHz, $CDCl_3$) of compounds 3p + 4p.

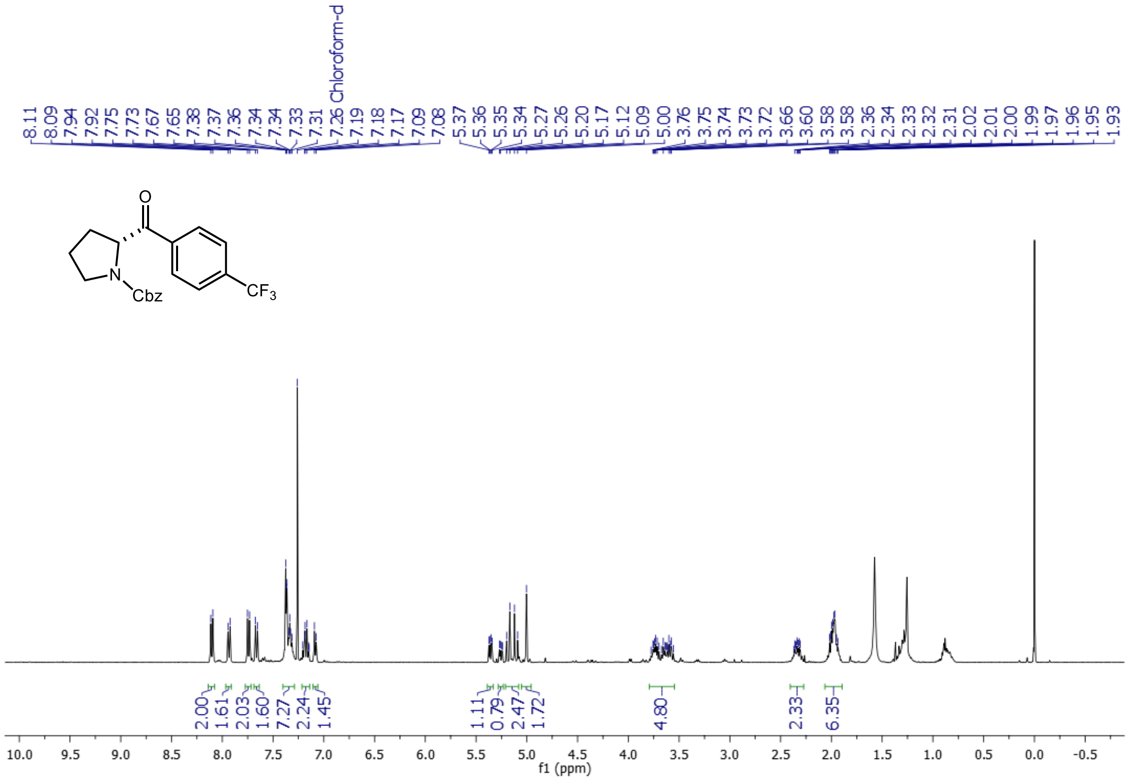

^{1}H NMR (500 MHz, $CDCl_3$) of compounds 3r + 4r.

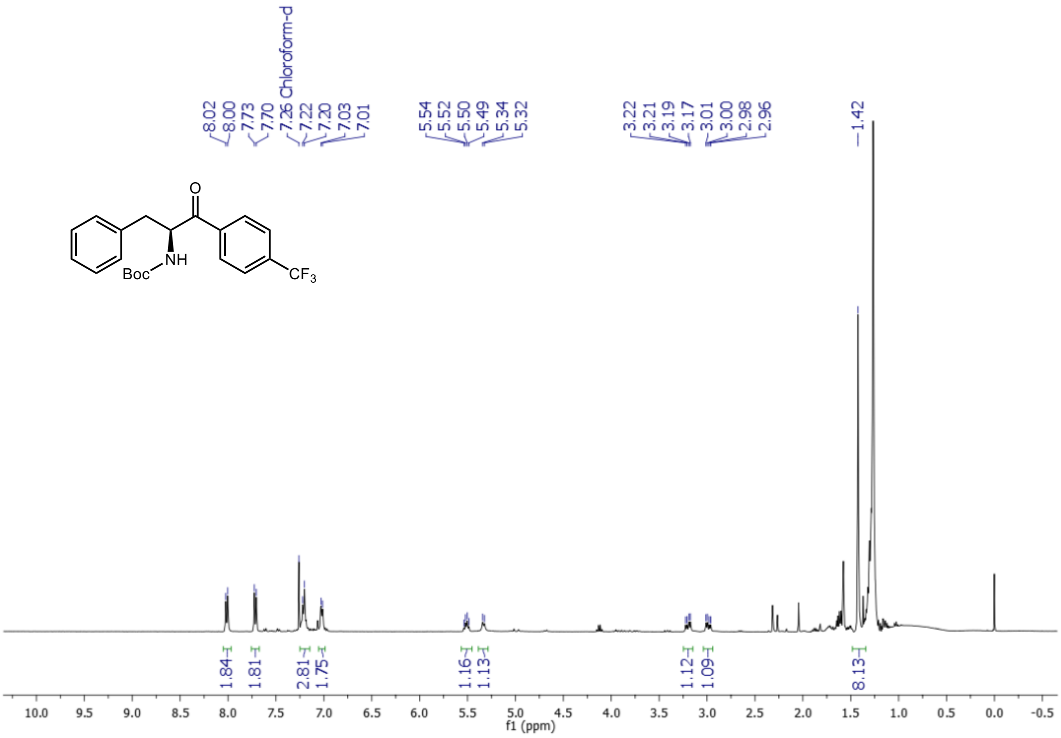

^{1}H NMR (500 MHz, $CDCl_3$) of compound 3s.

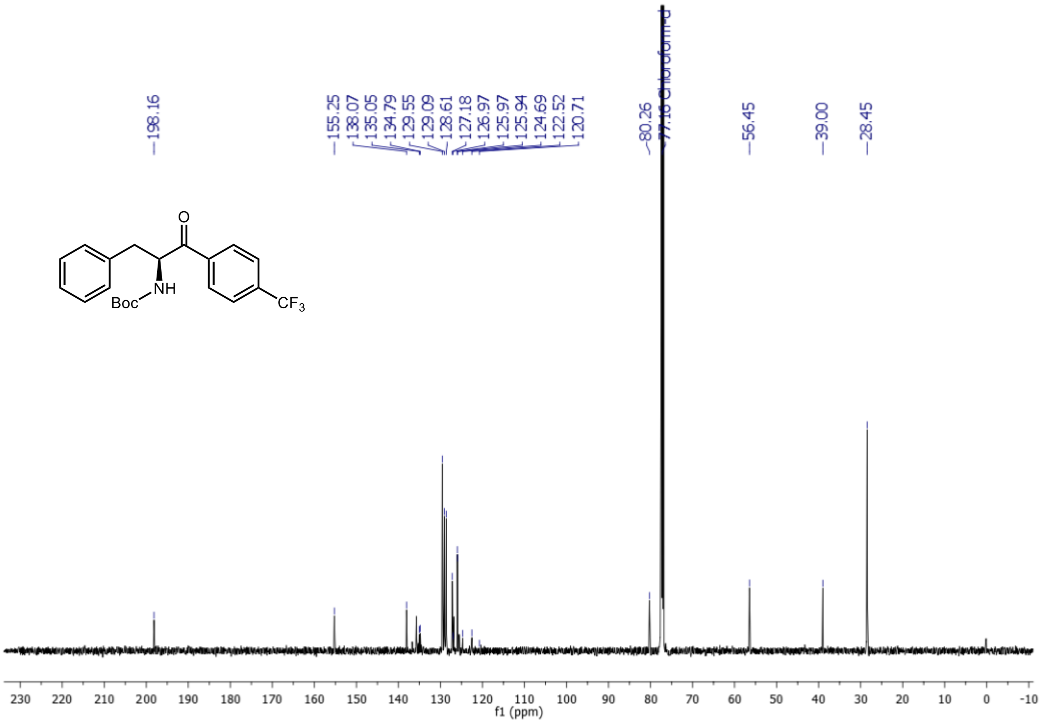

^{13}C NMR (126 MHz, $CDCl_3$) of compound 3s.

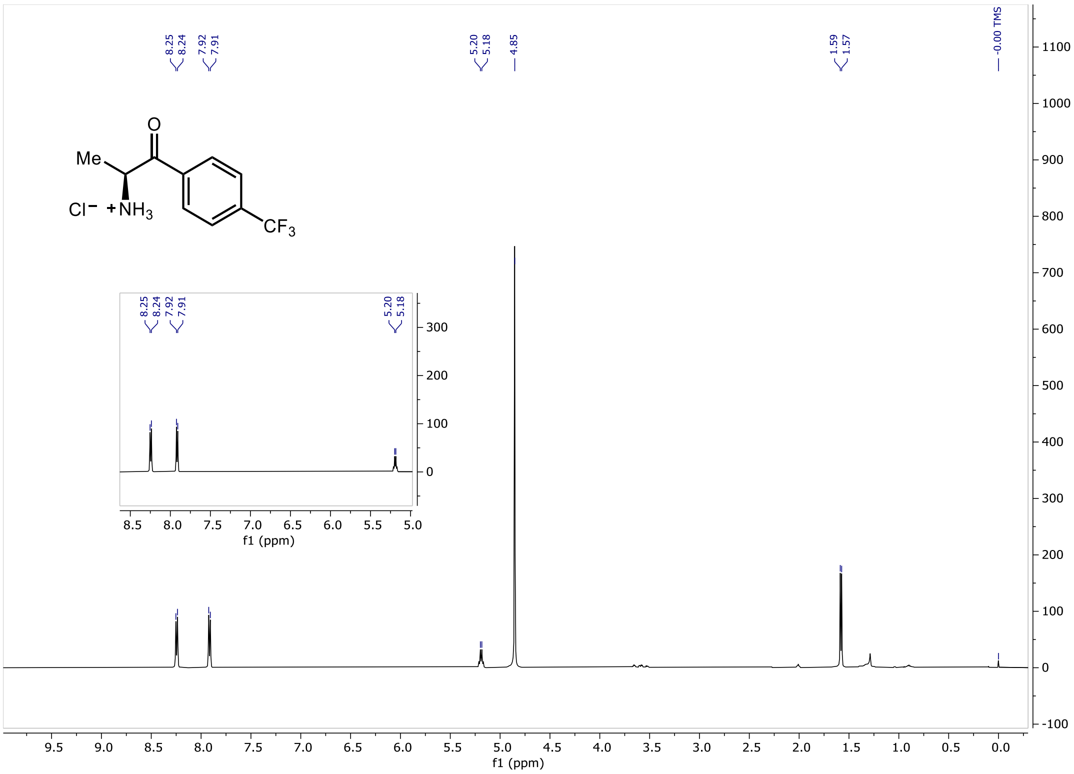

^{1}H NMR (500 MHz, D_2O) of compound 5c.

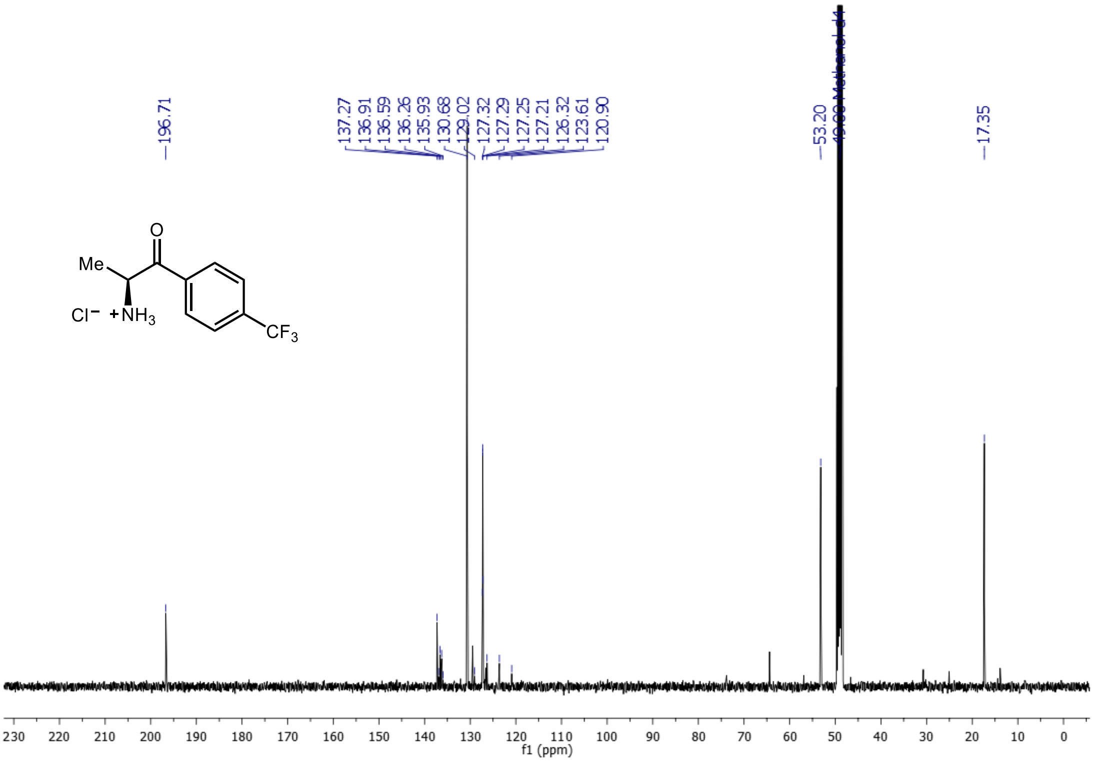

^{13}C NMR (126 MHz, CD_3OD) of compound 5c.

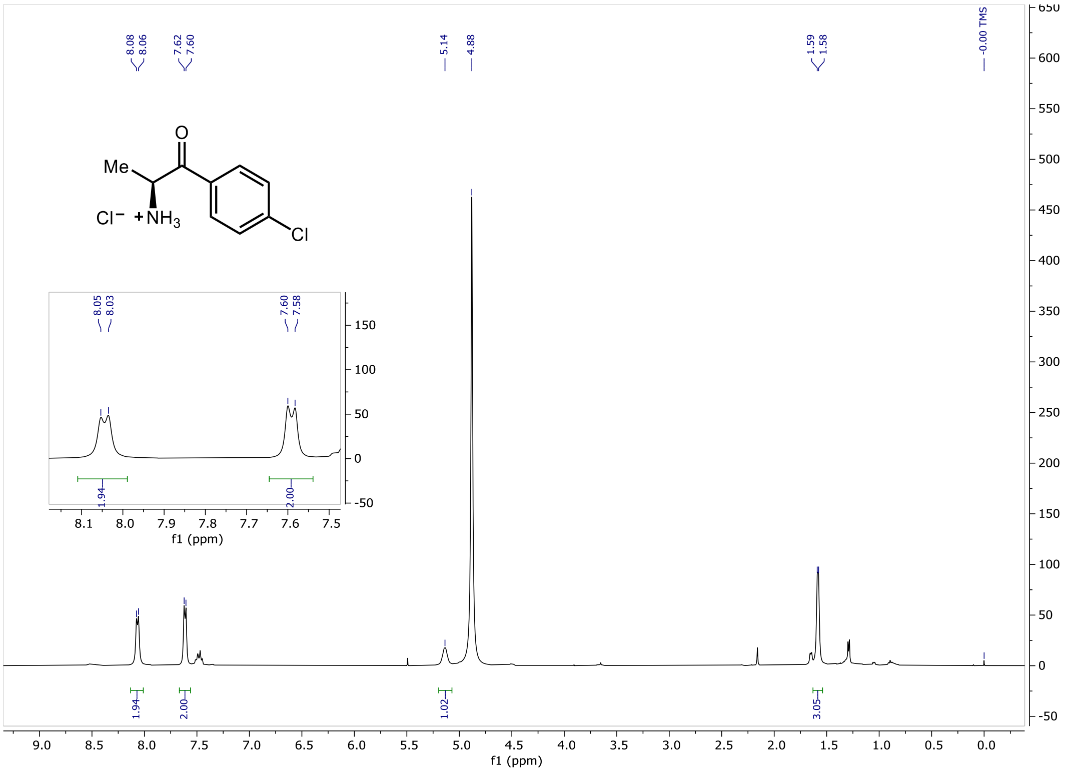

^{1}H NMR (500 MHz, D_2O) of compound 5d.

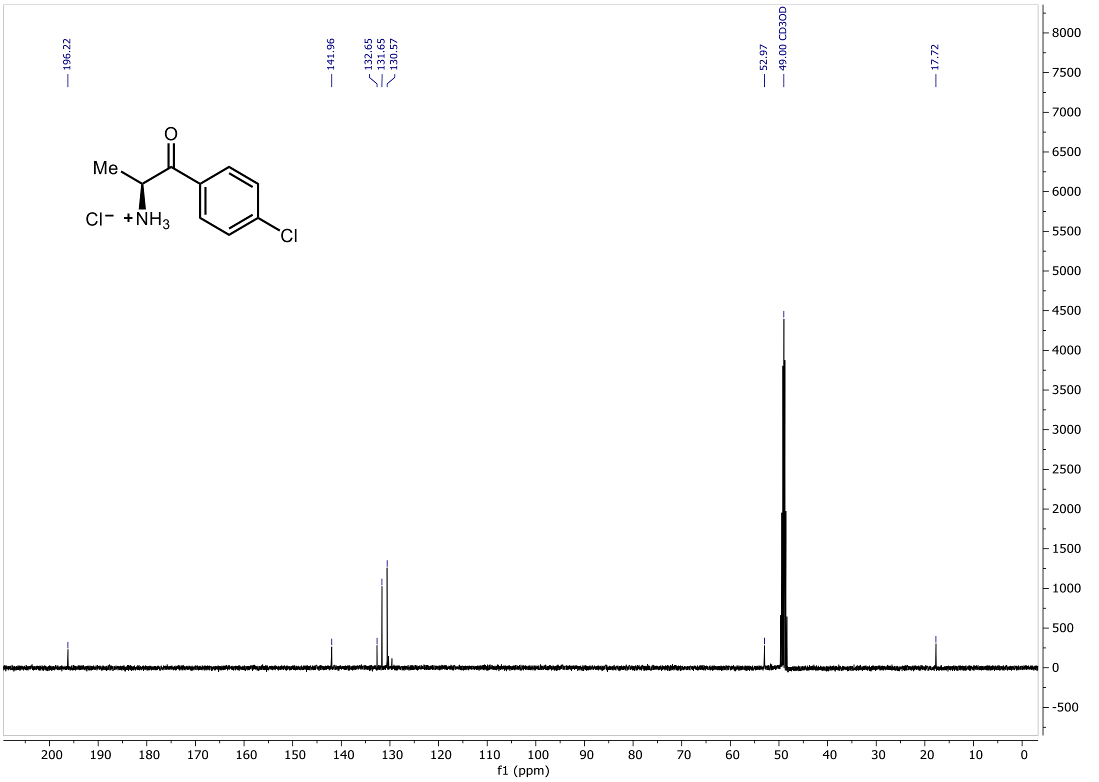

^{13}C NMR (126 MHz, CD_3OD) of compound 5d.

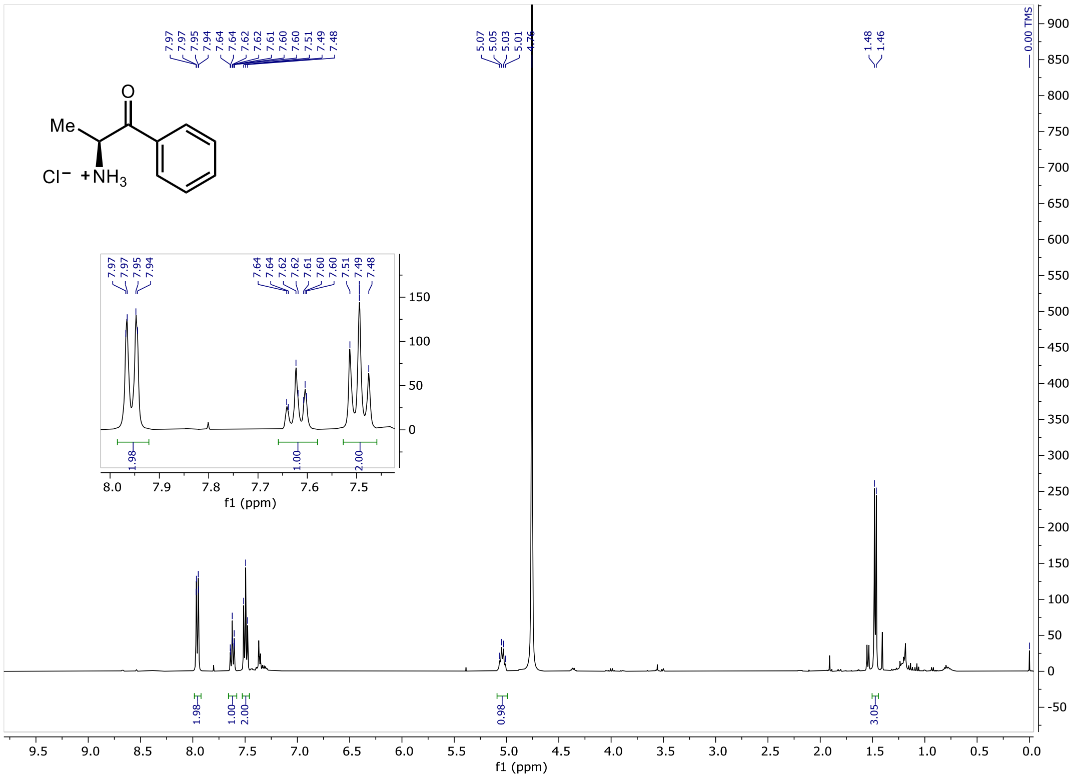

^{1}H NMR (500 MHz, D_2O) of compound 5g.

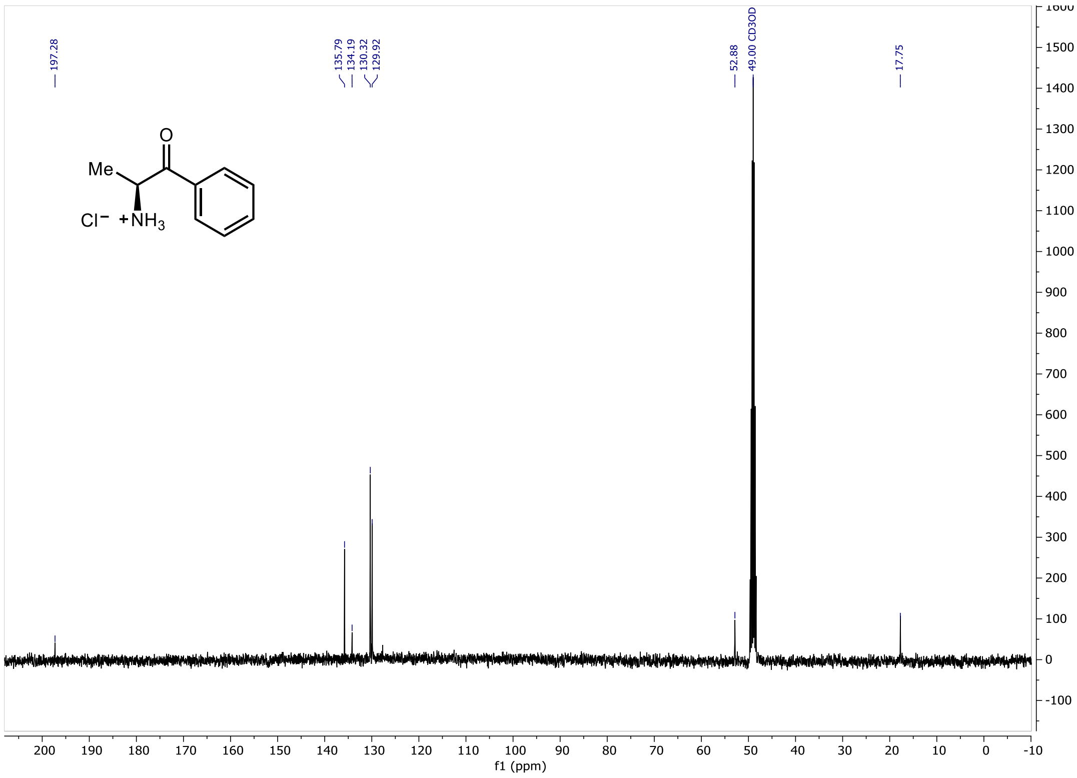

^{13}C NMR (126 MHz, CD_3OD) of compound 5g.
